# Transcriptomics analysis reveals new insights in E171-induced molecular alterations in a mouse model of colon cancer

**DOI:** 10.1038/s41598-018-28063-z

**Published:** 2018-06-27

**Authors:** Héloïse Proquin, Marlon J. Jetten, Marloes C. M. Jonkhout, Luis Guillermo Garduño-Balderas, Jacob J. Briedé, Theo M. de Kok, Henk van Loveren, Yolanda I. Chirino

**Affiliations:** 1 Department of Toxicogenomics, GROW institute of Oncology and Developmental Biology, Maastricht University, Maastricht, The Netherlands; 2 Laboratorio de Carcinogénesis y Toxicología, Unidad de Biomedicina, FES-Iztacala, UNAM, Estado de México, Mexico; 3 IUF-Leibniz Research Institute for Environmental Medicine, Auf’m Hennekamp 50, 40225 DE Düsseldorf, Germany

## Abstract

Titanium dioxide as a food additive (E171) has been demonstrated to facilitate growth of chemically induced colorectal tumours *in vivo* and induce transcriptomic changes suggestive of an immune system impairment and cancer development. The present study aimed to investigate the molecular mechanisms behind the tumour stimulatory effects of E171 in combination with azoxymethane (AOM)/dextran sodium sulphate (DSS) and compare these results to a recent study performed under the same conditions with E171 only. BALB/c mice underwent exposure to 5 mg/kg_bw_/day of E171 by gavage for 2, 7, 14, and 21 days. Whole genome mRNA microarray analyses on the distal colon were performed. The results show that E171 induced a downregulation of genes involved in the innate and adaptive immune system, suggesting impairment of this system. In addition, over time, signalling genes involved in colorectal cancer and other types of cancers were modulated. In relation to cancer development, effects potentially associated with oxidative stress were observed through modulation of genes related to antioxidant production. E171 affected genes involved in biotransformation of xenobiotics which can form reactive intermediates resulting in toxicological effects. These transcriptomics data reflect the early biological responses induced by E171 which precede tumour formation in an AOM/DSS mouse model.

## Introduction

Titanium dioxide (TiO_2_) is the most produced mineral worldwide^1^ and is used for its white colouring properties and stability over time^2^. One of the main applications of TiO_2_ is its use as a food additive in processed food such as cookies, sweets, toppings and coffee creamer^1–3^. TiO_2_ was accepted as a food colorant and registered under the code of E171 by the European Union (EU) in 1969^4^. This approval followed a risk assessment made earlier that year by the joint Food Agriculture Organisation and World Health Organisation (FAO/WHO) that identified no risk concerning the ingestion of TiO_2_^4^. Therefore, E171 is permitted by the EU in food at *quantum satis*, which means that there are no maximum intake levels for ingestion. In the USA, the regulation by the food and drug administration limits titanium dioxide to a maximum of 1% of the weight of the food^5^.

Over the past decade, characteristics have been studied in order to assess possible adverse effects. The shape and size of E171 have been measured by several groups. E171 consist of approximately 40% of nanoparticles (NPs) (<100 nm) and 60% of microparticles (MPs) (>100 nm)^3,6,7^. Studies on possible adverse effects have primarily been focused on inhalation of TiO_2_. Based on the outcome of these studies, the International Agency for Research in Cancer (IARC) changed in 2010 the classification of TiO_2_ from non-carcinogenic to probable carcinogenic to humans (Group 2B)^8^.

More recently, there are increasing number of studies to detect and quantify E171 in foods for human consumption^9^, pharmaceutical products^10^, to distinguish between NPs and MPs in foods, and to estimate the daily intake in adults and children^11^. In addition, a number of both *in vivo* and *in vitro* studies report on the adverse effects of exposure to TiO_2_ by the oral route and the biological mechanisms involved. *In vitro* studies have identified induction of DNA damage and oxidative stress by the production of reactive oxygen species (ROS) in colon cell lines such as Caco-2 and HCT116 cells^6,7,12^. Inflammation has also been observed *in vivo* with a significant increase of superoxide anion (O_2_^**·**−^) and hydrogen peroxide (H_2_O_2_) in the liver after ingestion of 10 mg/kg_bw_ of TiO_2_ NPs for 60 days in rats^13^. The same group observed after ingestion of 10 mg/kg_bw_ of TiO_2_ NPs for 90 days an induced inflammation and a reduction of the immune capacity in liver^14^. A more recent study performed by our group showed a significant increase of the number of colonic tumours after ingestion of E171 for 10 weeks in a murine colorectal cancer (CRC) model, in which colorectal tumours were chemically induced by azoxymethane (AOM)/dextran sodium sulphate (DSS)^15^. In addition, we identified that a decrease in number of goblet cells and neoplastic alterations in distal colon of mice began after 4 weeks of exposure to TiO_2_^15^. We also investigated initial transcriptome changes in colon tissue before the neoplastic alterations appeared and found that intragastric exposure to 5 mg/kg_bw_/day of E171 for 2, 7, 14 and 21 days led to the up-regulation of genes involved in activation of inflammation, reduction of immune capacity and up-regulation of genes related in the development of CRC^16^. In line with our data, Bettini *et al*. observed, after ingestion of E171 in rats, a potent Th1/Th17 immune response via an increased production of IFN-γ in Peyer’s Patches and IFN-γ and IL-17 in the spleen after 7 days of exposure^17^. In addition, Bettini *et al*. showed that E171 exposure for 100 days in a chemically induced carcinogenesis model induced a release of inflammatory molecules at a low level in the colon, preneoplastic lesions as well as the growth of aberrant crypt foci.

Based on the previous studies on the effects of E171 in the colon, we raised the hypothesis that ingestion of E171 induces gene expression changes in colon that are related to inflammation, deregulation of cancer-related genes and impairment of the immune system before tumours are detectable. To test this hypothesis, mice were co-exposed to AOM/DSS and to 5 mg/kg_bw_/day of E171 for 2, 7, 14 and 21 days and transcriptome changes in the colon were determined by whole genome mRNA microarrays.

## Results

### Differentially expressed genes (DEG)

**Figure 1:**
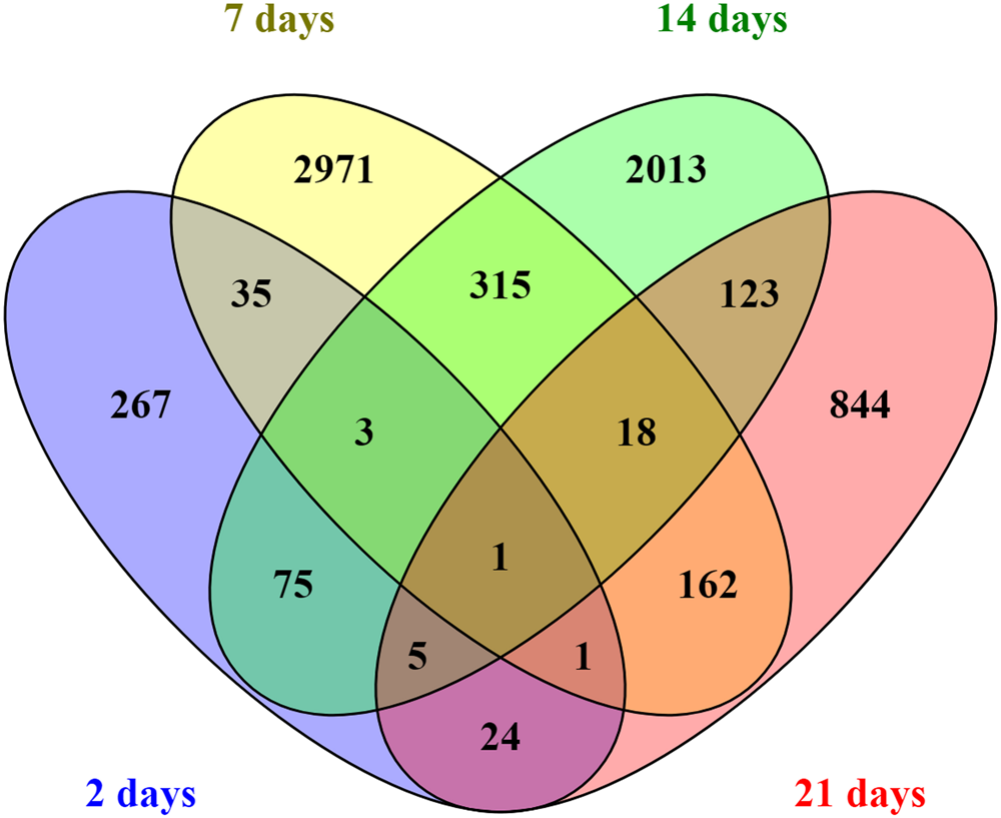
Venn diagram of the overlap (not based on directionality of expression) of DEGs (p < 0.05 and Log2FC > 1.5) at different time points (2, 7, 14, and 21 days) after exposure to E171 in colon of mice. Each colour represents a different day.

Gene expression values from samples of the distal colon of mice exposed to E171 (CRC + E171) were corrected for their own time-matched control (CRC) samples. In total, 21,106 genes passed the pre-processing. After using a Linear Mixed Model Analysis for Microarrays (LIMMA), the genes with a Log2FC > 1.5 and a p < 0.05 were selected. This selection resulted in 411 DEG after 2 days, 3506 DEG after 7 days of exposure, 2553 DEG after 14 days and 1178 DEG after 21 days (Table 1). With an adjusted p-value also called false discovery rate (FDR), there were only 2 DEG after 21 days and none after 2 days. Therefore, analysis will be performed with the DEG having a Log2FC > 1.5 and a p < 0.05 and stronger effects will be highlighted by indicating in the DEG with a Log2FC > 1.5 and a p < 0.05 which ones were also significant after FDR correction. The majority of changes in gene expression were time-point specific (Fig. 1). From day 2 to 21 between 10 and 30% of the DEG were common to the other time point(s).

**Table 1:** Summary results of DEG after LIMMA analysis.

	2 days	7 days	14 days	21 days
|FC|> = 1.5	2128	5277	4671	3387
Up-regulated	1203	2567	1888	616
Down-regulated	925	2710	2783	2771
p.val < 0.05	771	5106	3622	1951
adj.p.val < 0.05	0	1532	246	2
|FC| and p.val	411	3506	2553	1178
|FC| and adj.p.val	0	1390	233	2

Differently Expressed Genes after LIMMA analysis on the microarray data of chemically induced CRC BALB/c mice exposed to 5 mg/kg bw/day of E171. |FC| = Log2 fold change, p.val = p-value, adj.p.val = adjusted p-value.

Figure 1 also shows DEG in common between different time points. In total, 27 DEG were in common in 3 out of 4 time points. To visualise the expression of these DEG, a heatmap was created (see Supplementary Fig. 1). Among the 27 DEG common in 3 out of 4 time points, 8 had an unknown function. The other 19 DEG were separated according to their function in 3 clusters: cellular processes (7 DEG), gene involved in cancer (6 DEG) and genes coding for olfactory/G-protein-coupled receptors (GPCR) receptors (6 DEG). The DEG common to all time points was in the olfactory/GPCR receptors cluster in the heatmap: olfactory gene 975. It was up-regulated at 2, 14 and 21 days and down-regulated after 7 days of exposure.

Three DEG were also statistically significant after FDR correction: Npc1 in cellular processes and Ifitm10 and Sox17 in genes involved in cancer. These DEG were shown with a black square around in the heat map.

### Pathway analysis per time point

With regard to the pathway analyses applied in the previous study^16^, the affected biological mechanisms per time point were studied using the Consensus Pathway Database (CPDB) database for 2 different pathway analyses: over-representation gene set analysis (ORA) and a gene-set enrichment analysis (GSEA). The ORA resulted in 4 pathways after 2 days, the number of pathways increased at day 7 up to a total of 67 pathways and decreased after 14 and 21 days with 40 and 12 pathways respectively (Table 2). In addition, the GSEA resulted in 1 pathway at 2 days, 37 at day 7, 17 at day 14, and 107 at day 21 (see Supplementary Table 5). More details about the genes and their direction of expression can be found in Supplementary Tables 1, 2, 3 and 4.

**Table 2:** Summary table with pathways, number of genes and their direction.

Days of exposure	Group of pathways	Pathways	q-value	pathway source	Down	Up	Total
2 days	Signal transduction	Olfactory transduction	5.10E-12	KEGG	5	26	31
		Olfactory signalling Pathway	4.61E-05	Reactome	5	10	15
		GPCR downstream signalling	0.0183	Reactome	8	13	21
		Signalling by GPCR	0.0347	Reactome	8	14	22
7 days	Signal transduction	Class A/1 (Rhodopsin-like receptors)	2.42E-03	Reactome	45	21	66
		GPCR ligand binding	3.86E-03	Reactome	56	23	79
		Peptide ligand-binding receptors	1.56E-02	Reactome	28	14	42
		G alpha (s) signalling events	1.63E-02	Reactome	18	9	27
		Non-odorant GPCRs	3.06E-02	Wikipathways	36	19	55
		Antagonism of Activin by Follistatin	2.42E-02	Reactome	4	0	4
	Xenobiotics metabolism	Phase 1 - Functionalization of compounds	6.41E-04	Reactome	6	24	30
		Biological oxidations	8.93E-05	Reactome	8	39	47
		Xenobiotics	7.40E-04	Reactome	1	15	16
		Cytochrome P450 - arranged by substrate type	5.75E-03	Reactome	3	20	23
		bupropion degradation	5.75E-03	MouseCyc	1	13	14
		nicotine degradation III	7.23E-03	MouseCyc	1	14	15
		Metabolism of xenobiotics by cytochrome P450	7.54E-03	KEGG	3	23	26
		Drug metabolism - cytochrome P450	8.75E-03	KEGG	2	23	25
		nicotine degradation II	9.60E-03	MouseCyc	2	14	16
		Drug metabolism - other enzymes	2.31E-02	KEGG	1	16	17
	Metabolism	Nuclear receptors in lipid metabolism and toxicity	1.04E-02	Wikipathways	4	9	13
		Prostaglandin Synthesis and Regulation	2.42E-02	Wikipathways	10	3	13
		Synthesis of epoxy (EET) and dihydroxyeicosatrienoic acids (DHET)	8.37E-03	Reactome	0	10	10
		Synthesis of (16–20)-hydroxyeicosatetraenoic acids (HETE)	3.67E-02	Reactome	0	10	10
		Ascorbate and aldarate metabolism	2.53E-02	KEGG	0	9	9
		Linoleic acid metabolism	2.24E-02	KEGG	2	14	16
		Arachidonic acid metabolism	6.41E-04	Reactome	18	23	41
		Synthesis of Prostaglandins (PG) and Thromboxanes (TX)	1.83E-02	Reactome	5	5	10
		Propanoate metabolism	4.98E-02	KEGG	1	11	12
		UDP-N-acetyl-D-glucosamine biosynthesis II	2.33E-02	MouseCyc	1	4	5
	Metabolism of proteins	Regulation of Insulin-like Growth Factor (IGF) Transport and Uptake by Insulin-like Growth Factor Binding Proteins (IGFBPs)	3.86E-03	Reactome	5	9	14
		O-linked glycosylation of mucins	9.60E-03	Reactome	3	13	16
		Mucin type O-Glycan biosynthesis	1.63E-03	KEGG	4	10	14
	Immune response	Cytokine-cytokine receptor interaction	5.56E-04	KEGG	54	13	67
		Complement and coagulation cascades	9.60E-03	KEGG	18	3	21
		Staphylococcus aureus infection	4.98E-02	KEGG	17	0	17
	Cancer signalling	Chemical carcinogenesis	9.60E-03	KEGG	3	22	25
		FGFR3c ligand binding and activation	4.98E-02	Reactome	2	3	5
		FGFR3 ligand binding and activation	4.98E-02	Reactome	2	3	5
		Signalling by activated point mutants of FGFR3	4.98E-02	Reactome	2	3	5
		Signalling by FGFR3 mutants	4.98E-02	Reactome	2	3	5
		FGFR1c ligand binding and activation	4.98E-02	Reactome	2	3	5
		Signalling by activated point mutants of FGFR1	4.98E-02	Reactome	2	3	5
	Haemostasis	Common Pathway of Fibrin Clot Formation	4.13E-02	Reactome	6	1	7
		Cell surface interactions at the vascular wall	2.26E-02	Reactome	18	8	26
	Extracellular matrix organisation	Extracellular matrix organization	1.84E-07	Reactome	67	8	75
		Degradation of the extracellular matrix	3.98E-05	Reactome	26	5	31
		Activation of Matrix Metalloproteinases	8.93E-05	Reactome	17	0	17
		Matrix Metalloproteinases	3.86E-03	Wikipathways	12	1	13
		Elastic fibre formation	2.42E-02	Reactome	12	1	13
		Collagen formation	3.69E-02	Reactome	21	1	22
		Assembly of collagen fibrils and other multimeric structures	3.83E-02	Reactome	11	1	12
	Digestive system	Bile secretion	1.36E-03	KEGG	6	20	26
		Pancreatic secretion	8.76E-03	KEGG	15	16	31
		Amino sugar and nucleotide sugar metabolism	1.03E-02	KEGG	3	16	19
		Protein digestion and absorption	9.60E-03	KEGG	16	12	28
		Mineral absorption	6.41E-04	KEGG	8	13	21
7 days	Endocrine and metabolic disease	Maturity onset diabetes of the young	3.86E-03	KEGG	0	13	13
	Muscle contraction	Striated Muscle Contraction	4.07E-04	Reactome	15	1	16
		Muscle contraction	4.29E-02	Reactome	15	2	17
	Bone development	Endochondral Ossification	9.68E-04	Wikipathways	20	5	25
		Endocrine and other factor-regulated calcium reabsorption	2.17E-02	KEGG	5	14	19
		Calcium signalling pathway	2.33E-02	KEGG	26	20	46
	Transport of molecules	Transport of glucose and other sugars, bile salts and organic acids, metal ions and amine compounds	8.76E-03	Reactome	12	17	29
		Ion channel transport	2.11E-02	Reactome	12	23	35
		Ion transport by P-type ATPases	2.26E-02	Reactome	7	7	14
		Organic cation transport	4.98E-02	Reactome	1	4	5
		Transmembrane transport of small molecules	7.00E-03	Reactome	41	69	110
14 days	Signal transduction	Olfactory transduction	1.57E-44	KEGG	77	72	149
		GPCR downstream signalling	5.28E-26	Reactome	97	60	157
		Signalling by GPCR	8.59E-25	Reactome	106	63	169
		Olfactory signalling Pathway	6.18E-24	Reactome	43	36	79
		Signal Transduction	2.50E-10	Reactome	143	82	225
		Odorant GPCRs	2.51E-09	Wikipathways	24	19	43
		GPCR ligand binding	5.85E-07	Reactome	44	23	67
		GPCRs, Class A Rhodopsin-like	1.51E-06	Wikipathways	23	19	42
		GPCRs, Other	1.80E-06	Wikipathways	18	12	30
		Non-odorant GPCRs	3.08E-06	Wikipathways	34	17	51
		Class A/1 (Rhodopsin-like receptors)	4.40E-04	Reactome	32	16	48
		G alpha (i) signalling events	4.05E-03	Reactome	24	11	35
		Taste transduction	9.95E-03	KEGG	8	4	12
		Monoamine GPCRs	2.96E-02	Wikipathways	4	5	9
		G alpha (s) signalling events	3.12E-02	Reactome	13	6	19
		G alpha (q) signalling events	4.20E-02	Reactome	18	9	27
		Class C/3 (Metabotropic glutamate/pheromone receptors)	4.05E-03	Reactome	5	4	9
		Amine ligand-binding receptors	3.45E-02	Reactome	5	3	8
	Xenobiotics metabolism	Phase 1 - Functionalization of compounds	1.60E-02	Reactome	3	16	19
		Biological oxidations	6.38E-03	Reactome	4	25	29
		Drug metabolism - other enzymes	2.43E-02	KEGG	2	11	13
		Drug metabolism - cytochrome P450	3.12E-02	KEGG	3	14	17
		Cytochrome P450 - arranged by substrate type	3.82E-02	Reactome	1	14	15
	Metabolism	Steroid hormone biosynthesis	6.38E-03	KEGG	4	4	8
		Steroid hormones	2.73E-02	Reactome	3	10	13
		Retinol metabolism	1.08E-04	KEGG	2	18	20
		Ascorbate and aldarate metabolism	1.72E-03	KEGG	2	7	9
		Vitamin D (calciferol) metabolism	6.67E-03	Reactome	1	3	4
		Metabolism of steroid hormones and vitamin D	2.73E-02	Reactome	4	4	8
	Cancer signalling	Chemical carcinogenesis	1.78E-02	KEGG	4	14	18
	Digestive system	Recycling of bile acids and salts	4.98E-03	Reactome	2	5	7
		Fatty acid degradation	1.98E-02	KEGG	1	12	13
		Linoleic acid metabolism	2.96E-02	KEGG	3	9	11
		Bile secretion	3.45E-02	KEGG	3	13	16
		Fatty acids	3.86E-02	Reactome	0	6	6
	Transport of molecules	Transmembrane transport of small molecules	4.20E-03	Reactome	35	41	76
		SLC-mediated transmembrane transport	3.12E-02	Reactome	15	26	41
		Transport of vitamins, nucleosides, and related molecules	3.45E-02	Reactome	4	6	10
	Neuronal response	Neuroactive ligand-receptor interaction	4.55E-06	KEGG	28	24	52
		Serotonin receptors	4.37E-02	Reactome	3	1	4
21 days	Signal transduction	Olfactory transduction	3.02E-02	KEGG	29	5	34
		G Protein signalling Pathways	4.31E-02	Wikipathways	13	0	13
	Immune response	Cell adhesion molecules (CAMs)	8.19E-04	KEGG	22	0	22
		Innate Immune System	4.75E-03	Reactome	41	0	41
		Immune System	5.76E-03	Reactome	63	0	63
	Extracellular matrix	Extracellular matrix organization	1.51E-02	Reactome	23	0	23
		Regulation of actin cytoskeleton	3.53E-02	KEGG	21	0	21
	Neuronal response	Neuronal System	5.76E-03	Reactome	24	1	25
		Transmission across Chemical Synapses	1.50E-02	Reactome	18	1	19
		Serotonin and anxiety-related events	2.73E-02	Wikipathways	4	0	4
		Neurotransmitter Receptor Binding And Downstream Transmission In The Postsynaptic Cell	4.31E-02	Reactome	14	0	14
		HCN channels	3.02E-02	Reactome	3	0	3

Pathways related to the DEG after ORA with ConsensusPathDB. The pathways were grouped per biological function. The q-value is obtained after correction of the p-values for multiple testing using the false discovery rate method.

Because we raised the hypothesis that ingestion of E171 induces gene expression changes in colon which could be related to inflammation, cancer-related genes and impair of the immune system before the tumours are detectable, the focus of the result section will be on inflammation, immune response, and cancer related pathways.

#### 2 days of exposure

**Figure 2:**
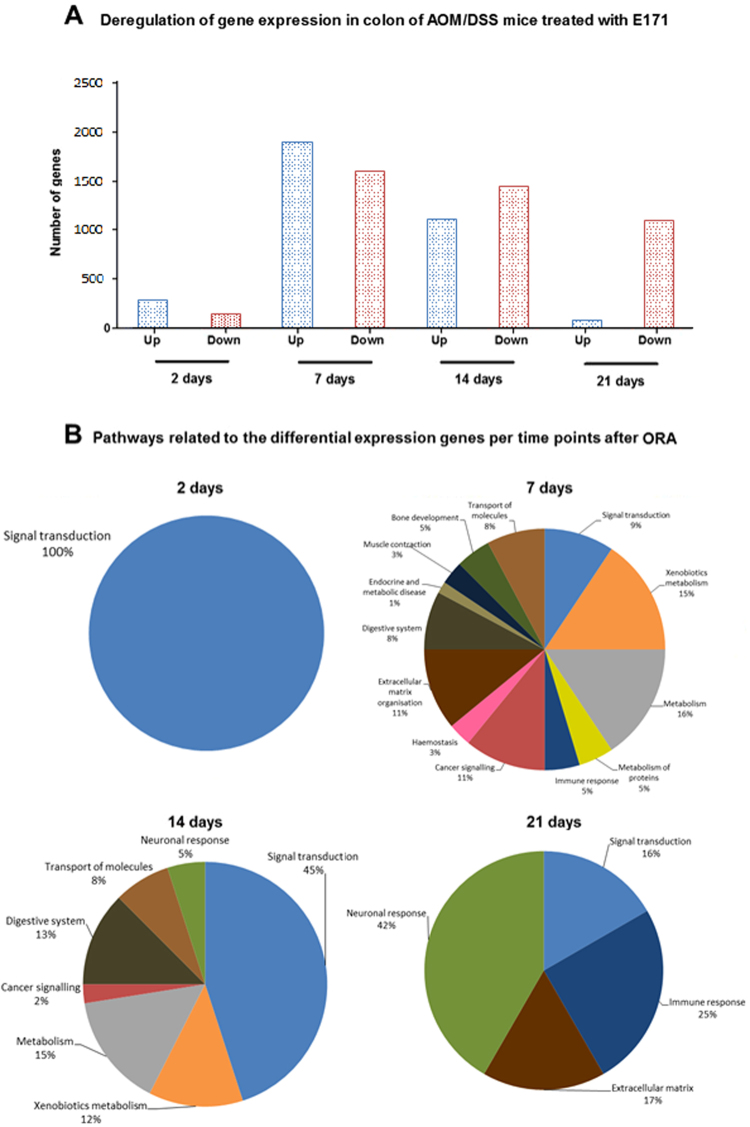
Visualisation of genes and pathways affected after E171 exposure. (**A**) Up and down regulation of expression of genes; (**B**) percent of group of pathways derived from the over-representation analysis (ORA) from colon tissue of mice treated with AOM/DSS and exposed to E171 during 2, 7, 14 and 21 days.

ORA and GSEA showed that all DEG expressed after 2 days were related to only one biological function, olfactory/GPCR signalling. ORA resulted in pathways such as olfactory signalling and GPCR signalling pathways (Table 2 and Fig. 2) whereas the GSEA resulted only in the olfactory transduction pathway (see Supplementary Table 5). In all pathways, 66% of the genes were up-regulated. In these pathways 6 genes were related to cancer like pancreas cancer (Ramp1 and Cckbr)^16,18,19^, melanoma (Plcb4)^20^, cervical and ovarian (Adcyap1 and Trpc3)^21,22^, and colorectal cancer (Ptger3)^23^.

No pathways related to the immune system were found but at the gene level 20 DEG were found to be related to the immune system including the innate immune system (10 DEG), the adaptive immune system (3 DEG), and cytokine signalling (12 DEG).

#### 7 days of exposure

At day 7 of exposure, the results after ORA showed that more pathways were modulated as compared to 2 days (Table 2 and Fig. 2). Based on the Reactome classification, the 64 pathways were organised according to their biological functions. The number of DEG related to the transport of molecules was the highest (110 DEG) followed by metabolism (109 DEG), signalling (79 DEG), extracellular matrix (75 DEG), and immune response (67 DEG) (see Supplementary Table 2).

Table 2 and Fig. 2 show that gene expression changes were observed in 13 different biological functions: signal transduction, immune system, metabolism, xenobiotics metabolism, muscle contraction, cancer signalling, metabolism of protein, transport of small molecules, extracellular matrix organisation, endocrine and metabolic disease, bone development, haemostasis, and digestive system. After ORA, eight of these biological functions were also observed related to the DEG significant after FDR correction: signal transduction, immune system, metabolism, xenobiotics metabolism, cancer, transport of small molecules, bone development, and digestive system.

Almost 80 DEG were related to signal transduction and clustered in 6 different pathways. A majority of these genes were down-regulated. Some of these genes were known to be related to development of cancer such as neuroblastoma (Ptdgr2)^24^, diverse types of cancer (Kiss1, Ace)^25,26^, breast cancer (Esr1), and colon cancer (Edn2: also significant after FDR correction)^27^.

With 109 DEG, another biological function was identified after ORA: metabolism. The number of metabolism pathways was high therefore a separation was made between metabolism (51 DEG) and biotransformation of xenobiotics (58 DEG) (Table 2 and Fig. 2). These 2 groups of pathways contained an important part of all the DEG associated in pathways after 7 days of exposure. Supplementary Fig. 2 shows the 2 different phases of biotransformation of compounds including up- or down-regulated related DEG for all time points. Phase I reactions exists of oxidation, reduction and hydrolysis. Oxidation of xenobiotics is performed by several mechanisms: CYP450 monooxygenase system, flavin-containing monooxygenase system, alcohol dehydrogenase, monoamine oxidase, co-oxidation by peroxidase^28^. After 7 days, DEG were involved in various biotransformation pathways in Phase 1 and Phase 2. Furthermore, different classes of Cyp450 genes were activated such as Cyp2, Cyp3, Cyp4 and Cyp7. Of all the Cyp450 genes modulated after 7 days of exposure, 18 were also significant after FDR correction. These are shown with top left back square on Supplementary Fig. 2 Other DEG in biotransformation of xenobiotics were at around 80% up-regulated, for instance the glutathione genes Gstt2 and Gstt3 (Also significant after FDR correction).

In the metabolism group, genes in various pathways were modulated such as synthesis of prostaglandins and synthesis of epoxy acids. Synthesis of epoxy acids was also a pathway observed from the DEG with after FDR correction. As shown in Fig. 2, most of these genes were significantly up-regulated.

Another biological function group, i.e. extracellular matrix organisation, was identified after 7 days of E171 administration. It contained pathways involved in collagen formation, activation of the matrix metalloproteinases, and elastic fibre formation. Ninety percent of the genes were down-regulated in this group such as integrin β1 and α2. Twenty-six genes significant after FDR correction were associated with the extracellular matrix organisation.

The immune response function group contained 3 pathways with 41 DEG mostly related to the innate immune response. The majority of the genes were down-regulated like genes involved in the complement activation (C1, C3, C4 and C5), Fc receptor, and interleukins. After FDR correction, 1 C5 gene, 1 C1 gene, 4 Fc receptor genes, and 10 interleukin genes were observed.

Genes involved in cancer and cancer signalling were clustered in 7 pathways. In these pathways 31 DEG were up-regulated (fibroblast growth factors genes, Apc, Nat genes, Ptgs2, Ugt genes, and Mgst genes). After FDR correction, 6 genes involved in fibroblast growth factor, Apc, 1 Nat gene, 1 Ugt gene, and 1 Mgst gene were observed.

Genes related to muscle contraction (Tnnt3, Tnni2, and Mybpc1), transport of molecules (solute carriers, also observed after FDR correction), and haemostasis (Atp1a3, Thbd, and Plau) were also modulated.

Thirty-seven pathways were identified by GSEA (see Supplementary Table 5). Among these pathways, 23 were in common with the ORA and were relevant in the context of the biological functions extracellular matrix organisation, immune response, transport of molecules, metabolism, haemostasis, signalling, and xenobiotics metabolism.

One additional biological function group present after GSEA was the neuronal response with the serotonergic synapse pathway containing genes such as Kcnj6, Htr4 and Itpr3.

To conclude, after 7 days of exposure, the molecular response at the gene expression and pathway levels was larger than 2 days after E171 ingestion in combination of AOM/DSS. The biological response after 7 days of exposure leads towards a deregulation of gene expression levels in the transport of molecules, metabolism, signalling, metabolism of xenobiotics, extracellular matrix, and immune response.

#### 14 days of exposure

After 14 days of exposure to E171, ORA showed significant changes associated with 40 different pathways in 8 biological functions (Table 2, Fig. 2 and Supplementary Table 3): signal transduction, xenobiotics metabolism, metabolism, cancer signalling, digestive system, transport of molecules, and neuronal response. After ORA, 3 of these biological functions were also observed related to the DEG significant after FDR correction: signal transduction, metabolism, and neuronal system.

Genes related to signal transduction were affected after 14 days of exposure and associated to 16 pathways. The DEG in these pathways were 60 to 70% down-regulated (Table 2 and Fig. 2). Among these DEG, some of them are related to development of cancer like B cell lymphoma (Ptger4)^29^ and colorectal cancer (Pyy and Pthlh)^30,31^.

In addition, DEG were affected in 2 other biological functions: xenobiotics metabolism and metabolism. In xenobiotics metabolism, several classes of Cyp450 genes were up-regulated:Cyp2 (with one Cyp2 gene observed after FDR correction), Cyp3, and Cyp5 (see Supplementary Fig. 2). In the metabolism biological function, genes were up-regulated in pathways such as vitamin D metabolism and retinol metabolism. Both pathways are related to inflammation. In both biological functions, most of the genes (90%) were up-regulated (Table 2 and Fig. 2).

Genes involved in cancer and cancer signalling were also modulated after 14 days of exposure to E171. In the pathway chemical carcinogenesis, DEG were mostly up-regulated like Gsto genes (Gsto1 was significant after FDR correction), Ugt genes, and Adh genes.

The outcome of GSEA showed fewer pathways than after ORA, a total of 17 pathways were expressed after 14 days of exposure (see Supplementary Table 5). Among these 17 pathways, the majority (9 pathways) were common to the ORA and associated in several biological functions: signalling, metabolism of protein, metabolism of xenobiotics and digestive system.

Five other pathways were classified in already mentioned biological functions: extracellular matrix organisation, immune system, and haemostasis. The extracellular matrix organisation pathway contained 23 DEG in which 2 were up-regulated, the other genes like matrix metallopeptidase genes, integrin genes, fibulin and fibronectin genes were down-regulated. Within the immune system biological function, the 61 DEG were separated in 3 different pathways related mostly to the innate immune system. A majority of the DEG in these pathways were down-regulated like genes involved in complement, interferons, activation of natural killers genes, whereas the Fas gene was up-regulated. One gene involved in complement was also significant after FDR correction.

To conclude about 14 days of exposure, similar to day 2 and 7, genes were modulated in signalling, cancer signalling, inflammation, immune system and extracellular matrix organisation.

#### 21 days of exposure

After a longer exposure, 21 days, 12 pathways were identified by CPDB after ORA. All details about genes and pathways can be found in Supplementary Table 4. Genes in these pathways were relevant in the context of the signal transduction, immune response, extracellular matrix organisation, and neuronal response.

Forty-eight DEG were related to signal transduction. Ninety percent of these genes were down-regulated (Table 2 and Fig. 2). Some of these genes were also related to development of cancer such as colorectal cancer (Camk2g)^32^, melanoma (Gna11)^33^, breast cancer (Esr1)^34^, and prostate cancer (Akap12)^35^.

The 77 immune system related DEG were down-regulated. These genes were involved in the complement activation, MHC class I and class II presentation, Tlr genes, and cell adhesion molecules genes.

The 23 DEG involved in extracellular matrix organisation were all down-regulated and played a role in integrins, elastase, intercellular adhesion, collagen and actinin.

After GSEA, the number of pathways was higher than after the ORA, 107 pathways classified in already mentioned biological functions (see Supplementary Table 5). Ten out of twelve pathways of the ORA were present in the GSEA and were classified in immune system, neuronal system, extracellular matrix organisation, and signal transduction.

To conclude on 21 days of exposure, both pathway analyses showed an effect of E171 on the immune response, neuronal response, signalling, and extracellular matrix organisation. In addition, the GSEA indicated modulation in cancer signalling pathways, cellular processes, haemostasis, and metabolism of protein. Furthermore, the reaction of the immune system was larger (33 pathways) in the GSEA.

### Comparison of all time points

Some of the effects of E171 on gene expression were in common between the different time points. At all time points, E171 affected mRNA levels related to signalling (olfactory, GPCR, cytokine, cancer signalling) and immune system (innate and adaptive). From 7 to 21 days of exposure genes involved in the extracellular matrix organisation and neuronal system were modulated. After 7 and 14 days of exposure, genes expression levels were significantly different in metabolism, metabolism of xenobiotics, haemostasis, digestive system, and transport of molecules. After FDR correction, genes after 7 and 14 days were commonly involved in signalling, metabolism, neuronal system, and xenobiotics metabolism.

**Figure 3:**
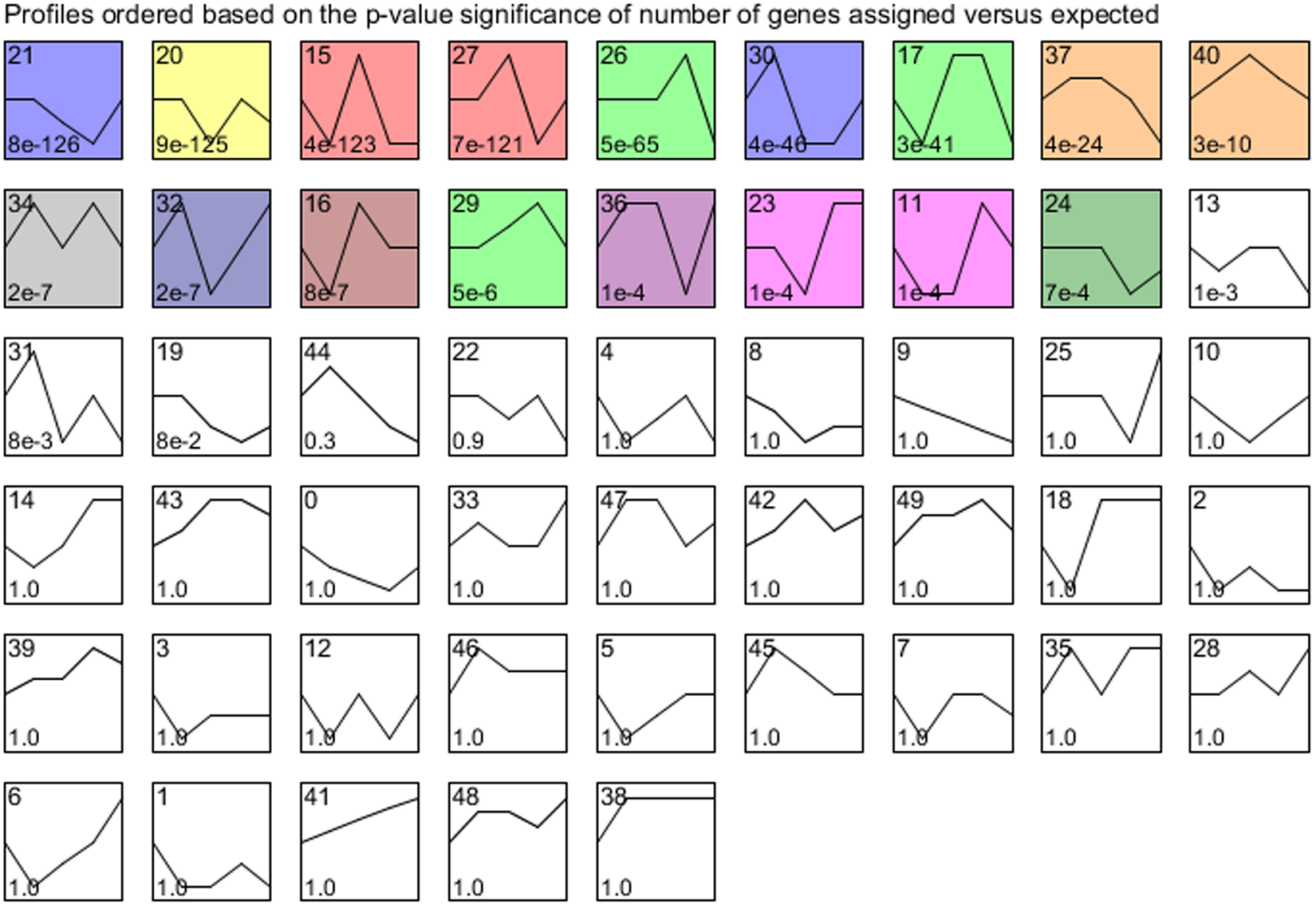
Results of STEM analysis. Analysis performed with all the genes passing the pre-processing. Directionality changes when the maximum unit change in model profiles is between time points is higher than 2. Significant profiles are represented in colour. Similar colours represent the same type of expression profile. The number on the top left corner corresponds to the number of profile. The number at the bottom left corresponds to the associated p-value.

Figure 3, a STEM analysis, shows the directionality of all the genes including the non-differentially expressed ones over time. This analysis indicates, as observed in Table 1 and Fig. 2, that the effects of E171 in a CRC mouse model is driven by the time points 7 and 14d. Within the significant expression profiles, several profiles had a very specific biological process linked to the genes which confirms the effects observed over time. The first profile with a very specific biological response is profile 21, it contains a majority of genes involved in signal transduction pathways (>90%) which is also observed with profile 34. Another profile, profile 15, was specific to immune response genes with over 75% of pathways related to the immune system. The profile is in line with the results observed with the DEG where all the genes were down-regulated after 21d of exposure. The genes involved in cancer were in majority present in profile 40 in which 45% of the related pathways were involved in cancer. All the other significant profiles had several different biological processes involved.

### Time course network analysis

**Figure 4:**
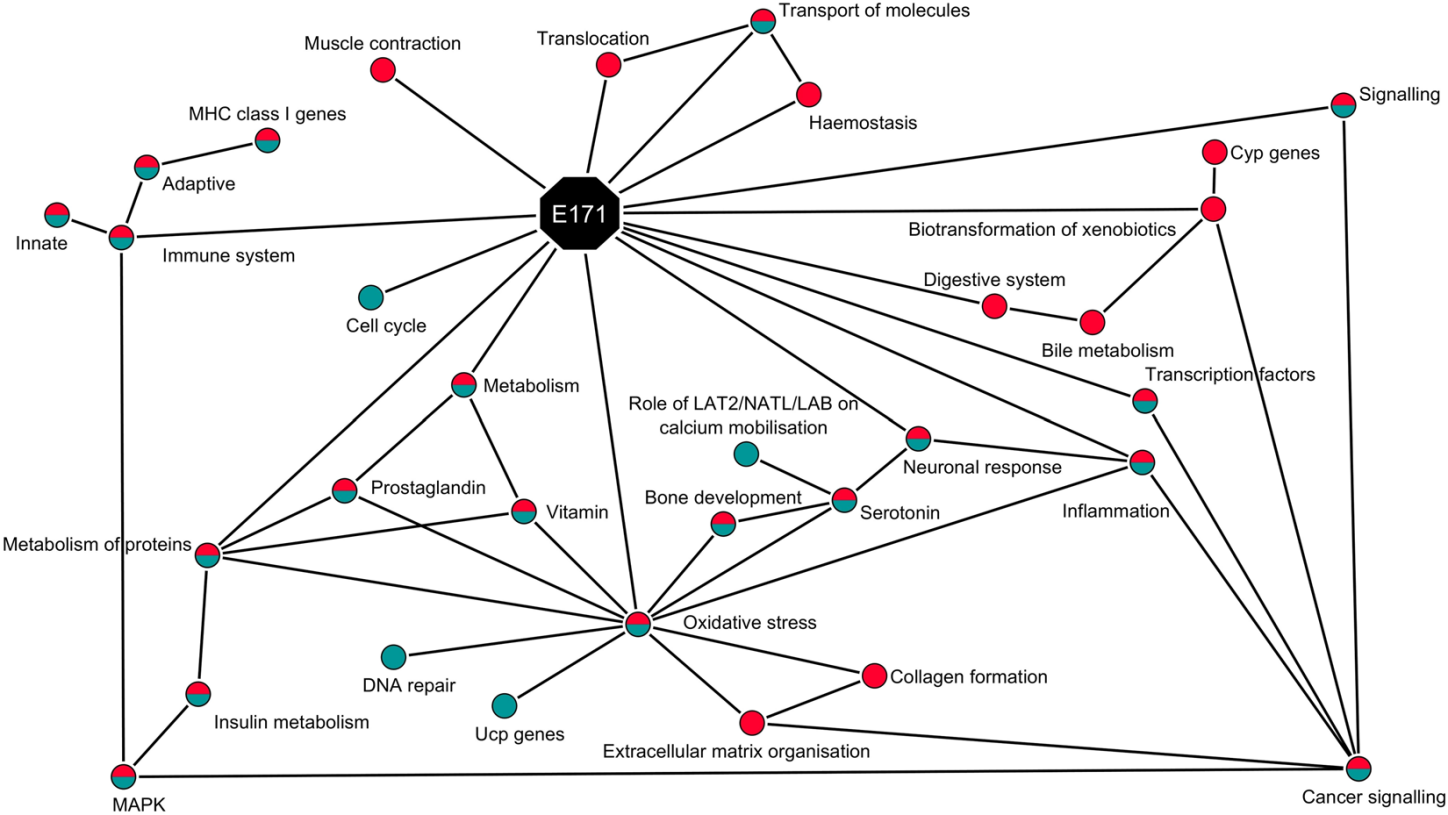
Visualisation the interaction between the different biological processes (circles) regulated after exposure to E171 (octagon) for 2, 7, 14 and 21 days. Network created with Cytoscape. Only seen in the CRC mouse model in combination with E171 are in red, the ones only seen in the mice solely exposed to E171 are in blue^16^, and the ones in common between the previous 2 exposures are in blue and red.

In order to visualise the effects of E171 *in vivo* and compare it to a previous study in which E171 was ingested in BALB/c mice without AOM/DSS treatment^16^, a network was made based on the pathway analyses as well as relevant DEG over time in Fig. 4. This network was created with the groups of pathways and relevant sub-categories (in red). The results were compared to the similar study performed in the same mouse strain exposed only to E171 (in blue) and the common effects observed were shown in blue with a red circle.

After exposure to E171 in the colon of mice in the absence or the presence of AOM/DSS, modulation of genes related to inflammation, oxidative stress, immune reaction, metabolism of protein, and signalling was observed.

In addition to the gene expression seen in both models, other effects have been only observed with the combined exposure to E171 and AOM/DSS. Those effects show modifications related to transcription factors, xenobiotics metabolism, extracellular matrix organisation, transport of molecules, and haemostasis.

## Discussion

In this study, we aim to establish the influence of E171 on gene expression patterns in the colon of a colorectal mouse model after exposure to 5 mg/kg_bw_/day of E171 for 2, 7, 14, and 21 days. These alterations could lay the basis for the development of tumours in colon that we found in a previous study after 4 weeks of exposure^15^. Specifically, the up-regulation of genes involved in inflammation, biotransformation of xenobiotics, genes related to cancer, and impairment of the immune system. Even though the majority of the DEG were not common between time points, their transient nature does not imply that the biological response to E171 is transient as well. In addition, the directionality of a pathway cannot be predicted by the overall interpretation of the directionality of expression of individual genes within this specific pathway. Interpretation of the biological functions of the genes using e.g. GSEA and ORA identified biological processes like immune response, signal transduction, and cancer signalling which were modulated after day 2, 7, 14, and 21. Therefore, the sequence of effects observed may be well in line with the different consecutive mechanisms that contribute to the development of cancer in this model. The mechanisms identified on the DEG and pathways are further elaborated below.

The current study shows that E171 induces molecular changes in translocation, transport of molecules, and muscle contraction. At day 7, genes involved in the muscle contraction pathway were mostly down-regulated. Among these genes, downregulation of Tnnt3, Tnni2, and Mybpc1 is associated with impaired muscle function^36,37^. Presence of E171 may cause a decrease of frequency or strength of muscle contraction in the gut, which would eventually increase absorption and metabolism^38^. Absorption of E171 in the colon of rats has been already observed by Bettini *et al*.^17^. They suggest that accumulation of E171 in the colon is due to the slow transit time and favours local absorption by epithelial cells. Also other groups observed a translocation of TiO_2_ from the gut to other part of the body^39,40^. In addition, changes in haemostasis can influence the absorption of TiO_2_ particles as shown previously^17,39–41^. In our study, a gene related to haemostasis, Atp1a3, was differentially expressed over time (see Supplementary Fig. 1) and codes for an integral membrane protein responsible of establishing and maintaining the electrochemical gradients of Na and K ions across the plasma membrane^42^. In addition, other haemostasis genes were affected after exposure to E171 such as thrombomodulin (Thbd) and plasminogen activator, urokinase (Plau) which are involved in blood coagulation^43,44^.

The ingestion of E171 in the colon also impacts the expression of genes involved in signalling which was a consistent effect across all the different time points (Table 2 and Fig. 2). The GPCR gene family is the largest in the genome and the olfactory receptor gene family is one of its member^45^. These receptors are essential for the signal transduction in cells and are only activated when a compound or element such as a hormone, neurotransmitter, ion, or photon is presented and recognised by the receptors^46,47^. Signal transduction genes were modulated by the exposure to E171 in the colon of mice in presence and also in absence of AOM/DSS^16^. In line with these findings, activation of GPCR/olfactory genes was previously observed after TiO_2_ NPs inhalation in the lung of mice^48^. Repercussions on other gene expression of immune or cancer signalling genes in the cells were observed from 2 to 21 days of exposure. Indeed, increasing evidence linking GPCR/olfactory signalling genes to development of cancer^49,50^ and modulation of the immune system^51,52^ has emerged. In our study, over time, a total of 22 GPCR DEG (in italic in Supplementary Tables 2, 3, and 4) have been identified to be involved in the development of cancer of which 7 genes coding for Wnt signalling, frizzleds and receptors of endothelins, prostaglandins, and thrombin were related to growth, metastasis, survival, angiogenesis, and migration of colorectal cancer^49,50^. Of these 22 genes, 9 were also significant after FDR correction like Wnt2, Fzd4, Apc, Fgf2, Ccl2, and Erbb2. In addition, enhanced expression of 2 genes was also found in the previous study^16^: Esr1 and Ace. Esr1 gene, involved in breast cancer^49,50^, was also down-regulated in both models at day 7 and 14 when exposed to E171 only and at day 7 and 21 in this study. Ace gene was modulated in the opposite directions in both *in vivo* mouse models exposed to E171: up-regulated at day 14 and 21 when exposed to E171 only and down-regulated at day 7 in this study and is involved in the regulation of cell proliferation and angiogenesis^26^.

In our study, an effect on the immune system was observed from 2 to 21 days of exposure with genes involved in the immune response. We noted that these genes were mostly down-regulated after 2 days of exposure and exclusively down-regulated after 21 days (see Supplementary Tables 1–4). This expression profile was also observed over time with the STEM analysis (Fig. 3). These down-regulated genes are involved in the regulation of the innate and adaptive immune system, MHC class I presentation, production of cytokines, natural killer cytotoxicity, and cell adhesion molecules. In order to escape from the innate or the adaptive immune system, tumour cells have the capacity to stop the expression of MHC class I at the surface of the cell and/or activate type 2 macrophages that would express IL-10^HIGH^, IL-12^LOW^ and IL-23^LOW^ an anti-inflammatory phenotype which stimulates cancer proliferation^53–55^.

The results of our study showed that E171 has a stronger effect on the innate than the adaptive immune system. However, the effect observed on the genes not significant after FDR correction involved in the innate and adaptive immune system might facilitate tumour formation. These data are in line with results of other publications on the evasion of tumours, even early tumours to the immune system^53–55^. Furthermore, the downregulation of genes involved in the MHC class I presentation as well as genes of the complement system was also observed in the similar transcriptomics study performed by our group^16^. In addition, Bettini *et al*. also showed that the intragastric exposure to E171 in rats at low doses impairs intestinal immune homeostasis after 1 week of treatment^17^.

As oxidative stress is a factor of development of cancer, a particular focus was on gene directly involved in oxidative stress and genes related to this. Oxidative stress genes were not differentially expressed but some genes indirectly related to oxidative stress like genes involved in antioxidant production were observed. After 7 and 14 days of exposure to E171, metabolism of prostaglandin and vitamin D pathways were affected. After FDR correction, half of the DEG involved in prostaglandin pathways were also differentially expressed. This can be regarded as a potential indication of the presence of oxidative stress. Basu *et al*. showed that a pro-inflammatory environment induced in macrophages, epithelial cells and fibroblasts the production of cyclooxygenase-2 leads to the release of prostaglandins in bone marrow^56^. Activation of vitamin metabolism genes but also oxidative stress genes was also observed after 14 and 21 days of exposure in our previous mouse model exposed to E171^16^.

In addition, several groups observed that the presence of oxidative stress damages the extracellular matrix organisation, decreases the fibrillar collagen synthesis genes, and induces changes in the collagen formation^57–59^. From 7 days, genes involved in the extracellular matrix and collagen were modulated also after FDR correction (Table 2, Fig. 2 and Supplementary Fig. 1). Extracellular matrix genes are also linked to induction of apoptosis in CRC and in gastric cell lines^59,60^. Gencer *et al*. suggest that the activation of Mmp-15 in gastric cancer cell lines is a direct link to oxidative stress. We observed the activation of Mmp-15 after 7 days of exposure to E171 (see Supplementary Table 2). This group also hypothesises that the perturbation of cell matrix adhesion may be a novel mechanism by which a compound can induce apoptosis in colorectal cancer cells.

Oxidative stress also leads to the modulation of serotonin genes^61^. In our study with AOM and DSS, the addition of E171 had also an influence of the mRNA levels of serotonin related genes. Two serotonin genes, Htr7 and Htr1b, were in common between the chemically-induced colorectal cancer mouse model and the previous mouse model exposed to E171 only. They are both serotonin receptor genes and were down-regulated in both models. In this study, other genes in neuronal and serotonin pathways were affected by exposure to E171 at 14 and 21 days (Table 2 and Fig. 2). Alterations in serotonin signalling have been associated with tumour progression in prostate as well as breast cancer^62^, although this has not been shown for colon cancer so far.

By inducing oxidative stress in the colon cells, E171 exposure may results in the production of antioxidants, serotonin, and degradation of extracellular matrix. Indeed, TiO_2_ was previously shown to induce DNA damage and genotoxicity via inflammation and/or oxidative stress in mice as well as *in vitro* in colon cell lines (Caco-2 and HCT116), human epidermal (A431) and HepG2 cells lines^7,16,63–65^. Inflammation by oxidative stress as well as suppressing the immune system can ultimately lead to development of cancer^66^.

The effect of E171 on expression of genes involved in cancer can also be observed from 2 to 14 days of exposure. This expression profile was also observed over time with the STEM analysis (Fig. 3). After 2 days, 6 DEG were related to cancer such as Adcyap1, Ramp1, and Trpc3 which were down-regulated. Adcyap1 methylation is associated with ovarian cancer^21^. In our study, this gene is down-regulated which is in line with the Adcyap1 methylation shown by Jung *et al*. Furthermore, Trpc3 and Ramp1 are associated with ovarian and pancreatic cancer respectively^18,19^. At day 7 and 14, E171 up-regulates genes in cancer signalling pathways such as chemical carcinogenesis and FGFR1/1c/3 and 3c ligand binding. At day 7, these pathways were also observed after ORA on the FDR corrected DEG. In addition, over time, 5 genes were modulated, all significant from 7 days of exposure and all related to development of cancer (see Supplementary Fig. 1). Some DEG were involved specifically in CRC like Itga5, involved in tumour invasion^67^ and Ifitm10 and Sox17, potential markers of CRC^68,69^. Sox17 and Ifitm10 were significantly expressed after FDR correction at day 7 and 14 respectively. The exposure to E171 leads to a modulation of cancer related genes which is in line with our previous study in which mice were exposed to only E171^16^.

Changes in gene expression in the metabolism of xenobiotics is observed from 2 to 21 days and may also contribute to the development of cancer by influencing the metabolism of potential carcinogenic compounds and intermediates. Secondary organs, like intestines, are also a site for biotransformation of compounds^70–72^.

In Supplementary Fig. 2, the modulation of the expression of Cyp450 genes over time is shown to be a consistent effect with a modulation of a total of 43 genes, most of them being differentially expressed at day 7 and 14 (see Supplementary Fig. 2). Of these 43 genes, 21 were also significant after FDR correction. These results are in line with a previous *in vitro* study in which shows that NPs have an impact on with Cyp450 enzymes^73^. Substrates identified for the oxidation in the biotransformation of xenobiotics include saturated and unsaturated fatty acids, sterols, steroids, bile acids, vitamin D3 derivatives and retinoids^74^. After 14 days of exposure, pathways such as vitamin D metabolism, fatty acids, steroid hormones, bile acids (also at day 7) and retinol metabolism were observed (Table 2, Fig. 2 and Supplementary Table 5). The effect of TiO_2_ on steroid hormones was also observed in a previous study. Gao *et al*. showed that after ingestion in mice, TiO_2_ crossed the blood-testis barrier, accumulated in the testes, and affected steroid hormones in serum^39^. After 7 and 14 days of exposure to E171, glutathione (GSH) genes were up- and down-regulated. Two GSH genes, one at day 7 and one at day 14, were also significant after FDR correction. GSH have also been implicated with the potential of forming reactive intermediates resulting in toxic effects^28^. The effects of E171 on the GSH genes is in line with a previous study by Shlukla *et al*. in which they observed that oxidative stress induced by TiO_2_ NPs resulted in a modulation of GSH content in epidermal cells^64^.

The up- and down-regulation of genes involved in the biotransformation of compounds showed a contribution of E171 to the metabolism of various exogenous compounds including drugs, environmental chemicals, pollutants, and natural plant products. The metabolism of xenobiobitics frequently results in successful detoxification of an irritant, but the actions of P450 enzymes may also generate toxic metabolites that contribute to increased risk of cancer, and other toxic effects^28^.

Biotransformation of compounds pathways were not the only type of metabolism pathways affected by E171. At day 7, pathways involved in the metabolism of glucose contained mostly up-regulated genes. After FDR correction, 10 genes were also significant and related to insulin events. These results are in line with previous findings which reported an increase of plasma glucose in mice after exposure to TiO_2_ via the production of ROS that activate the MAPK pathway^40^. In our study, some MAPK genes were also differentially expressed from 7 to 21 days (see Supplementary Table 2, 3 and 4). The activation of the MAPK genes and the metabolism of glucose were also observed after 7 days of exposure when mice were exposed to only E171^16^. Activation of signalling MAPK pathway is important in intestinal epithelial differentiation. Its cascade is involved in control of growth signal, cell survival and invasion in cancer^75^.

Modulations of mRNA levels of transcription factor genes were observed at all time points after E171 exposure. Initiation and regulation of mRNA levels are performed by transcription factors such as Ngfi-A binding protein 2 (Nab2) and cytoplasmic FMR1 interacting protein 1 (Cyfip1). These transcription factors genes were differently expressed from 7 to 21 days. Napoli *et al*. described that Cyfip1 protein binds to the translation initiation factor eIF4E and mediates translational repression in mammalian cells^76^. Cyfip1 upregulation affects general mRNA translation^77^. Cyfip1 was also observed as the only common DEG up-regulated at all time points in a similar study in which mice were exposed to only 5 mg/kg bw/day of E171 for 2, 7, 14 and 21 days^16^. Deregulation of transcription factors leads to deregulation of mRNA levels which can also lead to development of cancer with transcription factors genes like Cyfip1^78^ and Nab2^79^.

In summary, in this study we demonstrated that gene expression in the colon of mice is affected by exposure to E171 in combination with AOM/DSS. Over time, GPCR/olfactory signalling genes were modulated including genes involved in cancer and particularly colorectal cancer. Furthermore, the modulation of gene expression levels after E171 exposure on the biotransformation of xenobiotics shows a detoxification which increases the metabolism of potential carcinogenic compounds and intermediates. In addition, E171 modulated gene expression of immune related genes with a majority of the innate and adaptive immune system genes down-regulated. These results suggest an impairment of the immune system which can ultimately facilitate the development of cancer. E171 ingestion also induced changes at the mRNA levels of vitamin and prostaglandin genes as well as extracellular matrix organisation, collagen formation, and activation of MAPK genes which suggest the presence of oxidative stress responses. With a lower number of genes, these effects were also observed after FDR correction at day 7 and 14. Presence of oxidative stress, impairment of immune system, and modulation of cancer related genes are effects in line with the tumour formation observed previously by our group^15^ and in line with the transcriptomics study after exposure to E171 only^16^. The presence of AOM/DSS compared to the absence of these compounds in our previous study shows that AOM/DSS enhanced all observed biological reactions affected by the exposure to E171.

Altogether, the results of our animal studies and those of others warrant further investigation of the potential adverse effects of consumption of food additive E171 in human.

## Materials and Methods

### E171 particle characterisation

E171 was generously donated by the Sensient Technologies Company in Mexico. Characterisation of E171 was performed previously by electron microscopy with Scios DualBeam FIB/SEM (SEM, 20 KV, the Netherlands) at 150,000x magnification to evaluate the size and morphology of the particles^6^. E171 contains slightly to fully rounded particles with a proportion of 39% NPs and 61% MPs.

### Mouse model

**Figure 5:**
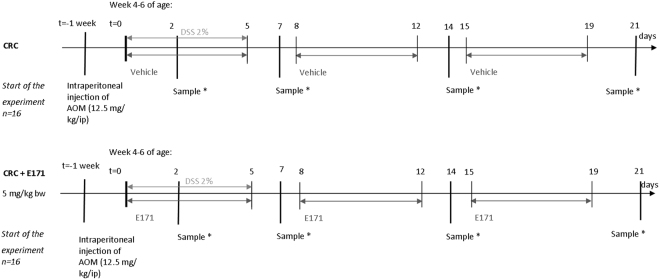
Scheme of exposure of experimental mouse model. BALB/c mice (n = 32) were randomly distributed in 2 groups (16 per group) and were kept one week under acclimation conditions. Both the control and exposure group received a single intraperitoneal injection 12.5 mg/kg of AOM a week before the start of the experiment and DSS 2% dissolved in water *ad libitum* between day 1 and 5 respectively (light grey line). From day 1 to 21, E171 group received 5 mg/kg body weight by oral gavage of E171 dispersed in water 5 days per week (black line). The control group kept on receiving a vehicle (sterile water). *2 males and 2 females were sampled.

Exposure of BALB/c mice to E171 by ingestion was performed at the Unidad de Biomedicina, Facultad de Estudios Superiores Iztacala, Universidad Nacional Autónoma de México, Mexico. Ethical approval was given by the Comité de Ética de la Facultad de Estudios Superiores Iztacala de la Universidad Nacional Autónoma de México under the number: FESI-ICY-I151. Guidelines of Norma Oficial Mexicana (NOM-062-ZOO-1999, NOM-087-ECOL-1995) were followed as well as the Protocol for the Care and Use of Laboratory Animals (PICUAL). The exposure of BALB/c mice was performed at the same time as a similar experiment^16^ with a few changes as described below. After one week of acclimatisation, 32 BALB/c mice (16 males, 16 females) of 4–6 weeks of age (Harlan Laboratories, Mexico) were randomly placed into 2 groups: a) control group (AOM/DSS) (8 males, 8 females) named CRC and b) E171 group with AOM/DSS (8 males, 8 females) named CRC + E171(Fig. 5). One week before the start of the experiment, both groups received a single intraperitoneal injection of 12.5 mg/kg_bw_ of AOM (Sigma, USA) as described in the chemically colitis-associated colorectal cancer mouse model of Tanaka *et al*.^80^. The first 5 days of the experiment, both groups were given 2% DSS (MW 35000–50000, MP Biomedicals, Solon OH, USA) dissolved in water and filtered *ad libitum*. In addition, the E171 group received an intragastric administration of 5 mg/kg_bw_ of E171 dispersed in drinking water and sonicated 30 min at 60 Hz by a gavage feeding 5 days per week for 21 days according to the scheme (Fig. 5). The control group received 100 µL of sterile sonicated water (30 min at 60 Hz) by intragastric gavage 5 days per week during 21 days according to the scheme.

### mRNA isolation from colonic tissues

mRNA extraction from the distal colons followed the same protocol as the previous study^16^. In short, mRNA was isolated according to the manufacturer’s protocols for “Animal Cells and Animal Tissues”^81^, the miRNeasy Mini Kit (Qiagen, The Netherlands) with DNase treatment (Qiagen, The Netherlands). Total RNA yield as well as the 260/230 and the 260/280 were measured on a Nanodrop® ND-1000 spectrophotometer (Thermo Fischer). Samples with a 260/230 ratio between 1.8 and 2.0 and a 260/280 ratio between 1.9 and 2.1 were checked for their integrity. The integrity of total RNA was checked using RNA Nanochips on a 2100 Bioanalyzer using manufacturer’s protocol (Agilent Technologies, The Netherlands). All samples had an RNA integrity number (RIN) above 6, the number at which the sample is approved for microarray analysis. The average of all was 8.8 ± 0.7.

### cRNA synthesis, labelling and hybridization

Preparation of the samples for microarray analysis was also performed with the exact same conditions as previously^16^. In short, according to the One-Color Microarray-Based Gene Expression Analysis protocol version 6.6^82^, 200 ng of total RNA was synthesized into cRNA and labelled. The procedure was performed as described by the manufacturer’s protocol. Purification of the amplified cRNA samples was performed with the RNeasy Mini Kit (Qiagen, The Netherlands) according to the manufacturer’s protocol of Agilent. cRNA samples were quantified using a Nanodrop® ND-1000 spectrophotometer with a Microarray Measurement.

A quantity of 600 ng of labelled cRNA was hybridized on the microarray slide. Hybridization was performed according to the Agilent’s protocol on SurePrint G3 mouse Gene exp 60 kv2 microarrays slides (Agilent Technologies, The Netherlands). Furthermore microarray slides were scanned using an Agilent DNA Microarray Scanner with Surescan High-resolution Technology (Agilent Technologies, The Netherlands).

### Pre-processing and data analysis of microarrays

Pre-processing methods were described previously^16^. In short, the quality of the microarrays was first verified by the quality control pipeline provided by Agilent (Feature extraction software (FES) version 10.7.3.1). All samples met the quality criteria of the FES. All samples met the in-house quality check pipeline previously published (github.com/BiGCAT-UM/arrayQC_Module). Raw data with both expression values and genes were selected for data analysis based on flags and missing values. Eight groups were defined: E171 + AOM/DSS (CRC + E171) 2 days, 7 days, 14 days, and 21 days for the exposed samples and control AOM/DSS (CRC) 2 days, 7 days, 14 days, and 21 days for the controls. Within each group, spot identifiers were removed if flagged by the in-house quality control. Spot identifiers were deleted when more than 40% of samples in each group have a missing value and when the average expression was less than four in all groups. Pre-processing of missing values was performed using the standard settings of the GenePattern ImputeMissingValues.KNN module v13^83^. Spot identifiers were annotated to Agilent probe identifiers and merged with Babelomics 5^84^. Next, Agilent probe identifiers were re-annotated to EntrezGeneIDs and merged with Babelomics 5. By using LIMMA (version 1.0), data of the exposed samples (CRC + E171) were compared to their time matched control sample (CRC) and DEG were identified. The standard cut-off values of a fold-change (Log2FC) of 1.5 and a p-value of 0.05 were used^85^. In addition, the false discovery rate was calculated according to the Benjamini-Hochberg method with a threshold at 0.05.

### Pathway analyses

ORA was performed in CPDB with the DEG of each time point^86^. For each annotation set, the p-value was calculated. Within each type of annotation set CPBD corrects for multiple hypothesis testing using the false discovery rate procedure^86,87^. All available databases from CPDB were used (release 31, 1 sept. 2015) with settings in the “pathways as defined by pathway databases” with a minimum overlap of input list of 2 and a p-value cut-off of p < 0.01.

With regard to the pathway analyses applied in the previous study, as second pathway analysis, GSEA, was performed. Like the ORA, the GSEA was performed using CPDB. All the available databases from CPDB were used with settings in the “pathways as defined by pathway databases” with a minimum number of measured genes of 4 and a p-value cut-off of p < 0.01.

### Pathway construction

Biotransformation of compound pathway Phase I and II *Mus musculus* (WP702(r29945)) was downloaded from the WikiPathway database (August 2016) as an analogue of the *Homo sapiens* pathway with a 64% conversion rate^88^ and uploaded on the Pathvisio software (version 3.2.4)^89^. The gene list of this pathway was adapted to the DEG present in the dataset across all time points. Expression data from the DEG of all time points was imported and coloured according to the Log2FC values obtained after LIMMA. Genes coloured in blue are down-regulated and genes coloured in red are up-regulated.

### Network construction

Summary network of the effects of E171 and its comparison to a previous mouse model in which mice were exposed to E171 only^16^ was performed with Cytoscape (version 3.4)^90^. All nodes and edges were added according to the biological effects observed at the pathway and gene levels. The biological processes only seen in the CRC mouse model in combination with E171 are in red, the ones only seen in the mice solely exposed to E171 are in blue. Common biological effects with the previous mouse model were coloured red and blue.

### STEM analysis

STEM analysis was performed with the tool Short Time-series Expression Miner developed by Ernst and Bar-Joseph to analyse the expression pattern over time of short time series, below 8 time points^91^.

For the analysis, all genes passing the pre-processing were used which includes the non-differentially expressed genes. The gene annotation source was the mouse (EBI), no cross reference, no gene location, and no normalization were used. The clustering method was the STEM clustering method with a maximum of 50 models of profiles. Each expression of a gene is compared to the previous time point and the maximum unit change in model profiles between time points was 2. Significance is calculated by comparing actual number of genes per cluster to expected number of genes.

### Data availability

The datasets generated and analysed during the current study are available in the Gene Expression Omnibus (GEO) repository (GEO accession: GSE109520), https://www.ncbi.nlm.nih.gov/geo/.

## Electronic supplementary material

Supplementary materials
