# Supplementary material for: Transcriptomics analysis reveals new insights in E171-induced molecular alterations in a mouse model of colon cancer
Source: Sci Rep. 2018 Jun 27;8:9738. doi: 10.1038/s41598-018-28063-z (PMC6021444; doi:10.1038/s41598-018-28063-z)
Supplement: Supplementary file 1 — Supplementary materials [file 41598_2018_28063_MOESM1_ESM.pdf]

Supplementary information

Transcriptomics analysis reveals new insights in E171-induced molecular alterations in a mouse model of colon cancer

Héloïse Proquin[1@], Marlon J. Jetten[1], Marloes Jonkhout[1], Luis Guillermo Garduño-Balderas[2], Jacob J. Briedé[1], Theo M. de Kok[1], Henk van Loveren[1], Yolanda I. Chirino[2,3]

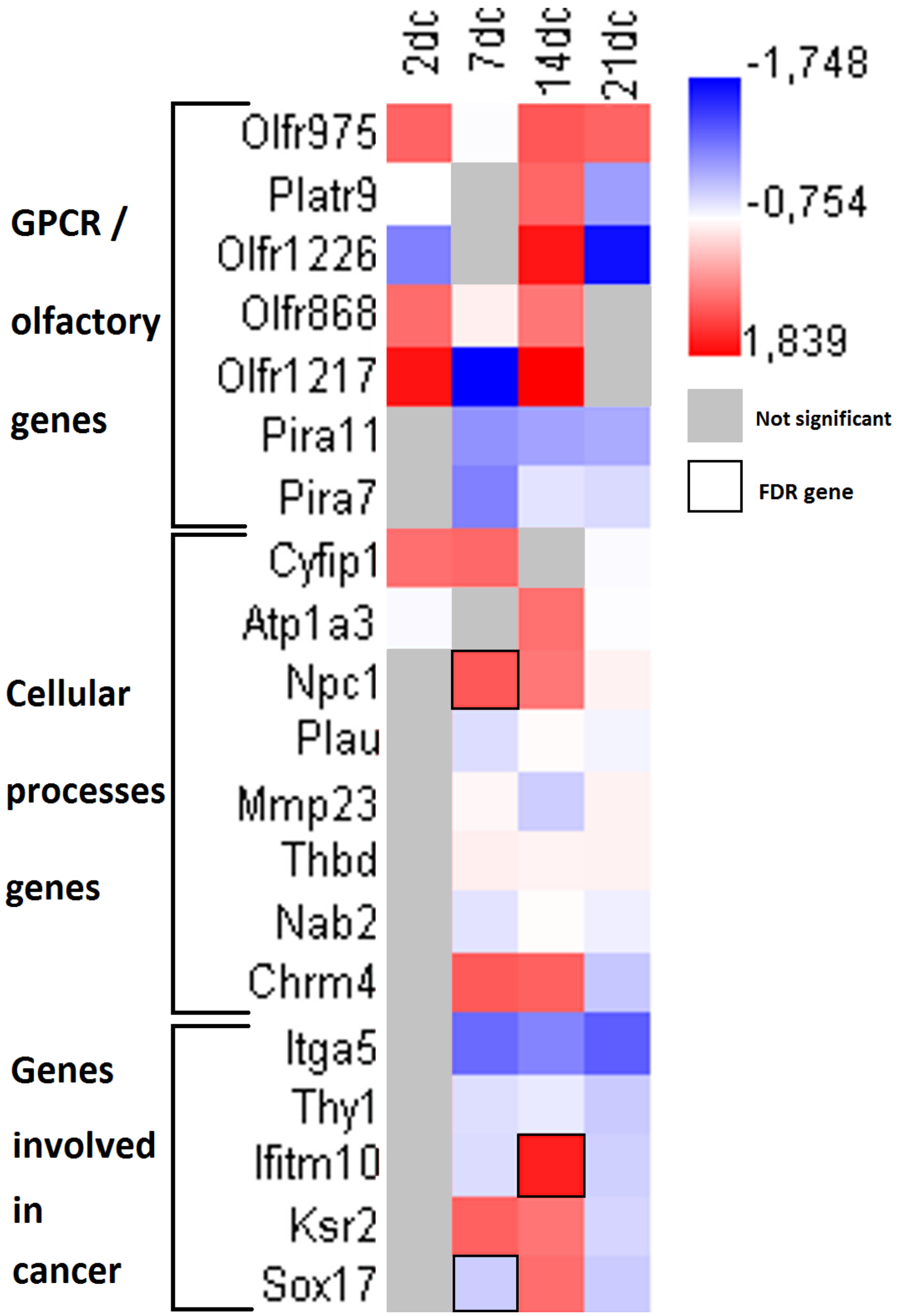

Supplementary Figure 1: Heat map of DEG (p<0.05 and Log2FC>1.5) common in 3 out of 4 time points or in all time points after exposure to E171 in colon of mice for 2, 7, 14 and 21 days.

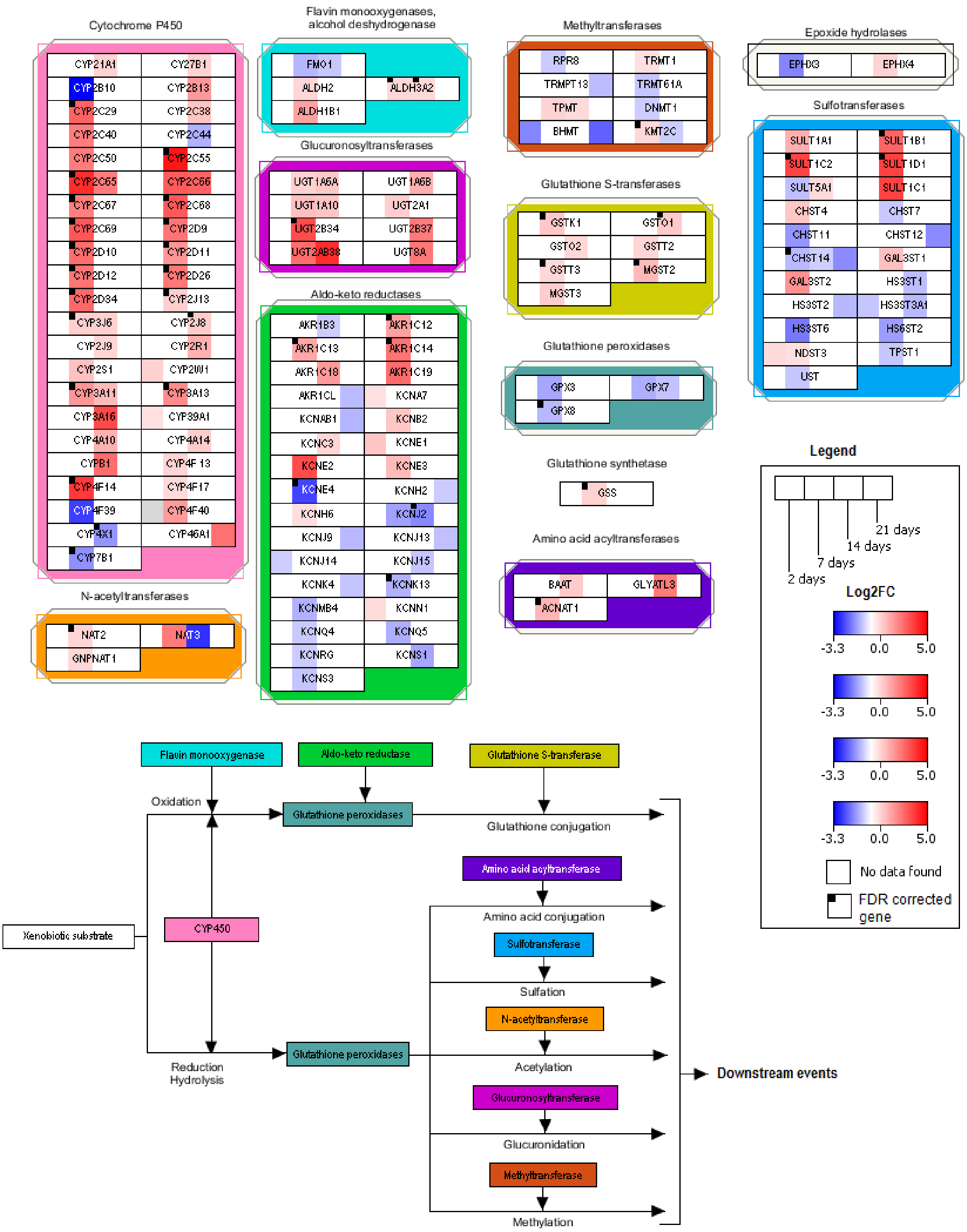

Supplementary Figure 2: Metapathway of biotransformation Phase I and II with DEGs after exposure to E171 for 2, 7, 14 and 21 days in the colon of mice.

Supplementary Table 1: Details results of ORA after 2 days of exposure of E171 in BALB/c mice in combination with AOM/DSS. Numbers in bold correspond to upregulated genes. Log2FC= Log2 fold change obtained with LIMMA script with correction for its own time-matched control.

Group of pathways	Pathways	EntrezGeneID	Genes	Log2FC
Signalling	Olfactory transduction	258542	Olfr786	-3.17822
		384732	Gm10081	-1.50511
		258882	Olfr874	-1.49093
		258969	Olfr1226	-1.24509
		257910	Olfr671	-0.66153
		**258272**	**Olfr1402**	**0.697679**
		**57269**	**Olfr1507**	**0.72437**
		**258552**	**Olfr868**	**0.737959**
		**258328**	**Olfr954**	**0.753012**
		**258581**	**Olfr1030**	**0.771431**
		**259119**	**Olfr578**	**0.793142**
		**258825**	**Olfr975**	**0.82989**
		**258487**	**Olfr722**	**0.834**
		**258436**	**Olfr458**	**0.849033**
		**404311**	**Olfr209**	**0.879874**
		**404307**	**Olfr100**	**0.894926**
		**257932**	**Olfr332**	**0.911724**
		**258730**	**Olfr483**	**0.959872**
		**258938**	**Olfr1417**	**0.998041**
		**258269**	**Olfr930**	**1.062573**
		**258379**	**Olfr1284**	**1.119586**
		**258823**	**Olfr969**	**1.169048**
		**258705**	**Olfr398**	**1.220772**
		**258623**	**Olfr123**	**1.239045**
		**258281**	**Olfr780**	**1.274172**
		**57251**	**Olfr870**	**1.361035**
		**258616**	**Olfr357**	**1.496832**
		**258903**	**Olfr1217**	**1.653872**
		**18332**	**Olfr33**	**1.754651**
		**258065**	**Olfr312**	**1.769029**
		**404315**	**Olfr372**	**2.603612**
	Olfactory signalling Pathway	258542	Olfr786	-3.17822
		384732	Gm10081	-1.50511
		258882	Olfr874	-1.49093
		258969	Olfr1226	-1.24509

		**257910**	**Olfr671**	**-0.66153**
		**258064**	**Olfr316**	**0.63679**
		**258552**	**Olfr868**	**0.737959**
		**259119**	**Olfr578**	**0.793142**
		**258487**	**Olfr722**	**0.834**
		**258730**	**Olfr483**	**0.959872**
		**258269**	**Olfr930**	**1.062573**
		**258281**	**Olfr780**	**1.274172**
		**258616**	**Olfr357**	**1.496832**
		**258903**	**Olfr1217**	**1.653872**
		**404315**	**Olfr372**	**2.603612**
	GPCR downstream signalling	258542	Olfr786	-3.17822
		384732	Gm10081	-1.50511
		258882	Olfr874	-1.49093
		11516	Adcyap1	-1.36922
		51801	Ramp1	-1.36477
		258969	Olfr1226	-1.24509
		257910	Olfr671	-0.66153
		22065	Trpc3	-0.61276
		**18798**	**Plcb4**	**0.621196**
		**258064**	**Olfr316**	**0.63679**
		**258552**	**Olfr868**	**0.737959**
		**19218**	**Ptger3**	**0.76914**
		**259119**	**Olfr578**	**0.793142**
		**258487**	**Olfr722**	**0.834**
		**12426**	**Cckbr**	**0.939752**
		**258730**	**Olfr483**	**0.959872**
		**258269**	**Olfr930**	**1.062573**
		**258281**	**Olfr780**	**1.274172**
		**258616**	**Olfr357**	**1.496832**
		**258903**	**Olfr1217**	**1.653872**
		**404315**	**Olfr372**	**2.603612**
	Signalling by GPCR	258542	Olfr786	-3.17822
		384732	Gm10081	-1.50511
		258882	Olfr874	-1.49093
		11516	Adcyap1	-1.36922
		51801	Ramp1	-1.36477
		258969	Olfr1226	-1.24509
		257910	Olfr671	-0.66153
		22065	Trpc3	-0.61276
		**18798**	**Plcb4**	**0.621196**

		**258064**	**Olfr316**	**0.63679**
		**258552**	**Olfr868**	**0.737959**
		**19218**	**Ptger3**	**0.76914**
		**259119**	**Olfr578**	**0.793142**
		**258487**	**Olfr722**	**0.834**
		**12426**	**Cckbr**	**0.939752**
		**258730**	**Olfr483**	**0.959872**
		**258269**	**Olfr930**	**1.062573**
		**258281**	**Olfr780**	**1.274172**
		**258616**	**Olfr357**	**1.496832**
		**258903**	**Olfr1217**	**1.653872**
		**83770**	**Tas1r2**	**1.725032**
		**404315**	**Olfr372**	**2.603612**

Supplementary Table 2: Details results of ORA after 7 days of exposure of E171 in BALB/c mice in combination with AOM/DSS. Numbers in bold correspond to upregulated genes. Log2FC= Log2 fold change obtained with LIMMA script with correction for its own time-matched control.

Group of pathways	Pathways	EntrezGeneID	Genes	Log2FC
Signalling	Class A/1 (Rhodopsin-like receptors)	*20311*	*Cxcl5*	*-4.34495*
		14825	Cxcl1	-3.66321
		12765	*Cxcr2*	*-3.17533*
		14293	Fpr1	-3.03344
		381489	Rxfp1	-2.58548
		14289	Fpr2	-2.50436
		20306	Ccl7	-2.30947
		109648	Npy	-2.19308
		11835	*Ar*	*-1.51719*
		30878	Apln	-2.06475
		16995	Ltb4r1	-1.87125
		11541	Adora2b	-1.82001
		20303	Ccl4	-1.80734
		12273	C5ar1	-1.78012
		18610	Pdyn	-1.69401
		80885	Hcar2	-1.60399
		12778	Ackr3	-1.56997
		54140	Avpr1a	-1.40933
		19220	Ptgfr	-1.35934
		22044	Trh	-1.26849
		19218	Ptger3	-1.17597
		21336	Tacr1	-1.16015
		11542	Adora3	-1.12328
		59289	Ackr2	-1.106
		12774	Ccr5	-1.06964
		19222	Ptgir	-1.06711
		56744	Pf4	-1.01392
		14764	Ptgdr2	-0.99233
		78134	Lpar4	-0.98779
		14539	Opn1mw	-0.9569
		12062	*Bdkrb2*	*-0.94312*
		321020	Fpr-rs6	-0.93244
		20308	Ccl9	-0.9276
		13617	Ednra	-0.86901
		11555	Adrb2	-0.84979

		11540	Adora2a	-0.8378
		54199	Ccrl2	-0.76284
		319197	Gpr4	-0.74029
		68039	*Nmb*	*-0.72562*
		19204	Ptafr	-0.72302
		14739	S1pr2	-0.68506
		12267	C3ar1	-0.68227
		19217	*Ptger2*	*-0.65162*
		1E+08	Gm20878	-0.60711
		12061	Bdkrb1	-0.60327
		**12672**	**Chrm4**	**0.91934**
		**12802**	**Cnr2**	**0.93971**
		280287	***Kiss1***	***0.944924***
		**20208**	**Saa1**	**0.949254**
		**15562**	**Htr4**	**0.991937**
		**20605**	**Sstr1**	**1.048219**
		**13051**	**Cx3cr1**	**1.128915**
		**107221**	**Ffar4**	**1.192343**
		**21337**	**Tacr2**	**1.265931**
		**12000**	**Avpr2**	**1.346946**
		**14063**	**F2rl1**	**1.373368**
		**16963**	**Xcl1**	**1.565947**
		**18387**	**Oprk1**	**1.723827**
		**56838**	**Ccl28**	**1.79597**
		**216749**	**Nmur2**	**1.868133**
		**16867**	**Lhcgr**	**1.871822**
		**20209**	**Saa2**	**1.96164**
		**14829**	**Grpr**	**2.004654**
		**574402**	**Gpr17**	**2.109197**
		**233079**	**Ffar2**	**2.134441**
		**13615**	**Edn2**	**2.907491**
	GPCR ligand binding	*20311*	*Cxcl5*	*-4.34495*
		14825	Cxcl1	-3.66321
		12765	*Cxcr2*	*-3.17533*
		14293	Fpr1	-3.03344
		12310	Calca	-2.88368
		381489	Rxfp1	-2.58548
		14289	Fpr2	-2.50436
		20306	Ccl7	-2.30947
		109648	Npy	-2.19308
		30878	Apln	-2.06475

		16995	Ltb4r1	-1.87125
		11541	Adora2b	-1.82001
		20303	Ccl4	-1.80734
		12273	C5ar1	-1.78012
		18610	Pdyn	-1.69401
		80885	Hcar2	-1.60399
		12778	Ackr3	-1.56997
		11535	Adm	-1.47588
		54140	Avpr1a	-1.40933
		19220	Ptgfr	-1.35934
		22044	Trh	-1.26849
		19218	Ptger3	-1.17597
		21336	Tacr1	-1.16015
		574417	Tas2r137	-1.1489
		19227	Pthlh	-1.14587
		11542	Adora3	-1.12328
		56089	Ramp3	-1.11478
		59289	Ackr2	-1.106
		12774	Ccr5	-1.06964
		19222	Ptgir	-1.06711
		56744	Pf4	-1.01392
		14764	Ptgdr2	-0.99233
		78134	Lpar4	-0.98779
		14539	Opn1mw	-0.9569
		12062	*Bdkrb2*	*-0.94312*
		321020	Fpr-rs6	-0.93244
		20308	Ccl9	-0.9276
		14366	*Fzd4*	*-0.87772*
		13617	Ednra	-0.86901
		11555	Adrb2	-0.84979
		11540	Adora2a	-0.8378
		12922	Crhr2	-0.83428
		54409	Ramp2	-0.79815
		54199	Ccrl2	-0.76284
		319197	Gpr4	-0.74029
		14369	*Fzd7*	*-0.735*
		68039	*Nmb*	*-0.72562*
		19204	Ptafr	-0.72302
		14709	Gng8	-0.72016
		14739	S1pr2	-0.68506
		12267	C3ar1	-0.68227

		19217	*Ptger2*	*-0.65162*
		14708	Gng7	-0.64044
		19228	Pth1r	-0.61805
		1E+08	Gm20878	-0.60711
		12061	Bdkrb1	-0.60327
		**12672**	**Chrm4**	**0.91934**
		**12802**	**Cnr2**	**0.93971**
		280287	***Kiss1***	***0.944924***
		**20208**	**Saa1**	**0.949254**
		**15562**	**Htr4**	**0.991937**
		**20605**	**Sstr1**	**1.048219**
		**13051**	**Cx3cr1**	**1.128915**
		**107221**	**Ffar4**	**1.192343**
		**21337**	**Tacr2**	**1.265931**
		**14367**	**Fzd5**	**1.338752**
		**12000**	**Avpr2**	**1.346946**
		**14063**	**F2rl1**	**1.373368**
		**16963**	**Xcl1**	**1.565947**
		**18387**	**Oprk1**	**1.723827**
		**56838**	**Ccl28**	**1.79597**
		**216749**	**Nmur2**	**1.868133**
		**16867**	**Lhcgr**	**1.871822**
		**20287**	**Sct**	**1.903349**
		**20209**	**Saa2**	**1.96164**
		**14829**	**Grpr**	**2.004654**
		**574402**	**Gpr17**	**2.109197**
		**233079**	**Ffar2**	**2.134441**
		**13615**	**Edn2**	**2.907491**
	Peptide ligand-binding receptors	*20311*	*Cxcl5*	*-4.34495*
		14825	Cxcl1	-3.66321
		12765	*Cxcr2*	*-3.17533*
		14293	Fpr1	-3.03344
		381489	Rxfp1	-2.58548
		14289	Fpr2	-2.50436
		20306	Ccl7	-2.30947
		109648	Npy	-2.19308
		30878	Apln	-2.06475
		20303	Ccl4	-1.80734
		12273	C5ar1	-1.78012
		18610	Pdyn	-1.69401
		12778	Ackr3	-1.56997

		54140	Avpr1a	-1.40933
		22044	Trh	-1.26849
		21336	Tacr1	-1.16015
		59289	Ackr2	-1.106
		12774	Ccr5	-1.06964
		56744	Pf4	-1.01392
		12062	*Bdkrb2*	*-0.94312*
		321020	Fpr-rs6	-0.93244
		20308	Ccl9	-0.9276
		13617	Ednra	-0.86901
		54199	Ccrl2	-0.76284
		68039	*Nmb*	*-0.72562*
		12267	C3ar1	-0.68227
		100041579	Gm20878	-0.60711
		12061	Bdkrb1	-0.60327
		280287	***Kiss1***	***0.944924***
		**20208**	**Saa1**	**0.949254**
		**20605**	**Sstr1**	**1.048219**
		**13051**	**Cx3cr1**	**1.128915**
		**21337**	**Tacr2**	**1.265931**
		**12000**	**Avpr2**	**1.346946**
		**14063**	**F2rl1**	**1.373368**
		**16963**	**Xcl1**	**1.565947**
		**18387**	**Oprk1**	**1.723827**
		**56838**	**Ccl28**	**1.79597**
		**216749**	**Nmur2**	**1.868133**
		**20209**	**Saa2**	**1.96164**
		**14829**	**Grpr**	**2.004654**
		**13615**	**Edn2**	**2.907491**
	G alpha (s) signalling events	12310	Calca	-2.88368
		381489	Rxfp1	-2.58548
		11541	Adora2b	-1.82001
		11535	Adm	-1.47588
		18578	Pde4b	-1.32826
		23984	Pde10a	-1.18192
		104110	Adcy4	-1.16978
		19227	Pthlh	-1.14587
		56089	Ramp3	-1.11478
		19222	Ptgir	-1.06711
		210044	Adcy2	-0.99756
		11555	Adrb2	-0.84979

		11540	Adora2a	-0.8378
		12922	Crhr2	-0.83428
		54409	Ramp2	-0.79815
		19217	*Ptger2*	*-0.65162*
		18577	Pde4a	-0.62155
		19228	Pth1r	-0.61805
		**54611**	**Pde3a**	**0.664187**
		**207728**	**Pde2a**	**0.669864**
		**18583**	**Pde7a**	**0.848634**
		**11512**	**Adcy6**	**0.967657**
		**15562**	**Htr4**	**0.991937**
		**22354**	**Vipr1**	**0.99492**
		**12000**	**Avpr2**	**1.346946**
		**16867**	**Lhcgr**	**1.871822**
		**20287**	**Sct**	**1.903349**
	Non-odorant GPCRs	*12765*	*Cxcr2*	*-3.17533*
		14293	Fpr1	-3.03344
		381489	Rxfp1	-2.58548
		16995	Ltb4r1	-1.87125
		11541	Adora2b	-1.82001
		12273	C5ar1	-1.78012
		80885	Hcar2	-1.60399
		12778	Ackr3	-1.56997
		13349	Ackr1	-1.56424
		64297	Gprc5b	-1.45985
		54140	Avpr1a	-1.40933
		19220	Ptgfr	-1.35934
		12772	Ccr2	-1.18817
		19218	Ptger3	-1.17597
		21336	Tacr1	-1.16015
		574417	Tas2r137	-1.1489
		11542	Adora3	-1.12328
		59289	Ackr2	-1.106
		19222	Ptgir	-1.06711
		78134	Lpar4	-0.98779
		12776	Ccr8	-0.96612
		12062	*Bdkrb2*	*-0.94312*
		14366	*Fzd4*	*-0.87772*
		13617	Ednra	-0.86901
		14368	Fzd6	-0.86552
		11555	Adrb2	-0.84979

		11540	Adora2a	-0.8378
		12922	Crhr2	-0.83428
		54199	Ccrl2	-0.76284
		14369	*Fzd7*	*-0.735*
		19204	Ptafr	-0.72302
		14739	S1pr2	-0.68506
		12267	C3ar1	-0.68227
		19217	*Ptger2*	*-0.65162*
		19228	Pth1r	-0.61805
		12061	Bdkrb1	-0.60327
		**12672**	**Chrm4**	**0.91934**
		**12802**	**Cnr2**	**0.93971**
		**15562**	**Htr4**	**0.991937**
		**22354**	**Vipr1**	**0.99492**
		**20605**	**Sstr1**	**1.048219**
		**13051**	**Cx3cr1**	**1.128915**
		**107221**	**Ffar4**	**1.192343**
		**236781**	**Gpr119**	**1.210129**
		**21337**	**Tacr2**	**1.265931**
		**14367**	**Fzd5**	**1.338752**
		**12000**	**Avpr2**	**1.346946**
		**14063**	**F2rl1**	**1.373368**
		**18387**	**Oprk1**	**1.723827**
		**216749**	**Nmur2**	**1.868133**
		**16867**	**Lhcgr**	**1.871822**
		**14829**	**Grpr**	**2.004654**
		**574402**	**Gpr17**	**2.109197**
		**23832**	**Xcr1**	**2.111892**
		**233079**	**Ffar2**	**2.134441**
	Antagonism of Activin by Follistatin	16324	Inhbb	-2.62677
		16323	Inhba	-2.3647
		14313	Fst	-1.657402
		83554	Fstl3	-1.079992
Xenobiotics metabolism	Phase 1 - Functionalization of compounds	13088	Cyp2b10	-3.70625
		13123	Cyp7b1	-1.44706
		19223	Ptgis	-1.18808
		14261	Fmo1	-0.93541
		228608	Smox	-0.64588
		212503	Paox	-0.60891
		11669	Aldh2	0.818158
		230459	Cyp2j13	0.92393

		13110	Cyp2j6	0.955546
		60525	Acss2	1.046677
		68738	Acss1	1.103326
		74134	Cyp2s1	1.216227
		631304	Cyp4f40	1.947573
		13112	Cyp3a11	2.066924
		545123	Cyp2d11	2.074292
		17161	Maoa	2.081097
		13113	Cyp3a13	2.238127
		545288	Cyp2c67	2.508173
		13099	Cyp2c40	2.536402
		107141	Cyp2c50	2.555901
		76279	Cyp2d26	2.666213
		380997	Cyp2d12	2.687051
		13105	Cyp2d9	2.696041
		13101	Cyp2d10	2.835051
		13095	Cyp2c29	3.072696
		69888	Cyp2c66	3.206495
		1E+08	Cyp2c69	3.276957
		64385	Cyp4f14	3.873506
		72303	Cyp2c65	4.136602
		72082	Cyp2c55	4.559563
	Biological oxidations	13088	Cyp2b10	-3.70625
		117147	Acsm1	-1.45708
		13123	Cyp7b1	-1.44706
		19223	Ptgis	-1.18808
		110175	Ggct	-1.00265
		14261	Fmo1	-0.93541
		228608	Smox	-0.64588
		212503	Paox	-0.60891
		**103140**	**Gstt3**	**0.777197**
		**14872**	**Gstt2**	**0.813855**
		**11669**	**Aldh2**	**0.818158**
		**17961**	**Nat2**	**0.824478**
		**22017**	**Tpmt**	**0.854172**
		**14854**	**Gss**	**0.871117**
		**94284**	**Ugt1a6a**	**0.900385**
		**22235**	**Ugdh**	**0.911553**
		**230459**	**Cyp2j13**	**0.92393**
		**13110**	**Cyp2j6**	**0.955546**
		**66447**	**Mgst3**	**0.971084**

		**60525**	**Acss2**	**1.046677**
		**68738**	**Acss1**	**1.103326**
		**74134**	**Cyp2s1**	**1.216227**
		**211666**	**Mgst2**	**1.363034**
		**20887**	**Sult1a1**	**1.5804**
		**23972**	**Papss2**	**1.890541**
		**13521**	**Slc26a2**	**1.912715**
		**631304**	**Cyp4f40**	**1.947573**
		**13112**	**Cyp3a11**	**2.066924**
		**545123**	**Cyp2d11**	**2.074292**
		**17161**	**Maoa**	**2.081097**
		**13113**	**Cyp3a13**	**2.238127**
		**545288**	**Cyp2c67**	**2.508173**
		**13099**	**Cyp2c40**	**2.536402**
		**107141**	**Cyp2c50**	**2.555901**
		**17962**	**Nat3**	**2.585722**
		**76279**	**Cyp2d26**	**2.666213**
		**380997**	**Cyp2d12**	**2.687051**
		**13105**	**Cyp2d9**	**2.696041**
		**13101**	**Cyp2d10**	**2.835051**
		**56362**	**Sult1b1**	**3.038625**
		**13095**	**Cyp2c29**	**3.072696**
		**69888**	**Cyp2c66**	**3.206495**
		**1E+08**	**Cyp2c69**	**3.276957**
		**69083**	**Sult1c2**	**3.692611**
		**64385**	**Cyp4f14**	**3.873506**
		**72303**	**Cyp2c65**	**4.136602**
		**72082**	**Cyp2c55**	**4.559563**
	Xenobiotics	13088	Cyp2b10	-3.70625
		**13112**	**Cyp3a11**	**2.066924**
		**545123**	**Cyp2d11**	**2.074292**
		**13113**	**Cyp3a13**	**2.238127**
		**545288**	**Cyp2c67**	**2.508173**
		**13099**	**Cyp2c40**	**2.536402**
		**107141**	**Cyp2c50**	**2.555901**
		**76279**	**Cyp2d26**	**2.666213**
		**380997**	**Cyp2d12**	**2.687051**
		**13105**	**Cyp2d9**	**2.696041**
		**13101**	**Cyp2d10**	**2.835051**
		**13095**	**Cyp2c29**	**3.072696**
		**69888**	**Cyp2c66**	**3.206495**

		**1E+08**	**Cyp2c69**	**3.276957**
		**72303**	**Cyp2c65**	**4.136602**
		**72082**	**Cyp2c55**	**4.559563**
	Cytochrome P450 - arranged by substrate type	13088	Cyp2b10	-3.70625
		13123	Cyp7b1	-1.44706
		19223	Ptgis	-1.18808
		**230459**	**Cyp2j13**	**0.92393**
		**13110**	**Cyp2j6**	**0.955546**
		**74134**	**Cyp2s1**	**1.216227**
		**631304**	**Cyp4f40**	**1.947573**
		**13112**	**Cyp3a11**	**2.066924**
		**545123**	**Cyp2d11**	**2.074292**
		**13113**	**Cyp3a13**	**2.238127**
		**545288**	**Cyp2c67**	**2.508173**
		**13099**	**Cyp2c40**	**2.536402**
		**107141**	**Cyp2c50**	**2.555901**
		**76279**	**Cyp2d26**	**2.666213**
		**380997**	**Cyp2d12**	**2.687051**
		**13105**	**Cyp2d9**	**2.696041**
		**13101**	**Cyp2d10**	**2.835051**
		**13095**	**Cyp2c29**	**3.072696**
		**69888**	**Cyp2c66**	**3.206495**
		**1E+08**	**Cyp2c69**	**3.276957**
		**64385**	**Cyp4f14**	**3.873506**
		**72303**	**Cyp2c65**	**4.136602**
		**72082**	**Cyp2c55**	**4.559563**
	bupropion degradation	13088	Cyp2b10	-3.70625
		**13110**	**Cyp2j6**	**0.955546**
		**74134**	**Cyp2s1**	**1.216227**
		**13112**	**Cyp3a11**	**2.066924**
		**545123**	**Cyp2d11**	**2.074292**
		**13113**	**Cyp3a13**	**2.238127**
		**13099**	**Cyp2c40**	**2.536402**
		**107141**	**Cyp2c50**	**2.555901**
		**76279**	**Cyp2d26**	**2.666213**
		**13105**	**Cyp2d9**	**2.696041**
		**13101**	**Cyp2d10**	**2.835051**
		**13095**	**Cyp2c29**	**3.072696**
		**72303**	**Cyp2c65**	**4.136602**
		**72082**	**Cyp2c55**	**4.559563**
	nicotine degradation III	13088	Cyp2b10	-3.70625

		**94284**	**Ugt1a6a**	**0.900385**
		**13110**	**Cyp2j6**	**0.955546**
		**74134**	**Cyp2s1**	**1.216227**
		**13112**	**Cyp3a11**	**2.066924**
		**545123**	**Cyp2d11**	**2.074292**
		**13113**	**Cyp3a13**	**2.238127**
		**13099**	**Cyp2c40**	**2.536402**
		**107141**	**Cyp2c50**	**2.555901**
		**76279**	**Cyp2d26**	**2.666213**
		**13105**	**Cyp2d9**	**2.696041**
		**13101**	**Cyp2d10**	**2.835051**
		**13095**	**Cyp2c29**	**3.072696**
		**72303**	**Cyp2c65**	**4.136602**
		**72082**	**Cyp2c55**	**4.559563**
	Metabolism of xenobiotics by cytochrome P450	13088	Cyp2b10	-3.70625
		109857	Cbr3	-1.27599
		15483	Hsd11b1	-0.9844
		**103140**	**Gstt3**	**0.777197**
		**14872**	**Gstt2**	**0.813855**
		**94284**	**Ugt1a6a**	**0.900385**
		**66447**	**Mgst3**	**0.971084**
		**94215**	**Ugt2a1**	**0.97448**
		**76263**	**Gstk1**	**1.087932**
		**74134**	**Cyp2s1**	**1.216227**
		**394430**	**Ugt1a10**	**1.254373**
		**211666**	**Mgst2**	**1.363034**
		**13112**	**Cyp3a11**	**2.066924**
		**13113**	**Cyp3a13**	**2.238127**
		**13099**	**Cyp2c40**	**2.536402**
		**107141**	**Cyp2c50**	**2.555901**
		**76279**	**Cyp2d26**	**2.666213**
		**13105**	**Cyp2d9**	**2.696041**
		**100559**	**Ugt2b38**	**2.824694**
		**13101**	**Cyp2d10**	**2.835051**
		**13095**	**Cyp2c29**	**3.072696**
		**69888**	**Cyp2c66**	**3.206495**
		**433247**	**Cyp2c68**	**3.274499**
		**100727**	**Ugt2b34**	**3.486643**
		**72303**	**Cyp2c65**	**4.136602**
		**72082**	**Cyp2c55**	**4.559563**
	Drug metabolism - cytochrome P450	13088	Cyp2b10	-3.70625

		14261	Fmo1	-0.93541
		**103140**	**Gstt3**	**0.777197**
		**14872**	**Gstt2**	**0.813855**
		**94284**	**Ugt1a6a**	**0.900385**
		**66447**	**Mgst3**	**0.971084**
		**94215**	**Ugt2a1**	**0.97448**
		**76263**	**Gstk1**	**1.087932**
		**394430**	**Ugt1a10**	**1.254373**
		**211666**	**Mgst2**	**1.363034**
		**13112**	**Cyp3a11**	**2.066924**
		**17161**	**Maoa**	**2.081097**
		**13113**	**Cyp3a13**	**2.238127**
		**13099**	**Cyp2c40**	**2.536402**
		**107141**	**Cyp2c50**	**2.555901**
		**76279**	**Cyp2d26**	**2.666213**
		**13105**	**Cyp2d9**	**2.696041**
		**100559**	**Ugt2b38**	**2.824694**
		**13101**	**Cyp2d10**	**2.835051**
		**13095**	**Cyp2c29**	**3.072696**
		**69888**	**Cyp2c66**	**3.206495**
		**433247**	**Cyp2c68**	**3.274499**
		**100727**	**Ugt2b34**	**3.486643**
		**72303**	**Cyp2c65**	**4.136602**
		**72082**	**Cyp2c55**	**4.559563**
	nicotine degradation II	13088	Cyp2b10	-3.70625
		14261	Fmo1	-0.93541
		**94284**	**Ugt1a6a**	**0.900385**
		**13110**	**Cyp2j6**	**0.955546**
		**74134**	**Cyp2s1**	**1.216227**
		**13112**	**Cyp3a11**	**2.066924**
		**545123**	**Cyp2d11**	**2.074292**
		**13113**	**Cyp3a13**	**2.238127**
		**13099**	**Cyp2c40**	**2.536402**
		**107141**	**Cyp2c50**	**2.555901**
		**76279**	**Cyp2d26**	**2.666213**
		**13105**	**Cyp2d9**	**2.696041**
		**13101**	**Cyp2d10**	**2.835051**
		**13095**	**Cyp2c29**	**3.072696**
		**72303**	**Cyp2c65**	**4.136602**
		**72082**	**Cyp2c55**	**4.559563**
	Drug metabolism - other enzymes	80914	Uck2	-0.66786

		**17961**	**Nat2**	**0.824478**
		**22017**	**Tpmt**	**0.854172**
		**94284**	**Ugt1a6a**	**0.900385**
		**103149**	**Upb1**	**0.966679**
		**94215**	**Ugt2a1**	**0.97448**
		**72269**	**Cda**	**1.094769**
		**76654**	**Upp2**	**1.136651**
		**394430**	**Ugt1a10**	**1.254373**
		**13112**	**Cyp3a11**	**2.066924**
		**13113**	**Cyp3a13**	**2.238127**
		**17962**	**Nat3**	**2.585722**
		**100559**	**Ugt2b38**	**2.824694**
		**102022**	**Ces2a**	**2.836396**
		**234673**	**Ces2e**	**3.257719**
		**100727**	**Ugt2b34**	**3.486643**
		**234671**	**Ces2c**	**3.511133**
Metabolism	Nuclear receptors in lipid metabolism and toxicity	13088	Cyp2b10	-3.70625
		19411	Rarg	-1.03301
		11303	Abca1	-0.91568
		11307	Abcg1	-0.88402
		**19015**	**Ppard**	**0.729502**
		19013	***Ppara***	***1.763983***
		**76408**	**Abcc3**	**1.189916**
		**18670**	**Abcb4**	**1.326175**
		**18671**	**Abcb1a**	**1.373398**
		**20186**	**Nr1h4**	**1.42261**
		**19016**	**Pparg**	**1.539321**
		**22337**	**Vdr**	**1.692396**
		**18171**	**Nr1i2**	**2.63852**
	Prostaglandin Synthesis and Regulation	22287	Scgb1a1	-1.63652
		19225	Ptgs2	-1.60905
		19220	Ptgfr	-1.35934
		19223	Ptgis	-1.18808
		19218	Ptger3	-1.17597
		19222	Ptgir	-1.06711
		15483	Hsd11b1	-0.9844
		13617	Ednra	-0.86901
		19215	Ptgds	-0.6629
		19217	*Ptger2*	*-0.65162*
		**11746**	**Anxa4**	**0.932985**
		**15446**	**Hpgd**	**1.637187**

		**15484**	**Hsd11b2**	**2.433383**
	Synthesis of epoxy (EET) and dihydroxyeicosatrienoic acids (DHET)	**230459**	**Cyp2j13**	**0.92393**
		**13110**	**Cyp2j6**	**0.955546**
		**545288**	**Cyp2c67**	**2.508173**
		**13099**	**Cyp2c40**	**2.536402**
		**107141**	**Cyp2c50**	**2.555901**
		**13095**	**Cyp2c29**	**3.072696**
		**69888**	**Cyp2c66**	**3.206495**
		**1E+08**	**Cyp2c69**	**3.276957**
		**72303**	**Cyp2c65**	**4.136602**
		**72082**	**Cyp2c55**	**4.559563**
	Synthesis of (16-20)-hydroxyeicosatetraenoic acids (HETE)	**631304**	**Cyp4f40**	**1.947573**
		**545288**	**Cyp2c67**	**2.508173**
		**13099**	**Cyp2c40**	**2.536402**
		**107141**	**Cyp2c50**	**2.555901**
		**13095**	**Cyp2c29**	**3.072696**
		**69888**	**Cyp2c66**	**3.206495**
		**1E+08**	**Cyp2c69**	**3.276957**
		**64385**	**Cyp4f14**	**3.873506**
		**72303**	**Cyp2c65**	**4.136602**
		**72082**	**Cyp2c55**	**4.559563**
	Ascorbate and aldarate metabolism	**11671**	**Aldh3a2**	**0.779767**
		**11669**	**Aldh2**	**0.818158**
		**94284**	**Ugt1a6a**	**0.900385**
		**22235**	**Ugdh**	**0.911553**
		**94215**	**Ugt2a1**	**0.97448**
		**394430**	**Ugt1a10**	**1.254373**
		**72535**	**Aldh1b1**	**1.830414**
		**100559**	**Ugt2b38**	**2.824694**
		**100727**	**Ugt2b34**	**3.486643**
	Linoleic acid metabolism	26970	Pla2g2e	-1.55384
		66350	Pla2g12a	-0.81203
		**230459**	**Cyp2j13**	**0.92393**
		**13110**	**Cyp2j6**	**0.955546**
		**26565**	**Pla2g10**	**1.218373**
		**69836**	**Pla2g12b**	**1.34028**
		**18781**	**Pla2g2c**	**1.949176**
		**13112**	**Cyp3a11**	**2.066924**
		**13113**	**Cyp3a13**	**2.238127**
		**13099**	**Cyp2c40**	**2.536402**
		**107141**	**Cyp2c50**	**2.555901**

		**13095**	**Cyp2c29**	**3.072696**
		**69888**	**Cyp2c66**	**3.206495**
		**433247**	**Cyp2c68**	**3.274499**
		**72303**	**Cyp2c65**	**4.136602**
		**72082**	**Cyp2c55**	**4.559563**
	Arachidonic acid metabolism	13088	Cyp2b10	-3.70625
		320997	Cyp4f39	-2.49348
		11686	Alox12b	-2.12341
		64292	Ptges	-1.95058
		319446	Dpep2	-1.78526
		11690	Alox5ap	-1.69206
		19225	Ptgs2	-1.60905
		26970	Pla2g2e	-1.55384
		109857	Cbr3	-1.27599
		19223	Ptgis	-1.18808
		14778	Gpx3	-1.14709
		11689	Alox5	-1.04672
		67305	Gpx7	-1.03756
		69590	Gpx8	-0.91385
		66469	Fam213b	-0.86354
		66350	Pla2g12a	-0.81203
		19215	Ptgds	-0.6629
		67103	Ptgr1	-0.6351
		**208285**	**Cyp4f17**	**0.707887**
		**170716**	**Cyp4f13**	**0.83413**
		**230459**	**Cyp2j13**	**0.92393**
		**13110**	**Cyp2j6**	**0.955546**
		**26565**	**Pla2g10**	**1.218373**
		**69836**	**Pla2g12b**	**1.34028**
		**15446**	**Hpgd**	**1.637187**
		**27384**	**Akr1c13**	**1.733167**
		**622402**	**Akr1c12**	**1.847407**
		**631304**	**Cyp4f40**	**1.947573**
		**18781**	**Pla2g2c**	**1.949176**
		**545288**	**Cyp2c67**	**2.508173**
		**13099**	**Cyp2c40**	**2.536402**
		**107141**	**Cyp2c50**	**2.555901**
		**105387**	**Akr1c14**	**2.737795**
		**432720**	**Akr1c19**	**2.895549**
		**13095**	**Cyp2c29**	**3.072696**
		**69888**	**Cyp2c66**	**3.206495**

		**433247**	**Cyp2c68**	**3.274499**
		**1E+08**	**Cyp2c69**	**3.276957**
		**64385**	**Cyp4f14**	**3.873506**
		**72303**	**Cyp2c65**	**4.136602**
		**72082**	**Cyp2c55**	**4.559563**
	Synthesis of Prostaglandins (PG) and Thromboxanes (TX)	64292	Ptges	-1.95058
		19225	Ptgs2	-1.60905
		19223	Ptgis	-1.18808
		66469	Fam213b	-0.86354
		19215	Ptgds	-0.6629
		**15446**	**Hpgd**	**1.637187**
		**27384**	**Akr1c13**	**1.733167**
		**622402**	**Akr1c12**	**1.847407**
		**105387**	**Akr1c14**	**2.737795**
		**432720**	**Akr1c19**	**2.895549**
	Regulation of Insulin-like Growth Factor (IGF) Transport and Uptake by Insulin-like Growth Factor Binding Proteins (IGFBPs)	18491	Pappa	-1.63629
		16008	Igfbp2	-1.45576
		16010	Igfbp4	-1.17088
		16012	Igfbp6	-0.97725
		17390	*Mmp2*	*-0.90074*
		**16616**	**Klk1b21**	**1.750316**
		**16622**	**Klk1b5**	**2.161531**
		**16617**	**Klk1b24**	**2.162845**
		**18048**	**Klk1b4**	**2.265681**
		**16624**	**Klk1b8**	**2.270613**
		**16618**	**Klk1b26**	**2.295813**
		**18050**	**Klk1b3**	**2.316179**
		**16612**	**Klk1**	**2.390543**
		**13646**	**Klk1b22**	**2.562583**
	Propanoate metabolism	16832	Ldhb	-1.049013
		**106557**	**Ldhal6b**	**0.7084865**
		**17850**	**Mut**	**0.7362996**
		**11671**	**Aldh3a2**	**0.779767**
		**11669**	**Aldh2**	**0.8181583**
		**66904**	**Pccb**	**0.8738388**
		**60525**	**Acss2**	**1.0466773**
		**74147**	**Ehhadh**	**1.0968974**
		**68738**	**Acss1**	**1.1033255**
		**268860**	**Abat**	**1.1681836**
		**20917**	**Suclg2**	**1.4169702**
		**72535**	**Aldh1b1**	**1.8304137**

	UDP-N-acetyl-D-glucosamine biosynthesis II	14584	Gfpt2	-1.786155
		**66681**	**Pgm1**	**0.6577726**
		**109785**	**Pgm3**	**0.7860824**
		**54342**	**Gnpnat1**	**0.7916704**
		**14583**	**Gfpt1**	**1.4531144**
Metabolism of proteins	O-linked glycosylation of mucins	108148	Galnt2	-1.04769
		78754	Galnt15	-0.99068
		232984	B3gnt8	-0.59967
		**59048**	**C1galt1c1**	**0.679343**
		**14426**	**Galnt4**	**0.916186**
		**94192**	**C1galt1**	**0.953988**
		**171212**	**Galnt10**	**0.969919**
		**140474**	**Muc4**	**1.024719**
		**20441**	**St3gal3**	**1.033166**
		**72297**	**B3gnt3**	**1.106007**
		**230145**	**Galnt12**	**1.201133**
		**108150**	**Galnt7**	**1.37418**
		**207839**	**Galnt6**	**1.483364**
		**17063**	**Muc13**	**1.729689**
		**72077**	**Gcnt3**	**2.629908**
		**241391**	**Galnt5**	**2.719413**
	Mucin type O-Glycan biosynthesis	108760	Galnt16	-1.84967
		108148	Galnt2	-1.04769
		78754	Galnt15	-0.99068
		233733	Galnt18	-0.90069
		**59048**	**C1galt1c1**	**0.679343**
		**14426**	**Galnt4**	**0.916186**
		**94192**	**C1galt1**	**0.953988**
		**171212**	**Galnt10**	**0.969919**
		**230145**	**Galnt12**	**1.201133**
		**270049**	**Galntl6**	**1.266109**
		**108150**	**Galnt7**	**1.37418**
		**207839**	**Galnt6**	**1.483364**
		**72077**	**Gcnt3**	**2.629908**
		**241391**	**Galnt5**	**2.719413**
Immune response	Cytokine-cytokine receptor interaction	*20311*	*Cxcl5*	*-4.34495*
		14825	Cxcl1	-3.66321
		16156	Il11	-3.30123
		12765	*Cxcr2*	*-3.17533*
		16324	Inhbb	-2.62677
		18413	Osm	-2.42429

		16176	Il1b	-2.39883
		16323	Inhba	-2.3647
		20296	*Ccl2*	*-2.33834*
		20306	Ccl7	-2.30947
		16171	Il17a	-2.10969
		14205	Figf	-1.92988
		329244	Il19	-1.89374
		16178	Il1r2	-1.81448
		20303	Ccl4	-1.80734
		237313	Il20ra	-1.72651
		12778	Ackr3	-1.56997
		27279	Tnfrsf12a	-1.4118
		213208	Il20rb	-1.40338
		12984	Csf2rb2	-1.375
		12804	Cntfr	-1.34321
		14254	Flt1	-1.28931
		18414	Osmr	-1.27205
		12981	Csf2	-1.26039
		16174	Il18rap	-1.23762
		21950	Tnfsf9	-1.21694
		56221	Ccl24	-1.19359
		12772	Ccr2	-1.18817
		12162	Bmp7	-1.15597
		21809	Tgfb3	-1.13117
		50930	Tnfsf14	-1.13101
		20292	Ccl11	-1.08681
		16177	Il1r1	-1.07659
		12774	Ccr5	-1.06964
		12167	Bmpr1b	-1.05427
		56744	Pf4	-1.01392
		21936	Tnfrsf18	-0.98226
		12776	Ccr8	-0.96612
		245527	Eda2r	-0.95375
		13607	Eda	-0.94984
		20308	Ccl9	-0.9276
		18596	Pdgfrb	-0.9223
		57266	Cxcl14	-0.81335
		15976	Ifnar2	-0.79849
		16195	Il6st	-0.78633
		16188	Il3ra	-0.77416
		16157	Il11ra1	-0.73559

		21944	Tnfsf12	-0.73266
		16190	Il4ra	-0.63756
		11477	Acvr1	-0.63677
		15968	Ifna5	-0.61563
		1E+08	Gm20878	-0.60711
		21937	Tnfrsf1a	-0.60442
		12977	Csf1	-0.60055
		**12986**	**Csf3r**	**0.59828**
		**326623**	**Tnfsf15**	**0.694025**
		**21934**	**Tnfrsf11a**	**0.695412**
		**16590**	**Kit**	**0.766462**
		**12168**	**Bmpr2**	**0.800831**
		**13051**	**Cx3cr1**	**1.128915**
		**230828**	**Il22ra1**	**1.173466**
		**16168**	**Il15**	**1.309923**
		**16173**	**Il18**	**1.377159**
		**16963**	**Xcl1**	**1.565947**
		**56838**	**Ccl28**	**1.79597**
		**387510**	**Ifnk**	**1.903833**
		**23832**	**Xcr1**	**2.111892**
	Complement and coagulation cascades	18787	Serpine1	-2.602674
		14058	F10	-1.842658
		12273	C5ar1	-1.780122
		18791	Plat	-1.499046
		18793	Plaur	-1.447755
		17174	Masp1	-1.230056
		19128	Pros1	-1.020553
		12628	Cfh	-1.014873
		12268	C4b	-0.937435
		18792	Plau	-0.884646
		50908	C1s1	-0.867112
		12258	Serping1	-0.856848
		14067	F5	-0.836852
		12260	C1qb	-0.717686
		12267	C3ar1	-0.682265
		12259	C1qa	-0.666613
		12061	Bdkrb1	-0.603269
		21824	Thbd	-0.593737
		**56373**	**Cpb2**	**1.0812315**
		**19123**	**Proc**	**1.383527**
		**12269**	**C4bp**	**1.5648379**

	Staphylococcus aureus infection	14293	Fpr1	-3.03344
		14289	Fpr2	-2.50436
		20344	Selp	-2.04701
		12273	C5ar1	-1.78012
		246256	Fcgr4	-1.63746
		14131	Fcgr3	-1.25112
		17174	Masp1	-1.23006
		14130	Fcgr2b	-1.22614
		14129	Fcgr1	-1.09932
		12628	Cfh	-1.01487
		12268	C4b	-0.93744
		317677	C1s2	-0.9368
		50908	C1s1	-0.86711
		19204	Ptafr	-0.72302
		12260	C1qb	-0.71769
		12267	C3ar1	-0.68227
		12259	C1qa	-0.66661
Cancer signalling	Chemical carcinogenesis	13088	Cyp2b10	-3.70625
		19225	Ptgs2	-1.60905
		15483	Hsd11b1	-0.9844
		**103140**	**Gstt3**	**0.777197**
		**14872**	**Gstt2**	**0.813855**
		**17961**	**Nat2**	**0.824478**
		**94284**	**Ugt1a6a**	**0.900385**
		**66447**	**Mgst3**	**0.971084**
		**94215**	**Ugt2a1**	**0.97448**
		**76263**	**Gstk1**	**1.087932**
		**394430**	**Ugt1a10**	**1.254373**
		**211666**	**Mgst2**	**1.363034**
		**20887**	**Sult1a1**	**1.5804**
		**13112**	**Cyp3a11**	**2.066924**
		**13113**	**Cyp3a13**	**2.238127**
		**13099**	**Cyp2c40**	**2.536402**
		**107141**	**Cyp2c50**	**2.555901**
		**17962**	**Nat3**	**2.585722**
		**100559**	**Ugt2b38**	**2.824694**
		**13095**	**Cyp2c29**	**3.072696**
		**69888**	**Cyp2c66**	**3.206495**
		**433247**	**Cyp2c68**	**3.274499**
		**100727**	**Ugt2b34**	**3.486643**
		**72303**	**Cyp2c65**	**4.136602**

		**72082**	**Cyp2c55**	**4.559563**
	FGFR3c ligand binding and activation<br>Signaling by FGFR3 mutants<br>FGFR3 ligand binding and activation<br>Signaling by activated point mutants of FGFR3	14172	Fgf18	-1.82915
		14173	*Fgf2*	*-1.4179*
		**14180**	**Fgf9**	**1.024348**
		**64654**	**Fgf23**	**1.226145**
		**14179**	**Fgf8**	**1.751112**
	FGFR1c ligand binding and activation<br>Signaling by activated point mutants of FGFR1	*14173*	*Fgf2*	*-1.4179*
		14182	Fgfr1	-0.69788
		**14180**	**Fgf9**	**1.024348**
		**64654**	**Fgf23**	**1.226145**
		**14179**	**Fgf8**	**1.751112**
Haemostasis	Common Pathway of Fibrin Clot Formation	14058	F10	-1.84266
		74145	F13a1	-1.41525
		19128	Pros1	-1.02055
		56744	Pf4	-1.01392
		14067	F5	-0.83685
		21824	Thbd	-0.59374
		**19123**	**Proc**	**1.383527**
	Cell surface interactions at the vascular wall	20344	Selp	-2.04701
		11602	Angpt4	-1.97774
		80879	Slc16a3	-1.57048
		14268	Fn1	-1.48474
		16402	Itga5	-1.3317
		67374	Jam2	-1.05142
		14127	Fcer1g	-1.02748
		19128	Pros1	-1.02055
		56744	Pf4	-1.01392
		69524	Esam	-0.9507
		330836	Slc7a6	-0.78741
		13449	Dok2	-0.7873
		11933	Atp1b3	-0.76755
		16412	Itgb1	-0.67394
		83964	Jam3	-0.61841
		11421	*Ace*	*-0.642567*
		270152	Amica1	-0.61308
		21824	Thbd	-0.59374
		**16653**	**Kras**	**0.827463**
		**16728**	**L1cam**	**0.83648**
		**14786**	**Grb7**	**1.147856**
		**11931**	**Atp1b1**	**1.177377**
		**13052**	**Cxadr**	**1.211875**

		**19123**	**Proc**	**1.383527**
		**30962**	**Slc7a9**	**1.507333**
		**18706**	**Pik3ca**	**1.94193**
Extracellular matrix organisation	Extracellular matrix organization	103964	Try5	-6.95841
		22074	Try4	-6.80598
		22072	Prss2	-6.4655
		1E+08	Gm10334	-5.43591
		17384	Mmp10	-3.57344
		22073	Prss3	-3.56028
		17394	Mmp8	-3.34127
		17386	Mmp13	-3.04753
		18787	Serpine1	-2.60267
		16948	Lox	-2.53793
		21857	Timp1	-2.07328
		17395	*Mmp9*	*-1.95523*
		50530	Mfap5	-1.55405
		14268	Fn1	-1.48474
		23794	Adamts5	-1.48121
		13038	Ctsk	-1.42001
		14173	*Fgf2*	*-1.4179*
		12837	Col8a1	-1.36969
		16776	Lama5	-1.33705
		16402	Itga5	-1.3317
		16949	Loxl1	-1.26522
		12842	Col1a1	-1.2469
		18074	Nid2	-1.24567
		14115	Fbln2	-1.2306
		12831	Col5a1	-1.22051
		17228	Cma1	-1.19147
		237360	Adamts14	-1.19042
		21825	Thbs1	-1.16147
		12162	Bmp7	-1.15597
		58223	Mmp19	-1.14639
		12827	Col4a2	-1.12303
		14118	Fbn1	-1.10411
		20692	Sparc	-1.07947
		67374	Jam2	-1.05142
		58859	Efemp2	-1.04386
		12826	Col4a1	-1.04373
		12822	Col18a1	-0.99187
		18214	Ddr2	-0.98714

		17381	Mmp12	-0.97331
		16416	Itgb3	-0.9521
		56693	Crtap	-0.93321
		17229	Tpsb2	-0.90222
		17390	*Mmp2*	*-0.90074*
		12153	Bmp1	-0.88652
		16399	Itga2b	-0.8846
		17387	Mmp14	-0.88225
		17183	Matn4	-0.876
		14789	P3h3	-0.8704
		12111	Bgn	-0.86889
		18542	Pcolce	-0.85151
		78369	Icam4	-0.84185
		12154	Bmp10	-0.83219
		23876	Fbln5	-0.81925
		16998	Ltbp3	-0.79868
		26432	Plod2	-0.75722
		13039	Ctsl	-0.75546
		226519	Lamc1	-0.75043
		216725	Adamts2	-0.72634
		12832	Col5a2	-0.70187
		16412	Itgb1	-0.67394
		21858	Timp2	-0.65079
		13003	Vcan	-0.6463
		76293	Mfap4	-0.64341
		15530	Hspg2	-0.63594
		83964	Jam3	-0.61841
		12814	Col11a1	-0.59561
		13179	Dcn	-0.58753
		**17388**	**Mmp15**	**0.646751**
		**16780**	**Lamb3**	**0.779921**
		**19164**	**Psen1**	**0.797152**
		**18750**	**Prkca**	**0.853424**
		**12550**	**Cdh1**	**0.894417**
		**16420**	**Itgb6**	**0.946717**
		**12367**	**Casp3**	**1.014783**
		**213119**	**Itga10**	**1.186335**
	Degradation of the extracellular matrix	103964	Try5	-6.95841
		22074	Try4	-6.80598
		22072	Prss2	-6.4655
		100040233	Gm10334	-5.43591

		17384	Mmp10	-3.57344
		22073	Prss3	-3.56028
		17394	Mmp8	-3.34127
		17386	Mmp13	-3.04753
		21857	Timp1	-2.07328
		17395	*Mmp9*	*-1.95523*
		23794	Adamts5	-1.48121
		13038	Ctsk	-1.42001
		16776	Lama5	-1.33705
		17228	Cma1	-1.19147
		58223	Mmp19	-1.14639
		12822	Col18a1	-0.99187
		17381	Mmp12	-0.97331
		17229	Tpsb2	-0.90222
		17390	*Mmp2*	*-0.90074*
		12153	Bmp1	-0.88652
		17387	Mmp14	-0.88225
		13039	Ctsl	-0.75546
		226519	Lamc1	-0.75043
		21858	Timp2	-0.65079
		15530	Hspg2	-0.63594
		13179	Dcn	-0.58753
		**17388**	**Mmp15**	**0.646751**
		**16780**	**Lamb3**	**0.779921**
		**19164**	**Psen1**	**0.797152**
		**12550**	**Cdh1**	**0.894417**
		**12367**	**Casp3**	**1.014783**
	Activation of Matrix Metalloproteinases	103964	Try5	-6.95841
		22074	Try4	-6.80598
		22072	Prss2	-6.4655
		100040233	Gm10334	-5.43591
		17384	Mmp10	-3.57344
		22073	Prss3	-3.56028
		17386	Mmp13	-3.04753
		21857	Timp1	-2.07328
		17395	*Mmp9*	*-1.95523*
		13038	Ctsk	-1.42001
		17228	Cma1	-1.19147
		12822	Col18a1	-0.99187
		17229	Tpsb2	-0.90222
		17390	*Mmp2*	*-0.90074*

		17387	Mmp14	-0.88225
		13039	Ctsl	-0.75546
		21858	Timp2	-0.65079
	Matrix Metalloproteinases	17384	Mmp10	-3.57344
		17394	Mmp8	-3.34127
		17386	Mmp13	-3.04753
		21857	Timp1	-2.07328
		17395	*Mmp9*	*-1.95523*
		58223	Mmp19	-1.14639
		17381	Mmp12	-0.97331
		17390	*Mmp2*	*-0.90074*
		17387	Mmp14	-0.88225
		21859	Timp3	-0.70261
		26561	Mmp23	-0.67758
		21858	Timp2	-0.65079
		**17388**	**Mmp15**	**0.646751**
	Elastic fibre formation	50530	Mfap5	-1.55405
		16402	Itga5	-1.3317
		14115	Fbln2	-1.2306
		12162	Bmp7	-1.15597
		14118	Fbn1	-1.10411
		58859	Efemp2	-1.04386
		16416	Itgb3	-0.9521
		12154	Bmp10	-0.83219
		23876	Fbln5	-0.81925
		16998	Ltbp3	-0.79868
		16412	Itgb1	-0.67394
		76293	Mfap4	-0.64341
		**16420**	**Itgb6**	**0.946717**
	Collagen formation	17384	Mmp10	-3.57344
		17386	Mmp13	-3.04753
		16948	Lox	-2.53793
		17395	*Mmp9*	*-1.95523*
		12837	Col8a1	-1.36969
		16949	Loxl1	-1.26522
		12842	Col1a1	-1.2469
		12831	Col5a1	-1.22051
		237360	Adamts14	-1.19042
		12827	Col4a2	-1.12303
		12826	Col4a1	-1.04373
		12822	Col18a1	-0.99187

		56693	Crtap	-0.93321
		12153	Bmp1	-0.88652
		14789	P3h3	-0.8704
		18542	Pcolce	-0.85151
		26432	Plod2	-0.75722
		13039	Ctsl	-0.75546
		216725	Adamts2	-0.72634
		12832	Col5a2	-0.70187
		12814	Col11a1	-0.59561
		**16780**	**Lamb3**	**0.779921**
	Assembly of collagen fibrils and other multimeric structures	17384	Mmp10	-3.57344
		17386	Mmp13	-3.04753
		16948	Lox	-2.53793
		17395	*Mmp9*	*-1.95523*
		12837	Col8a1	-1.36969
		16949	Loxl1	-1.26522
		12827	Col4a2	-1.12303
		12826	Col4a1	-1.04373
		12822	Col18a1	-0.99187
		12153	Bmp1	-0.88652
		13039	Ctsl	-0.75546
		**16780**	**Lamb3**	**0.779921**
Digestive system	Bile secretion	104110	Adcy4	-1.16978
		27222	Atp1a4	-1.09805
		239273	Abcc4	-1.08389
		210044	Adcy2	-0.99756
		64008	Aqp9	-0.95331
		11933	Atp1b3	-0.76755
		**320024**	**Nceh1**	**0.717925**
		**20537**	**Slc5a1**	**0.762464**
		**11928**	**Atp1a1**	**0.814248**
		**26357**	**Abcg2**	**0.910726**
		**18669**	**Abcb1b**	**0.947346**
		**11512**	**Adcy6**	**0.967657**
		**11931**	**Atp1b1**	**1.177377**
		**76408**	**Abcc3**	**1.189916**
		**12349**	**Car2**	**1.218899**
		**18670**	**Abcb4**	**1.326175**
		**18671**	**Abcb1a**	**1.373398**
		**20186**	**Nr1h4**	**1.42261**
		**20517**	**Slc22a1**	**1.679911**

		**12638**	**Cftr**	**1.952521**
		**28254**	**Slco1a6**	**1.98067**
		**330962**	**Slc51b**	**2.335887**
		**20494**	**Slc10a2**	**2.71532**
		**105243**	**Slc9a3**	**2.762093**
		**11833**	**Aqp8**	**3.844864**
		**106407**	**Slc51a**	**4.704848**
	Pancreatic secretion	103964	Try5	-6.958405
		22074	Try4	-6.805979
		109697	Cpa1	-6.506434
		22072	Prss2	-6.465498
		1E+08	Gm10334	-5.435911
		18946	Pnliprp1	-5.269375
		12613	Cel	-4.981617
		11937	Atp2a1	-1.741202
		26970	Pla2g2e	-1.553839
		12182	Bst1	-1.527494
		104110	Adcy4	-1.169776
		27222	Atp1a4	-1.098046
		210044	Adcy2	-0.997562
		66350	Pla2g12a	-0.812027
		11933	Atp1b3	-0.76755
		**16440**	**Itpr3**	**0.6272043**
		**67972**	**Atp2b1**	**0.6699104**
		**80718**	**Rab27b**	**0.6804285**
		**11928**	**Atp1a1**	**0.8142482**
		**18750**	**Prkca**	**0.8534241**
		**11512**	**Adcy6**	**0.9676566**
		**56373**	**Cpb2**	**1.0812315**
		**11931**	**Atp1b1**	**1.1773769**
		**26565**	**Pla2g10**	**1.2183728**
		**12349**	**Car2**	**1.2188992**
		**69836**	**Pla2g12b**	**1.3402796**
		**53313**	**Atp2a3**	**1.7509815**
		**18781**	**Pla2g2c**	**1.9491762**
		**12638**	**Cftr**	**1.9525208**
		**13487**	**Slc26a3**	**2.3676917**
		**23844**	**Clca1**	**4.0815394**
	Amino sugar and nucleotide sugar metabolism	14584	Gfpt2	-1.78616
		19703	Renbp	-0.86803
		212032	Hk3	-0.73564

		**110119**	**Mpi**	**0.632236**
		**66681**	**Pgm1**	**0.657773**
		**234730**	**Fuk**	**0.666565**
		**94181**	**Nans**	**0.695492**
		**22122**	**Tsta3**	**0.721833**
		**75540**	**Fpgt**	**0.783654**
		**109785**	**Pgm3**	**0.786082**
		**54342**	**Gnpnat1**	**0.79167**
		**69976**	**Galk2**	**0.798675**
		**12764**	**Cmas**	**0.861057**
		**22235**	**Ugdh**	**0.911553**
		**74246**	**Gale**	**1.07737**
		**54128**	**Pmm2**	**1.235963**
		**14583**	**Gfpt1**	**1.453114**
		**50798**	**Gne**	**1.507443**
		**218138**	**Gmds**	**1.515681**
	Protein digestion and absorption	103964	Try5	-6.95841
		22074	Try4	-6.80598
		109697	Cpa1	-6.50643
		22072	Prss2	-6.4655
		1E+08	Gm10334	-5.43591
		17380	Mme	-1.71403
		12842	Col1a1	-1.2469
		12831	Col5a1	-1.22051
		12827	Col4a2	-1.12303
		27222	Atp1a4	-1.09805
		12826	Col4a1	-1.04373
		12822	Col18a1	-0.99187
		12818	Col14a1	-0.81313
		11933	Atp1b3	-0.76755
		12832	Col5a2	-0.70187
		12814	Col11a1	-0.59561
		**110893**	**Slc8a3**	**0.744391**
		**11928**	**Atp1a1**	**0.814248**
		**170745**	**Xpnpep2**	**1.009864**
		**56373**	**Cpb2**	**1.081231**
		**11931**	**Atp1b1**	**1.177377**
		**17288**	**Mep1b**	**1.317645**
		**57442**	**Kcne3**	**1.326414**
		**30962**	**Slc7a9**	**1.507333**
		**16529**	**Kcnk5**	**1.67479**

		**105243**	**Slc9a3**	**2.762093**
		**20532**	**Slc3a1**	**3.223735**
		**17287**	**Mep1a**	**4.972591**
	Mineral absorption	320718	Slc26a9	-3.26554
		15368	Hmox1	-1.6416
		27222	Atp1a4	-1.09805
		18173	Slc11a1	-1.04758
		17748	Mt1	-1.03798
		74051	Steap2	-0.91737
		70358	Steap1	-0.81693
		11933	Atp1b3	-0.76755
		**67972**	**Atp2b1**	**0.66991**
		**20537**	**Slc5a1**	**0.762464**
		**11928**	**Atp1a1**	**0.814248**
		**11931**	**Atp1b1**	**1.177377**
		**15203**	**Heph**	**1.252356**
		**72027**	**Slc39a4**	**1.359697**
		**22337**	**Vdr**	**1.692396**
		**53945**	**Slc40a1**	**1.796217**
		**225997**	**Trpm6**	**1.834091**
		**12724**	**Clcn2**	**2.092737**
		**13487**	**Slc26a3**	**2.367692**
		**105243**	**Slc9a3**	**2.762093**
		**12309**	**S100g**	**4.31916**
Endocrine and metabolic disease	Maturity onset diabetes of the young	**21410**	**Hnf1b**	**0.742425**
		**15205**	**Hes1**	**0.823965**
		**18508**	**Pax6**	**0.887141**
		**21405**	**Hnf1a**	**1.191427**
		**15377**	**Foxa3**	**1.231398**
		**30942**	**Hnf4g**	**1.294742**
		**18770**	**Pklr**	**1.664678**
		**18506**	**Pax4**	**1.687499**
		**15376**	**Foxa2**	**1.894105**
		**18012**	**Neurod1**	**2.067547**
		**26424**	**Nr5a2**	**2.1028**
		**18088**	**Nkx2-2**	**2.111146**
		**15378**	**Hnf4a**	**2.913687**
Muscle contraction	Striated Muscle Contraction	21925	Tnnc2	-4.38071
		21957	Tnnt3	-4.1188
		17897	Myl3	-3.65672
		17996	Neb	-3.50566

		17885	Myh8	-3.40606
		21393	Tcap	-3.13716
		233199	Mybpc2	-2.922
		21953	Tnni2	-2.80414
		17901	Myl1	-2.50815
		11472	Actn2	-2.42883
		109272	Mybpc1	-2.17576
		22138	Ttn	-1.98538
		21924	Tnnc1	-0.96121
		22352	Vim	-0.7463
		17868	Mybpc3	-0.60747
		21952	Tnni1	1.979125
	Muscle contraction	21925	Tnnc2	-4.38071
		21957	Tnnt3	-4.1188
		17897	Myl3	-3.65672
		17996	Neb	-3.50566
		17885	Myh8	-3.40606
		21393	Tcap	-3.13716
		233199	Mybpc2	-2.922
		21953	Tnni2	-2.80414
		17901	Myl1	-2.50815
		11472	Actn2	-2.42883
		109272	Mybpc1	-2.17576
		22138	Ttn	-1.98538
		21924	Tnnc1	-0.96121
		22352	Vim	-0.7463
		17868	Mybpc3	-0.60747
		12313	Calm1	0.865731
		21952	Tnni1	1.979125
Bone development	Endochondral Ossification	17386	Mmp13	-3.04753
		11504	Adamts1	-2.10667
		17395	*Mmp9*	*-1.95523*
		14172	Fgf18	-1.82915
		18791	Plat	-1.49905
		23794	Adamts5	-1.48121
		14173	*Fgf2*	*-1.4179*
		58250	Chst11	-1.36561
		11647	Alpl	-1.22751
		12162	Bmp7	-1.15597
		19227	Pthlh	-1.14587
		17313	Mgp	-1.01976

		18214	Ddr2	-0.98714
		18792	Plau	-0.88465
		12577	Cdkn1c	-0.84685
		13039	Ctsl	-0.75546
		14634	Gli3	-0.74002
		21859	Timp3	-0.70261
		14182	Fgfr1	-0.69788
		19228	Pth1r	-0.61805
		12283	Cab39	0.755627
		12313	Calm1	0.865731
		20682	Sox9	1.271387
		20259	Scin	1.945651
		16147	Ihh	2.452108
	Endocrine and other factor-regulated calcium reabsorption	27222	Atp1a4	-1.09805
		12062	*Bdkrb2*	*-0.94312*
		11933	Atp1b3	-0.76755
		19228	Pth1r	-0.61805
		13982	*Esr1*	*-0.60087*
		67972	Atp2b1	0.66991
		11928	Atp1a1	0.814248
		18750	Prkca	0.853424
		11512	Adcy6	0.967657
		11931	Atp1b1	1.177377
		22337	Vdr	1.692396
		16616	Klk1b21	1.750316
		16622	Klk1b5	2.161531
		16617	Klk1b24	2.162845
		16624	Klk1b8	2.270613
		16618	Klk1b26	2.295813
		18050	Klk1b3	2.316179
		16612	Klk1	2.390543
		13646	Klk1b22	2.562583
	Calcium signalling pathway	21925	Tnnc2	-4.38071
		228785	Mylk2	-2.14324
		20698	*Sphk1*	*-1.83599*
		12292	Cacna1s	-1.83224
		11541	Adora2b	-1.82001
		11937	Atp2a1	-1.7412
		12182	Bst1	-1.52749
		54140	Avpr1a	-1.40933
		19220	Ptgfr	-1.35934

		18127	Nos3	-1.23655
		20190	Ryr1	-1.18304
		19218	Ptger3	-1.17597
		104110	Adcy4	-1.16978
		21336	Tacr1	-1.16015
		210044	Adcy2	-0.99756
		94045	P2rx5	-0.96585
		21924	Tnnc1	-0.96121
		12062	*Bdkrb2*	*-0.94312*
		18596	Pdgfrb	-0.9223
		13617	Ednra	-0.86901
		11555	Adrb2	-0.84979
		11540	Adora2a	-0.8378
		18439	P2rx7	-0.81755
		11739	Slc25a4	-0.7536
		19204	Ptafr	-0.72302
		12061	Bdkrb1	-0.60327
		18575	Pde1c	0.585316
		16440	Itpr3	0.627204
		67972	Atp2b1	0.66991
		102093	Phkb	0.676941
		20866	Stim1	0.683955
		110893	Slc8a3	0.744391
		13866	*Erbb2*	*0.817359*
		18750	Prkca	0.853424
		12313	Calm1	0.865731
		74055	Plce1	0.876211
		14672	Gna11	0.925661
		15562	Htr4	0.991937
		13867	Erbb3	1.114448
		18679	Phka1	1.193311
		21337	Tacr2	1.265931
		54652	Cacna1f	1.342953
		228550	Itpka	1.497681
		53313	Atp2a3	1.750981
		16867	Lhcgr	1.871822
		14829	Grpr	2.004654
Transport of molecules	Transport of glucose and other sugars, bile salts and organic acids, metal ions and amine compounds	75750	Slc10a6	-2.13402
		80879	Slc16a3	-1.57048
		14411	Slc6a12	-1.46674
		12870	Cp	-1.32586

		230810	Slc30a2	-1.29338
		213053	Slc39a14	-1.29001
		30805	Slc22a4	-1.19745
		18173	Slc11a1	-1.04758
		227059	Slc39a10	-0.94369
		106957	Slc39a6	-0.83374
		20527	Slc2a3	-0.77539
		353169	Slc2a12	-0.75386
		56517	Slc22a21	0.635002
		20520	Slc22a5	0.676375
		20537	Slc5a1	0.762464
		233836	Slc5a11	1.052542
		53881	Slc5a3	1.053722
		213603	Slc44a3	1.11154
		15203	Heph	1.252356
		72027	Slc39a4	1.359697
		18400	Slc22a18	1.470949
		20517	Slc22a1	1.679911
		110877	Slc18a1	1.691706
		53945	Slc40a1	1.796217
		70129	Slc44a4	2.016854
		72002	Slc39a5	2.180762
		55961	Slc13a1	2.29241
		56774	Slc6a14	2.523776
		20500	Slc13a2	3.892954
	Ion channel transport	11937	Atp2a1	-1.7412
		20190	Ryr1	-1.18304
		22068	Trpc6	-1.17226
		27222	Atp1a4	-1.09805
		54667	Atp8b2	-1.03066
		63873	Trpv4	-0.95688
		17178	Fxyd3	-0.93915
		11982	Atp10a	-0.90694
		11933	Atp1b3	-0.76755
		105722	Ano6	-0.75203
		56188	Fxyd1	-0.69905
		14605	Tsc22d3	-0.69762
		20276	Scnn1a	0.599167
		12725	Clcn3	0.609869
		67972	Atp2b1	0.66991
		338370	Nalcn	0.743838

		76295	Atp11b	0.76297
		102566	Ano10	0.769236
		69847	Wnk4	0.805242
		11928	Atp1a1	0.814248
		71345	Ano9	0.888618
		329178	Unc80	0.912872
		54670	Atp8b1	1.002619
		17364	Trpm1	1.029013
		252972	Tpcn1	1.060119
		11931	Atp1b1	1.177377
		241118	Asic4	1.193495
		27219	Sgk2	1.31348
		404545	Ano7	1.334749
		69047	Atp2c2	1.358197
		68667	Trpm4	1.406567
		53313	Atp2a3	1.750981
		225997	Trpm6	1.834091
		12724	Clcn2	2.092737
		14395	Gabra2	2.629172
	Ion transport by P-type ATPases	11937	Atp2a1	-1.7412
		27222	Atp1a4	-1.09805
		54667	Atp8b2	-1.03066
		17178	Fxyd3	-0.93915
		11982	Atp10a	-0.90694
		11933	Atp1b3	-0.76755
		56188	Fxyd1	-0.69905
		67972	Atp2b1	0.66991
		76295	Atp11b	0.76297
		11928	Atp1a1	0.814248
		54670	Atp8b1	1.002619
		11931	Atp1b1	1.177377
		69047	Atp2c2	1.358197
		53313	Atp2a3	1.750981
	Organic cation transport	30805	Slc22a4	-1.19745
		56517	Slc22a21	0.635002
		20520	Slc22a5	0.676375
		18400	Slc22a18	1.470949
		20517	Slc22a1	1.679911
	Transmembrane transport of small molecules	320718	Slc26a9	-3.26554
		11828	Aqp3	-2.8076
		140494	Atp6v0a4	-2.39619

		75750	Slc10a6	-2.13402
		11937	Atp2a1	-1.7412
		15368	Hmox1	-1.6416
		80879	Slc16a3	-1.57048
		14411	Slc6a12	-1.46674
		12870	Cp	-1.32586
		230810	Slc30a2	-1.29338
		213053	Slc39a14	-1.29001
		30805	Slc22a4	-1.19745
		20928	Abcc9	-1.18331
		20190	Ryr1	-1.18304
		22068	Trpc6	-1.17226
		104110	Adcy4	-1.16978
		27222	Atp1a4	-1.09805
		239273	Abcc4	-1.08389
		18173	Slc11a1	-1.04758
		54667	Atp8b2	-1.03066
		210044	Adcy2	-0.99756
		242607	Slc1a7	-0.97491
		63873	Trpv4	-0.95688
		64008	Aqp9	-0.95331
		227059	Slc39a10	-0.94369
		17178	Fxyd3	-0.93915
		20498	Slc12a4	-0.92154
		11982	Atp10a	-0.90694
		11307	Abcg1	-0.88402
		106957	Slc39a6	-0.83374
		330836	Slc7a6	-0.78741
		20527	Slc2a3	-0.77539
		11933	Atp1b3	-0.76755
		353169	Slc2a12	-0.75386
		105722	Ano6	-0.75203
		212032	Hk3	-0.73564
		14709	Gng8	-0.72016
		13340	Slc29a2	-0.71226
		56188	Fxyd1	-0.69905
		14605	Tsc22d3	-0.69762
		14708	Gng7	-0.64044
		100561	Slc15a4	0.58711
		20276	Scnn1a	0.599167
		12725	Clcn3	0.609869

		56517	Slc22a21	0.635002
		67972	Atp2b1	0.66991
		20520	Slc22a5	0.676375
		338370	Nalcn	0.743838
		110893	Slc8a3	0.744391
		21871	Atp6v0a2	0.760918
		20537	Slc5a1	0.762464
		76295	Atp11b	0.76297
		102566	Ano10	0.769236
		69847	Wnk4	0.805242
		11928	Atp1a1	0.814248
		208890	Slc26a7	0.833579
		20499	Slc12a7	0.880019
		71345	Ano9	0.888618
		26357	Abcg2	0.910726
		329178	Unc80	0.912872
		18669	Abcb1b	0.947346
		11512	Adcy6	0.967657
		224814	Abcc10	0.996532
		54670	Atp8b1	1.002619
		218756	Slc4a7	1.018249
		17364	Trpm1	1.029013
		229782	Slc35a3	1.036432
		233836	Slc5a11	1.052542
		53881	Slc5a3	1.053722
		252972	Tpcn1	1.060119
		235504	Slc17a5	1.067807
		27376	Slc25a10	1.077187
		213603	Slc44a3	1.11154
		24060	Slc35a1	1.142999
		11931	Atp1b1	1.177377
		76408	Abcc3	1.189916
		241118	Asic4	1.193495
		15203	Heph	1.252356
		27219	Sgk2	1.31348
		404545	Ano7	1.334749
		12000	Avpr2	1.346946
		69047	Atp2c2	1.358197
		72027	Slc39a4	1.359697
		18671	Abcb1a	1.373398
		68667	Trpm4	1.406567

		18400	Slc22a18	1.470949
		30962	Slc7a9	1.507333
		66333	Aqp11	1.509709
		27421	Abcc6	1.537954
		20517	Slc22a1	1.679911
		110877	Slc18a1	1.691706
		53313	Atp2a3	1.750981
		53945	Slc40a1	1.796217
		225997	Trpm6	1.834091
		13521	Slc26a2	1.912715
		12638	Cftr	1.952521
		28254	Slco1a6	1.98067
		70129	Slc44a4	2.016854
		12724	Clcn2	2.092737
		216225	Slc5a8	2.146749
		72002	Slc39a5	2.180762
		55961	Slc13a1	2.29241
		13487	Slc26a3	2.367692
		56774	Slc6a14	2.523776
		14395	Gabra2	2.629172
		105243	Slc9a3	2.762093
		226999	Slc9a2	2.868787
		20532	Slc3a1	3.223735
		11833	Aqp8	3.844864
		20500	Slc13a2	3.892954

Supplementary Table 3: Details results of ORA after 14 days of exposure of E171 in BALB/c mice in combination with AOM/DSS. Numbers in bold correspond to upregulated genes. Log2FC= Log2 fold change obtained with LIMMA script with correction for its own time-matched control.

Group of pathways	Pathways	EntrezGeneID	Genes	Log2FC
Signalling	Olfactory transduction	258811	Olfr926	-4.64876
		258358	Olfr557	-3.78887
		259072	Olfr686	-3.21602
		258800	Olfr905	-2.8355
		258989	Olfr457	-2.76885
		258836	Olfr272	-2.73318
		258729	Olfr478	-2.64268
		258320	Olfr1232	-2.62691
		258621	Olfr344	-2.57238
		258371	Olfr368	-2.41772
		258775	Olfr196	-2.4027
		258177	Olfr1222	-2.34283
		258429	Olfr996	-2.2195
		258577	Olfr1026	-2.18809
		227789	Olfr358	-2.16778
		257912	Olfr948	-2.16021
		404222	Olfr231	-2.03002
		258982	Olfr1272	-2.01257
		258674	Olfr1427	-1.9539
		258315	Olfr767	-1.94937
		259071	Olfr166	-1.91143
		18332	Olfr33	-1.90223
		258025	Olfr1211	-1.8169
		258734	Olfr502	-1.70538
		258807	Olfr910	-1.69483
		170639	Olfr78	-1.69158
		258649	Olfr441	-1.68312
		258880	Olfr218	-1.66686
		258563	Olfr1006	-1.61089
		258559	Olfr830	-1.58115
		258350	Olfr706	-1.56576
		258939	Olfr63	-1.53883
		18344	Olfr45	-1.52067
		258910	Olfr1280	-1.52065

		258560	Olfr843	-1.51915
		258744	Olfr875	-1.49692
		18365	Olfr65	-1.48665
		258273	Olfr1394	-1.48421
		258465	Olfr1387	-1.39693
		258737	Olfr1316	-1.36997
		258343	Olfr397	-1.36572
		258629	Olfr1487	-1.34557
		258932	Olfr791	-1.3421
		258173	Olfr1532-ps1	-1.3396
		258785	Olfr1230	-1.32246
		258962	Olfr53	-1.3223
		259033	Olfr1122	-1.3054
		258028	Olfr901	-1.2159
		18346	Olfr47	-1.18329
		258511	Olfr523	-1.17449
		258334	Olfr1396	-1.15271
		258525	Olfr1170	-1.14879
		259041	Olfr1414	-1.14487
		258704	Olfr411	-1.13571
		233649	Cnga4	-1.13302
		258417	Olfr470	-1.13038
		545985	Olfr612	-1.12476
		259116	Olfr559	-1.10927
		258808	Olfr620	-1.1068
		258705	Olfr398	-1.09713
		258281	Olfr780	-1.09618
		258791	Olfr812	-1.08902
		258055	Olfr524	-1.01384
		258271	Olfr1311	-1.0065
		258151	Olfr1505	-0.97509
		258991	Olfr1496	-0.94303
		107477	Guca1b	-0.85852
		259113	Olfr577	-0.80041
		258718	Olfr513	-0.77543
		258505	Olfr97	-0.74544
		258782	Olfr914	-0.74005
		258290	Olfr1143	-0.71076
		258198	Olfr224	-0.70859
		18749	Prkacb	-0.69222
		57269	Olfr1507	-0.68481

		258949	Olfr338	-0.68418
		258615	Olfr360	-0.67571
		**258552**	**Olfr868**	**0.63894**
		**56860**	**Olfr690**	**0.643344**
		**383243**	**Olfr128**	**0.686736**
		**258463**	**Olfr1393**	**0.6995**
		**258801**	**Olfr907**	**0.701786**
		**258652**	**Olfr1131**	**0.710637**
		**259119**	**Olfr578**	**0.71483**
		**258728**	**Olfr482**	**0.7194**
		**18322**	**Olfr24**	**0.762628**
		**110511**	**Olfr153**	**0.805912**
		**258248**	**Olfr576**	**0.852716**
		**258487**	**Olfr722**	**0.875545**
		**259053**	**Olfr362**	**0.886359**
		**57271**	**Olfr1509**	**0.887**
		**258269**	**Olfr930**	**0.892154**
		**258224**	**Olfr1358**	**0.900034**
		**18331**	**Olfr32**	**0.906447**
		**258392**	**Olfr190**	**0.908549**
		**258512**	**Olfr530**	**0.917809**
		**258913**	**Olfr1377**	**0.930402**
		**258499**	**Olfr945**	**0.933335**
		**258625**	**Olfr116**	**0.941665**
		**258825**	**Olfr975**	**0.944944**
		**257959**	**Olfr1537**	**0.981626**
		**258518**	**Olfr847**	**0.99196**
		**233670**	**Olfr6**	**1.010129**
		**259000**	**Olfr195**	**1.024429**
		**258492**	**Olfr484**	**1.030595**
		**257932**	**Olfr332**	**1.038775**
		**258201**	**Olfr538**	**1.081171**
		**258544**	**Olfr788**	**1.082466**
		**634104**	**Olfr287**	**1.082896**
		**258665**	**Olfr815**	**1.09629**
		**258507**	**Olfr96**	**1.112944**
		**258882**	**Olfr874**	**1.141852**
		**258602**	**Olfr150**	**1.145491**
		**258722**	**Olfr611**	**1.165157**
		**259006**	**Olfr399**	**1.169014**
		**258984**	**Olfr1257**	**1.187363**

		**259064**	**Olfr124**	**1.197695**
		**257909**	**Olfr735**	**1.237712**
		**258799**	**Olfr906**	**1.243894**
		**259114**	**Olfr570**	**1.248525**
		**258877**	**Olfr1395**	**1.255895**
		**258751**	**Olfr608**	**1.280827**
		**117005**	**Olfr74**	**1.29757**
		**258694**	**Olfr1445**	**1.302618**
		**258851**	**Olfr1339**	**1.339229**
		**257890**	**Olfr12**	**1.339785**
		**258661**	**Olfr740**	**1.341121**
		**258952**	**Olfr341**	**1.377262**
		**258354**	**Olfr168**	**1.388465**
		**257978**	**Olfr1322**	**1.404626**
		**258716**	**Olfr424**	**1.435701**
		**258282**	**Olfr801**	**1.467309**
		**258606**	**Olfr229**	**1.518727**
		**258852**	**Olfr1341**	**1.520375**
		**259124**	**Olfr638**	**1.520383**
		**258739**	**Olfr1362**	**1.612199**
		**258969**	**Olfr1226**	**1.619691**
		**384732**	**Gm10081**	**1.732919**
		**258319**	**Olfr187**	**1.734293**
		**18347**	**Olfr48**	**1.776151**
		**258638**	**Olfr1163**	**1.786424**
		**258903**	**Olfr1217**	**1.838686**
		**258959**	**Olfr170**	**1.949262**
		**258285**	**Olfr122**	**2.125295**
		**258928**	**Olfr477**	**2.144292**
		**259025**	**Olfr385**	**2.234689**
		**258859**	**Olfr161**	**3.109598**
		**258762**	**Olfr1109**	**3.197569**
		**19092**	**Prkg2**	**3.495995**
	GPCR downstream signalling	258811	Olfr926	-4.64876
		23919	Insl5	-4.39738
		258358	Olfr557	-3.78887
		26361	Avpr1b	-2.92801
		258836	Olfr272	-2.73318
		258729	Olfr478	-2.64268
		258320	Olfr1232	-2.62691
		258371	Olfr368	-2.41772

		258775	Olfr196	-2.4027
		14652	Glp1r	-2.36498
		258177	Olfr1222	-2.34283
		17202	Mc4r	-2.32772
		19064	Ppy	-2.23412
		217212	Pyy	-2.12279
		387347	Tas2r118	-2.1135
		404222	Olfr231	-2.03002
		258982	Olfr1272	-2.01257
		258674	Olfr1427	-1.9539
		258315	Olfr767	-1.94937
		259071	Olfr166	-1.91143
		258025	Olfr1211	-1.8169
		14459	Gast	-1.80179
		257880	Olfr1066	-1.78941
		574417	Tas2r137	-1.7196
		258734	Olfr502	-1.70538
		170639	Olfr78	-1.69158
		258649	Olfr441	-1.68312
		258880	Olfr218	-1.66686
		667992	Tas2r103	-1.64983
		258563	Olfr1006	-1.61089
		17773	Mtnr1a	-1.58287
		258559	Olfr830	-1.58115
		258350	Olfr706	-1.56576
		258939	Olfr63	-1.53883
		258560	Olfr843	-1.51915
		258744	Olfr875	-1.49692
		18365	Olfr65	-1.48665
		258465	Olfr1387	-1.39693
		16867	Lhcgr	-1.37102
		258932	Olfr791	-1.3421
		268934	Grm4	-1.33684
		258785	Olfr1230	-1.32246
		259033	Olfr1122	-1.3054
		18386	Oprd1	-1.27226
		58991	Ghrl	-1.26899
		15551	Htr1b	-1.2463
		16952	Anxa1	-1.18898
		18346	Olfr47	-1.18329
		14309	Fshr	-1.17472

		22068	Trpc6	-1.15821
		258525	Olfr1170	-1.14879
		259041	Olfr1414	-1.14487
		15566	Htr7	-1.1364
		258704	Olfr411	-1.13571
		258417	Olfr470	-1.13038
		21336	Tacr1	-1.12491
		258180	Olfr699	-1.12295
		258808	Olfr620	-1.1068
		258281	Olfr780	-1.09618
		14675	Gna14	-1.07872
		18751	Prkcb	-1.07462
		99296	Hrh3	-1.05305
		242093	Rxfp4	-1.04311
		15562	Htr4	-1.03209
		18166	Npy1r	-1.02075
		12772	Ccr2	-1.00727
		12773	Ccr4	-1.00322
		13614	*Edn1*	*-0.99296*
		19204	Ptafr	-0.97951
		258151	Olfr1505	-0.97509
		21333	Tac1	-0.97278
		24012	Rgs7	-0.96881
		258064	Olfr316	-0.95922
		258991	Olfr1496	-0.94303
		11555	Adrb2	-0.92768
		18796	Plcb2	-0.92128
		227289	Gpbar1	-0.88141
		19227	Pthlh	-0.88025
		12776	Ccr8	-0.85933
		18583	Pde7a	-0.84692
		11606	Agt	-0.80285
		259113	Olfr577	-0.80041
		217480	Dgkb	-0.80037
		56470	Rgs19	-0.78909
		11513	Adcy7	-0.76956
		18829	Ccl21a	-0.76839
		244701	Mtnr1b	-0.7353
		22324	Vav1	-0.73052
		258290	Olfr1143	-0.71076
		73341	Arhgef6	-0.68006

		319197	Gpr4	-0.63098
		53978	Lpar2	-0.61346
		14062	F2r	-0.61231
		19219	*Ptger4*	*-0.59249*
		18573	Pde1a	-0.58797
		16801	Arhgef1	-0.58553
		233571	P2ry6	-0.58524
		**69632**	**Arhgef12**	**0.585643**
		**258552**	**Olfr868**	**0.63894**
		**258463**	**Olfr1393**	**0.6995**
		**258652**	**Olfr1131**	**0.710637**
		**259119**	**Olfr578**	**0.71483**
		**258728**	**Olfr482**	**0.7194**
		**387343**	**Tas2r109**	**0.735099**
		**12921**	**Crhr1**	**0.757531**
		**18441**	**P2ry1**	**0.758201**
		**18322**	**Olfr24**	**0.762628**
		**320127**	**Dgki**	**0.800134**
		**110511**	**Olfr153**	**0.805912**
		**65086**	**Lpar3**	**0.832089**
		**12672**	**Chrm4**	**0.850005**
		**11517**	**Adcyap1r1**	**0.851143**
		**321020**	**Fpr-rs6**	**0.856069**
		**13491**	**Drd4**	**0.875113**
		**258487**	**Olfr722**	**0.875545**
		**259053**	**Olfr362**	**0.886359**
		**57271**	**Olfr1509**	**0.887**
		**258269**	**Olfr930**	**0.892154**
		**18331**	**Olfr32**	**0.906447**
		**14294**	**Fpr3**	**0.944948**
		**14676**	**Gna15**	**0.956512**
		**387356**	**Tas2r131**	**0.958132**
		**13492**	**Drd5**	**0.966222**
		**258518**	**Olfr847**	**0.99196**
		**259000**	**Olfr195**	**1.024429**
		**258492**	**Olfr484**	**1.030595**
		**258544**	**Olfr788**	**1.082466**
		**634104**	**Olfr287**	**1.082896**
		**258665**	**Olfr815**	**1.09629**
		**258507**	**Olfr96**	**1.112944**
		**258882**	**Olfr874**	**1.141852**

		**258984**	**Olfr1257**	**1.187363**
		**15552**	**Htr1d**	**1.221482**
		**257909**	**Olfr735**	**1.237712**
		**12000**	**Avpr2**	**1.251924**
		**117005**	**Olfr74**	**1.29757**
		**258694**	**Olfr1445**	**1.302618**
		**20300**	**Ccl25**	**1.322852**
		**57385**	**P2ry4**	**1.327103**
		**258661**	**Olfr740**	**1.341121**
		**18167**	**Npy2r**	**1.341246**
		**22044**	**Trh**	**1.380194**
		**258354**	**Olfr168**	**1.388465**
		**258716**	**Olfr424**	**1.435701**
		**26946**	**Trpc7**	**1.470884**
		**259124**	**Olfr638**	**1.520383**
		**258969**	**Olfr1226**	**1.619691**
		**384732**	**Gm10081**	**1.732919**
		**258319**	**Olfr187**	**1.734293**
		**258638**	**Olfr1163**	**1.786424**
		**11541**	**Adora2b**	**1.805445**
		**258903**	**Olfr1217**	**1.838686**
		**258959**	**Olfr170**	**1.949262**
		**116903**	**Calcb**	**2.19162**
		**259025**	**Olfr385**	**2.234689**
		**67405**	**Nts**	**3.057371**
		**258859**	**Olfr161**	**3.109598**
	Signalling by GPCR	258811	Olfr926	-4.64876
		23919	Insl5	-4.39738
		258358	Olfr557	-3.78887
		26361	Avpr1b	-2.92801
		258836	Olfr272	-2.73318
		258729	Olfr478	-2.64268
		258320	Olfr1232	-2.62691
		22413	Wnt2	-2.58182
		258371	Olfr368	-2.41772
		258775	Olfr196	-2.4027
		14652	Glp1r	-2.36498
		258177	Olfr1222	-2.34283
		17202	Mc4r	-2.32772
		19064	Ppy	-2.23412
		217212	Pyy	-2.12279

			387347	Tas2r118	-2.1135
			404222	Olfr231	-2.03002
			258982	Olfr1272	-2.01257
			258674	Olfr1427	-1.9539
			258315	Olfr767	-1.94937
			259071	Olfr166	-1.91143
			83770	Tas1r2	-1.84156
			258025	Olfr1211	-1.8169
			14459	Gast	-1.80179
			257880	Olfr1066	-1.78941
			574417	Tas2r137	-1.7196
			258734	Olfr502	-1.70538
			170639	Olfr78	-1.69158
			258649	Olfr441	-1.68312
			258880	Olfr218	-1.66686
			667992	Tas2r103	-1.64983
			258563	Olfr1006	-1.61089
			17773	Mtnr1a	-1.58287
			258559	Olfr830	-1.58115
			258350	Olfr706	-1.56576
			258939	Olfr63	-1.53883
			258560	Olfr843	-1.51915
			13137	Daf2	-1.49756
			258744	Olfr875	-1.49692
			18365	Olfr65	-1.48665
			258465	Olfr1387	-1.39693
			16867	Lhcgr	-1.37102
			258932	Olfr791	-1.3421
			268934	Grm4	-1.33684
			258785	Olfr1230	-1.32246
			259033	Olfr1122	-1.3054
			13136	Cd55	-1.29775
			18386	Oprd1	-1.27226
			58991	Ghrl	-1.26899
			15551	Htr1b	-1.2463
			16952	Anxa1	-1.18898
			18346	Olfr47	-1.18329
			14309	Fshr	-1.17472
			22068	Trpc6	-1.15821
			258525	Olfr1170	-1.14879
			259041	Olfr1414	-1.14487

		15566	Htr7	-1.1364
		258704	Olfr411	-1.13571
		258417	Olfr470	-1.13038
		21336	Tacr1	-1.12491
		258180	Olfr699	-1.12295
		258808	Olfr620	-1.1068
		258281	Olfr780	-1.09618
		14675	Gna14	-1.07872
		18751	Prkcb	-1.07462
		99296	Hrh3	-1.05305
		242093	Rxfp4	-1.04311
		15562	Htr4	-1.03209
		18166	Npy1r	-1.02075
		12772	Ccr2	-1.00727
		12773	Ccr4	-1.00322
		13614	*Edn1*	*-0.99296*
		19204	Ptafr	-0.97951
		258151	Olfr1505	-0.97509
		21333	Tac1	-0.97278
		24012	Rgs7	-0.96881
		12326	Camk4	-0.96196
		258064	Olfr316	-0.95922
		258991	Olfr1496	-0.94303
		11555	Adrb2	-0.92768
		18796	Plcb2	-0.92128
		227289	Gpbar1	-0.88141
		19227	Pthlh	-0.88025
		12776	Ccr8	-0.85933
		18583	Pde7a	-0.84692
		11606	Agt	-0.80285
		259113	Olfr577	-0.80041
		217480	Dgkb	-0.80037
		56470	Rgs19	-0.78909
		11513	Adcy7	-0.76956
		18829	Ccl21a	-0.76839
		244701	Mtnr1b	-0.7353
		22324	Vav1	-0.73052
		14710	Gngt2	-0.72984
		258290	Olfr1143	-0.71076
		18749	Prkacb	-0.69222
		73341	Arhgef6	-0.68006

		14784	Grb2	-0.6724
		319197	Gpr4	-0.63098
		53978	Lpar2	-0.61346
		14062	F2r	-0.61231
		26364	Adgre5	-0.60643
		19219	*Ptger4*	*-0.59249*
		18573	Pde1a	-0.58797
		16801	Arhgef1	-0.58553
		233571	P2ry6	-0.58524
		**69632**	**Arhgef12**	**0.585643**
		**83771**	**Tas1r3**	**0.629121**
		**14370**	**Fzd8**	**0.631495**
		**258552**	**Olfr868**	**0.63894**
		**258463**	**Olfr1393**	**0.6995**
		**258652**	**Olfr1131**	**0.710637**
		**259119**	**Olfr578**	**0.71483**
		**258728**	**Olfr482**	**0.7194**
		**387343**	**Tas2r109**	**0.735099**
		**12921**	**Crhr1**	**0.757531**
		**18441**	**P2ry1**	**0.758201**
		**18322**	**Olfr24**	**0.762628**
		**320127**	**Dgki**	**0.800134**
		**110511**	**Olfr153**	**0.805912**
		**65086**	**Lpar3**	**0.832089**
		**12672**	**Chrm4**	**0.850005**
		**11517**	**Adcyap1r1**	**0.851143**
		**321020**	**Fpr-rs6**	**0.856069**
		**13491**	**Drd4**	**0.875113**
		**258487**	**Olfr722**	**0.875545**
		**259053**	**Olfr362**	**0.886359**
		**57271**	**Olfr1509**	**0.887**
		**258269**	**Olfr930**	**0.892154**
		**18331**	**Olfr32**	**0.906447**
		**14294**	**Fpr3**	**0.944948**
		**14676**	**Gna15**	**0.956512**
		**387356**	**Tas2r131**	**0.958132**
		**13492**	**Drd5**	**0.966222**
		**258518**	**Olfr847**	**0.99196**
		**259000**	**Olfr195**	**1.024429**
		**258492**	**Olfr484**	**1.030595**
		**258544**	**Olfr788**	**1.082466**

		**634104**	**Olfr287**	**1.082896**
		**258665**	**Olfr815**	**1.09629**
		**258507**	**Olfr96**	**1.112944**
		**258882**	**Olfr874**	**1.141852**
		**258984**	**Olfr1257**	**1.187363**
		**15552**	**Htr1d**	**1.221482**
		**257909**	**Olfr735**	**1.237712**
		**12000**	**Avpr2**	**1.251924**
		**117005**	**Olfr74**	**1.29757**
		**258694**	**Olfr1445**	**1.302618**
		**20300**	**Ccl25**	**1.322852**
		**57385**	**P2ry4**	**1.327103**
		**258661**	**Olfr740**	**1.341121**
		**18167**	**Npy2r**	**1.341246**
		**22044**	**Trh**	**1.380194**
		**258354**	**Olfr168**	**1.388465**
		**258716**	**Olfr424**	**1.435701**
		**252837**	**Ackr4**	**1.449683**
		**26946**	**Trpc7**	**1.470884**
		**259124**	**Olfr638**	**1.520383**
		**258969**	**Olfr1226**	**1.619691**
		**384732**	**Gm10081**	**1.732919**
		**258319**	**Olfr187**	**1.734293**
		**258638**	**Olfr1163**	**1.786424**
		**11541**	**Adora2b**	**1.805445**
		**258903**	**Olfr1217**	**1.838686**
		**258959**	**Olfr170**	**1.949262**
		**116903**	**Calcb**	**2.19162**
		**259025**	**Olfr385**	**2.234689**
		**67405**	**Nts**	**3.057371**
		**258859**	**Olfr161**	**3.109598**
	Olfactory signalling Pathway	258811	Olfr926	-4.64876
		258358	Olfr557	-3.78887
		258836	Olfr272	-2.73318
		258729	Olfr478	-2.64268
		258320	Olfr1232	-2.62691
		258371	Olfr368	-2.41772
		258775	Olfr196	-2.4027
		258177	Olfr1222	-2.34283
		404222	Olfr231	-2.03002
		258982	Olfr1272	-2.01257

		258674	Olfr1427	-1.9539
		258315	Olfr767	-1.94937
		259071	Olfr166	-1.91143
		258025	Olfr1211	-1.8169
		257880	Olfr1066	-1.78941
		258734	Olfr502	-1.70538
		170639	Olfr78	-1.69158
		258649	Olfr441	-1.68312
		258880	Olfr218	-1.66686
		258563	Olfr1006	-1.61089
		258559	Olfr830	-1.58115
		258350	Olfr706	-1.56576
		258939	Olfr63	-1.53883
		258560	Olfr843	-1.51915
		258744	Olfr875	-1.49692
		18365	Olfr65	-1.48665
		258465	Olfr1387	-1.39693
		258932	Olfr791	-1.3421
		258785	Olfr1230	-1.32246
		259033	Olfr1122	-1.3054
		18346	Olfr47	-1.18329
		258525	Olfr1170	-1.14879
		259041	Olfr1414	-1.14487
		258704	Olfr411	-1.13571
		258417	Olfr470	-1.13038
		258180	Olfr699	-1.12295
		258808	Olfr620	-1.1068
		258281	Olfr780	-1.09618
		258151	Olfr1505	-0.97509
		258064	Olfr316	-0.95922
		258991	Olfr1496	-0.94303
		259113	Olfr577	-0.80041
		258290	Olfr1143	-0.71076
		**258552**	**Olfr868**	**0.63894**
		**258463**	**Olfr1393**	**0.6995**
		**258652**	**Olfr1131**	**0.710637**
		**259119**	**Olfr578**	**0.71483**
		**258728**	**Olfr482**	**0.7194**
		**18322**	**Olfr24**	**0.762628**
		**110511**	**Olfr153**	**0.805912**
		**258487**	**Olfr722**	**0.875545**

		**259053**	**Olfr362**	**0.886359**
		**57271**	**Olfr1509**	**0.887**
		**258269**	**Olfr930**	**0.892154**
		**18331**	**Olfr32**	**0.906447**
		**258518**	**Olfr847**	**0.99196**
		**259000**	**Olfr195**	**1.024429**
		**258492**	**Olfr484**	**1.030595**
		**258544**	**Olfr788**	**1.082466**
		**634104**	**Olfr287**	**1.082896**
		**258665**	**Olfr815**	**1.09629**
		**258507**	**Olfr96**	**1.112944**
		**258882**	**Olfr874**	**1.141852**
		**258984**	**Olfr1257**	**1.187363**
		**257909**	**Olfr735**	**1.237712**
		**117005**	**Olfr74**	**1.29757**
		**258694**	**Olfr1445**	**1.302618**
		**258661**	**Olfr740**	**1.341121**
		**258354**	**Olfr168**	**1.388465**
		**258716**	**Olfr424**	**1.435701**
		**259124**	**Olfr638**	**1.520383**
		**258969**	**Olfr1226**	**1.619691**
		**384732**	**Gm10081**	**1.732919**
		**258319**	**Olfr187**	**1.734293**
		**258638**	**Olfr1163**	**1.786424**
		**258903**	**Olfr1217**	**1.838686**
		**258959**	**Olfr170**	**1.949262**
		**259025**	**Olfr385**	**2.234689**
		**258859**	**Olfr161**	**3.109598**
	Signal Transduction	258811	Olfr926	-4.64876
		23919	Insl5	-4.39738
		258358	Olfr557	-3.78887
		21828	Thbs4	-3.44354
		13386	Dlk1	-3.27428
		26361	Avpr1b	-2.92801
		258836	Olfr272	-2.73318
		258729	Olfr478	-2.64268
		258320	Olfr1232	-2.62691
		22413	Wnt2	-2.58182
		258371	Olfr368	-2.41772
		258775	Olfr196	-2.4027
		14652	Glp1r	-2.36498

		258177	Olfr1222	-2.34283
		17202	Mc4r	-2.32772
		22139	Ttr	-2.27389
		19064	Ppy	-2.23412
		242341	Atp6v0d2	-2.21608
		217212	Pyy	-2.12279
		387347	Tas2r118	-2.1135
		338375	Atp6v1g3	-2.04699
		404222	Olfr231	-2.03002
		258982	Olfr1272	-2.01257
		245650	Gucy2f	-1.96801
		258674	Olfr1427	-1.9539
		258315	Olfr767	-1.94937
		259071	Olfr166	-1.91143
		83770	Tas1r2	-1.84156
		258025	Olfr1211	-1.8169
		14459	Gast	-1.80179
		257880	Olfr1066	-1.78941
		17228	Cma1	-1.73523
		83379	Klb	-1.72259
		574417	Tas2r137	-1.7196
		258734	Olfr502	-1.70538
		170639	Olfr78	-1.69158
		258649	Olfr441	-1.68312
		258880	Olfr218	-1.66686
		14725	Lrp2	-1.65004
		667992	Tas2r103	-1.64983
		16323	Inhba	-1.62741
		258563	Olfr1006	-1.61089
		17773	Mtnr1a	-1.58287
		258559	Olfr830	-1.58115
		258350	Olfr706	-1.56576
		258939	Olfr63	-1.53883
		76117	Arhgap15	-1.53779
		258560	Olfr843	-1.51915
		13137	Daf2	-1.49756
		258744	Olfr875	-1.49692
		18365	Olfr65	-1.48665
		54613	St3gal6	-1.44575
		232201	Arhgap25	-1.4054
		14357	Dtx1	-1.40444

		258465	Olfr1387	-1.39693
		16867	Lhcgr	-1.37102
		258932	Olfr791	-1.3421
		268934	Grm4	-1.33684
		258785	Olfr1230	-1.32246
		259033	Olfr1122	-1.3054
		13136	Cd55	-1.29775
		18386	Oprd1	-1.27226
		58991	Ghrl	-1.26899
		15551	Htr1b	-1.2463
		16952	Anxa1	-1.18898
		18346	Olfr47	-1.18329
		14309	Fshr	-1.17472
		14268	Fn1	-1.16448
		22068	Trpc6	-1.15821
		258525	Olfr1170	-1.14879
		259041	Olfr1414	-1.14487
		15566	Htr7	-1.1364
		258704	Olfr411	-1.13571
		258417	Olfr470	-1.13038
		21336	Tacr1	-1.12491
		258180	Olfr699	-1.12295
		258808	Olfr620	-1.1068
		258281	Olfr780	-1.09618
		14675	Gna14	-1.07872
		18751	Prkcb	-1.07462
		99296	Hrh3	-1.05305
		242093	Rxfp4	-1.04311
		22341	Vegfc	-1.03365
		15562	Htr4	-1.03209
		18166	Npy1r	-1.02075
		12772	Ccr2	-1.00727
		18815	Plg	-1.00547
		12773	Ccr4	-1.00322
		13614	*Edn1*	*-0.99296*
		19204	Ptafr	-0.97951
		258151	Olfr1505	-0.97509
		21333	Tac1	-0.97278
		11857	Arhgdib	-0.969
		24012	Rgs7	-0.96881
		12326	Camk4	-0.96196

		258064	Olfr316	-0.95922
		258991	Olfr1496	-0.94303
		11555	Adrb2	-0.92768
		14735	Gpc4	-0.92575
		18796	Plcb2	-0.92128
		80837	Rhoj	-0.9051
		15205	Hes1	-0.88304
		227289	Gpbar1	-0.88141
		19227	Pthlh	-0.88025
		94212	Pag1	-0.86399
		12776	Ccr8	-0.85933
		107477	Guca1b	-0.85852
		72536	Tagap	-0.85136
		12487	Cd28	-0.85003
		18583	Pde7a	-0.84692
		18776	Prl3b1	-0.8162
		11606	Agt	-0.80285
		259113	Olfr577	-0.80041
		217480	Dgkb	-0.80037
		56470	Rgs19	-0.78909
		11513	Adcy7	-0.76956
		18829	Ccl21a	-0.76839
		20441	St3gal3	-0.74711
		17136	Mag	-0.74259
		18775	Prl3d1	-0.74072
		244701	Mtnr1b	-0.7353
		22324	Vav1	-0.73052
		14710	Gngt2	-0.72984
		14257	Flt4	-0.71094
		258290	Olfr1143	-0.71076
		18749	Prkacb	-0.69222
		58194	Sh3kbp1	-0.68653
		73341	Arhgef6	-0.68006
		14784	Grb2	-0.6724
		17242	Mdk	-0.6707
		17921	Myo7a	-0.64451
		319197	Gpr4	-0.63098
		83554	Fstl3	-0.6248
		53978	Lpar2	-0.61346
		14062	F2r	-0.61231
		21894	Tln1	-0.60783

		26364	Adgre5	-0.60643
		54601	Foxo4	-0.60052
		16367	Irs1	-0.59977
		19219	*Ptger4*	*-0.59249*
		18573	Pde1a	-0.58797
		16801	Arhgef1	-0.58553
		233571	P2ry6	-0.58524
		**69632**	**Arhgef12**	**0.585643**
		**17311**	**Kitl**	**0.607134**
		**20602**	**Ncor2**	**0.620732**
		**83771**	**Tas1r3**	**0.629121**
		**14370**	**Fzd8**	**0.631495**
		**16971**	**Lrp1**	**0.638471**
		**258552**	**Olfr868**	**0.63894**
		**258463**	**Olfr1393**	**0.6995**
		**258652**	**Olfr1131**	**0.710637**
		**259119**	**Olfr578**	**0.71483**
		**258728**	**Olfr482**	**0.7194**
		**387343**	**Tas2r109**	**0.735099**
		**230597**	**Zfyve9**	**0.74451**
		**12921**	**Crhr1**	**0.757531**
		**18441**	**P2ry1**	**0.758201**
		**18322**	**Olfr24**	**0.762628**
		**19111**	**Prl6a1**	**0.770609**
		**320127**	**Dgki**	**0.800134**
		**110511**	**Olfr153**	**0.805912**
		**65086**	**Lpar3**	**0.832089**
		**259302**	**Srgap3**	**0.835414**
		**12672**	**Chrm4**	**0.850005**
		**11517**	**Adcyap1r1**	**0.851143**
		**108099**	**Prkag2**	**0.85238**
		**321020**	**Fpr-rs6**	**0.856069**
		**13491**	**Drd4**	**0.875113**
		**258487**	**Olfr722**	**0.875545**
		**259053**	**Olfr362**	**0.886359**
		**57271**	**Olfr1509**	**0.887**
		**258269**	**Olfr930**	**0.892154**
		**20404**	**Sh3gl2**	**0.903947**
		**18331**	**Olfr32**	**0.906447**
		**14294**	**Fpr3**	**0.944948**
		**56484**	**Foxo3**	**0.947986**

		14676	Gna15	0.956512
		387356	Tas2r131	0.958132
		13492	Drd5	0.966222
		19273	Ptpru	0.976005
		74915	Atp6v1e2	0.979016
		258518	Olfr847	0.99196
		259000	Olfr195	1.024429
		258492	Olfr484	1.030595
		14599	Gh	1.043182
		69993	Chn2	1.070246
		258544	Olfr788	1.082466
		634104	Olfr287	1.082896
		258665	Olfr815	1.09629
		22414	Wnt2b	1.109751
		258507	Olfr96	1.112944
		258882	Olfr874	1.141852
		258984	Olfr1257	1.187363
		15552	Htr1d	1.221482
		257909	Olfr735	1.237712
		12000	Avpr2	1.251924
		545481	Arhgap40	1.285542
		117005	Olfr74	1.29757
		258694	Olfr1445	1.302618
		20300	Ccl25	1.322852
		57385	P2ry4	1.327103
		258661	Olfr740	1.341121
		18167	Npy2r	1.341246
		22044	Trh	1.380194
		258354	Olfr168	1.388465
		258716	Olfr424	1.435701
		19116	Prlr	1.437521
		252837	Ackr4	1.449683
		26946	Trpc7	1.470884
		259124	Olfr638	1.520383
		258969	Olfr1226	1.619691
		384732	Gm10081	1.732919
		258319	Olfr187	1.734293
		258638	Olfr1163	1.786424
		11541	Adora2b	1.805445
		258903	Olfr1217	1.838686
		258959	Olfr170	1.949262

		**105349**	**Akr1c18**	**1.966455**
		**244867**	**Arhgap20**	**1.969602**
		**116903**	**Calcb**	**2.19162**
		**259025**	**Olfr385**	**2.234689**
		**67405**	**Nts**	**3.057371**
		**258859**	**Olfr161**	**3.109598**
		**238055**	**Apob**	**3.336986**
	Odorant GPCRs	14608	Gpr83	-3.21469
		77596	Adgrf1	-2.76988
		258729	Olfr478	-2.64268
		64378	Gpr88	-2.30643
		381413	Gpr176	-2.13457
		83924	Gpr137b	-2.12778
		209513	Taar4	-1.97343
		113847	Vmn1r43	-1.9454
		113846	Vmn1r47	-1.92418
		83770	Tas1r2	-1.84156
		664862	Gpr137b-ps	-1.82967
		258734	Olfr502	-1.70538
		258939	Olfr63	-1.53883
		209517	Taar7b	-1.36067
		329252	Lgr6	-1.35867
		113848	Vmn1r42	-1.29014
		229357	Gpr149	-1.26846
		258417	Olfr470	-1.13038
		435207	Taar7f	-0.98449
		70693	Adgra2	-0.92974
		239530	Gpr20	-0.90468
		64450	Gpr85	-0.85607
		319197	Gpr4	-0.63098
		224792	Adgrf5	-0.62424
		**83771**	**Tas1r3**	**0.629121**
		**269053**	**Gpr152**	**0.701654**
		**258728**	**Olfr482**	**0.7194**
		**387343**	**Tas2r109**	**0.735099**
		**18322**	**Olfr24**	**0.762628**
		**52710**	**Slc52a2**	**0.805314**
		**76206**	**Gpr165**	**0.849664**
		**107515**	**Lgr4**	**0.851757**
		**233230**	**Mrgprb4**	**0.92276**
		**257959**	**Olfr1537**	**0.981626**

		**233670**	**Olfr6**	**1.010129**
		**258492**	**Olfr484**	**1.030595**
		**215854**	**Taar5**	**1.272795**
		**257890**	**Olfr12**	**1.339785**
		**14761**	**Gpr27**	**1.471809**
		**113850**	**V1ra8**	**1.713639**
		**258319**	**Olfr187**	**1.734293**
		**171469**	**Gpr37l1**	**1.787234**
		**258928**	**Olfr477**	**2.144292**
	GPCR ligand binding	23919	Insl5	-4.39738
		26361	Avpr1b	-2.92801
		22413	Wnt2	-2.58182
		14652	Glp1r	-2.36498
		17202	Mc4r	-2.32772
		19064	Ppy	-2.23412
		217212	Pyy	-2.12279
		387347	Tas2r118	-2.1135
		83770	Tas1r2	-1.84156
		574417	Tas2r137	-1.7196
		667992	Tas2r103	-1.64983
		17773	Mtnr1a	-1.58287
		13137	Daf2	-1.49756
		16867	Lhcgr	-1.37102
		268934	Grm4	-1.33684
		13136	Cd55	-1.29775
		18386	Oprd1	-1.27226
		58991	Ghrl	-1.26899
		15551	Htr1b	-1.2463
		16952	Anxa1	-1.18898
		14309	Fshr	-1.17472
		15566	Htr7	-1.1364
		21336	Tacr1	-1.12491
		99296	Hrh3	-1.05305
		242093	Rxfp4	-1.04311
		15562	Htr4	-1.03209
		18166	Npy1r	-1.02075
		12773	Ccr4	-1.00322
		13614	*Edn1*	*-0.99296*
		19204	Ptafr	-0.97951
		21333	Tac1	-0.97278
		11555	Adrb2	-0.92768

		227289	Gpbar1	-0.88141
		19227	Pthlh	-0.88025
		11606	Agt	-0.80285
		18829	Ccl21a	-0.76839
		244701	Mtnr1b	-0.7353
		14710	Gngt2	-0.72984
		319197	Gpr4	-0.63098
		53978	Lpar2	-0.61346
		14062	F2r	-0.61231
		26364	Adgre5	-0.60643
		19219	*Ptger4*	*-0.59249*
		233571	P2ry6	-0.58524
		**83771**	**Tas1r3**	**0.629121**
		**14370**	**Fzd8**	**0.631495**
		**387343**	**Tas2r109**	**0.735099**
		**12921**	**Crhr1**	**0.757531**
		**18441**	**P2ry1**	**0.758201**
		**65086**	**Lpar3**	**0.832089**
		**12672**	**Chrm4**	**0.850005**
		**11517**	**Adcyap1r1**	**0.851143**
		**321020**	**Fpr-rs6**	**0.856069**
		**13491**	**Drd4**	**0.875113**
		**14294**	**Fpr3**	**0.944948**
		**387356**	**Tas2r131**	**0.958132**
		**13492**	**Drd5**	**0.966222**
		**15552**	**Htr1d**	**1.221482**
		**12000**	**Avpr2**	**1.251924**
		**20300**	**Ccl25**	**1.322852**
		**57385**	**P2ry4**	**1.327103**
		**18167**	**Npy2r**	**1.341246**
		**22044**	**Trh**	**1.380194**
		**252837**	**Ackr4**	**1.449683**
		**11541**	**Adora2b**	**1.805445**
		**116903**	**Calcb**	**2.19162**
		**67405**	**Nts**	**3.057371**
	GPCRs, Class A Rhodopsin-like	258811	Olfr926	-4.64876
		14608	Gpr83	-3.21469
		26361	Avpr1b	-2.92801
		257912	Olfr948	-2.16021
		17773	Mtnr1a	-1.58287
		258350	Olfr706	-1.56576

		16867	Lhcgr	-1.37102
		258173	Olfr1532-ps1	-1.3396
		57811	Rgr	-1.28031
		18386	Oprd1	-1.27226
		15551	Htr1b	-1.2463
		14309	Fshr	-1.17472
		15566	Htr7	-1.1364
		15562	Htr4	-1.03209
		18166	Npy1r	-1.02075
		12772	Ccr2	-1.00727
		12773	Ccr4	-1.00322
		19204	Ptafr	-0.97951
		11555	Adrb2	-0.92768
		12776	Ccr8	-0.85933
		14062	F2r	-0.61231
		19219	*Ptger4*	*-0.59249*
		233571	P2ry6	-0.58524
		**18441**	**P2ry1**	**0.758201**
		**12672**	**Chrm4**	**0.850005**
		**13491**	**Drd4**	**0.875113**
		**258269**	**Olfr930**	**0.892154**
		**258499**	**Olfr945**	**0.933335**
		**14294**	**Fpr3**	**0.944948**
		**257959**	**Olfr1537**	**0.981626**
		**233670**	**Olfr6**	**1.010129**
		**14765**	**Gpr50**	**1.018872**
		**258882**	**Olfr874**	**1.141852**
		**15552**	**Htr1d**	**1.221482**
		**12000**	**Avpr2**	**1.251924**
		**57385**	**P2ry4**	**1.327103**
		**258046**	**Olfr951**	**1.336804**
		**18167**	**Npy2r**	**1.341246**
		**14761**	**Gpr27**	**1.471809**
		**171469**	**Gpr37l1**	**1.787234**
		**11541**	**Adora2b**	**1.805445**
		**11551**	**Adra2a**	**2.836444**
	GPCRs, Other	14608	Gpr83	-3.21469
		17202	Mc4r	-2.32772
		64378	Gpr88	-2.30643
		113847	Vmn1r43	-1.9454
		113846	Vmn1r47	-1.92418

		18332	Olfr33	-1.90223
		81015	Vmn1r56	-1.62035
		18344	Olfr45	-1.52067
		258343	Olfr397	-1.36572
		81016	Vmn1r62	-1.30782
		113848	Vmn1r42	-1.29014
		18346	Olfr47	-1.18329
		64379	Irx6	-1.01392
		81017	Vmn1r63	-0.9283
		11555	Adrb2	-0.92768
		14362	*Fzd1*	*-0.81648*
		244701	Mtnr1b	-0.7353
		26364	Adgre5	-0.60643
		**71111**	**Gpr39**	**0.728031**
		**18322**	**Olfr24**	**0.762628**
		**76206**	**Gpr165**	**0.849664**
		**18331**	**Olfr32**	**0.906447**
		**13492**	**Drd5**	**0.966222**
		**233670**	**Olfr6**	**1.010129**
		**15552**	**Htr1d**	**1.221482**
		**257890**	**Olfr12**	**1.339785**
		**113850**	**V1ra8**	**1.713639**
		**18347**	**Olfr48**	**1.776151**
		**171469**	**Gpr37l1**	**1.787234**
		**258859**	**Olfr161**	**3.109598**
	Non-odorant GPCRs	26361	Avpr1b	-2.92801
		14652	Glp1r	-2.36498
		17202	Mc4r	-2.32772
		387347	Tas2r118	-2.1135
		236781	Gpr119	-1.79257
		574417	Tas2r137	-1.7196
		667992	Tas2r103	-1.64983
		17773	Mtnr1a	-1.58287
		93746	Gprc5d	-1.37834
		16867	Lhcgr	-1.37102
		268934	Grm4	-1.33684
		57811	Rgr	-1.28031
		18386	Oprd1	-1.27226
		15551	Htr1b	-1.2463
		14309	Fshr	-1.17472
		15566	Htr7	-1.1364

		21336	Tacr1	-1.12491
		99296	Hrh3	-1.05305
		242093	Rxfp4	-1.04311
		15562	Htr4	-1.03209
		18166	Npy1r	-1.02075
		12772	Ccr2	-1.00727
		12773	Ccr4	-1.00322
		19204	Ptafr	-0.97951
		11555	Adrb2	-0.92768
		227289	Gpbar1	-0.88141
		12776	Ccr8	-0.85933
		14362	*Fzd1*	*-0.81648*
		244701	Mtnr1b	-0.7353
		53978	Lpar2	-0.61346
		14062	F2r	-0.61231
		26364	Adgre5	-0.60643
		19219	*Ptger4*	*-0.59249*
		233571	P2ry6	-0.58524
		**14370**	**Fzd8**	**0.631495**
		**12921**	**Crhr1**	**0.757531**
		**18441**	**P2ry1**	**0.758201**
		**71223**	**Gpr15**	**0.793225**
		**65086**	**Lpar3**	**0.832089**
		**12672**	**Chrm4**	**0.850005**
		**11517**	**Adcyap1r1**	**0.851143**
		**13491**	**Drd4**	**0.875113**
		**13492**	**Drd5**	**0.966222**
		**14765**	**Gpr50**	**1.018872**
		**15552**	**Htr1d**	**1.221482**
		**12000**	**Avpr2**	**1.251924**
		**57385**	**P2ry4**	**1.327103**
		**18167**	**Npy2r**	**1.341246**
		**107831**	**Adgrb1**	**1.711902**
		**11541**	**Adora2b**	**1.805445**
		**11551**	**Adra2a**	**2.836444**
	Class A/1 (Rhodopsin-like receptors)	23919	Insl5	-4.39738
		26361	Avpr1b	-2.92801
		17202	Mc4r	-2.32772
		19064	Ppy	-2.23412
		217212	Pyy	-2.12279
		17773	Mtnr1a	-1.58287

		16867	Lhcgr	-1.37102
		18386	Oprd1	-1.27226
		58991	Ghrl	-1.26899
		15551	Htr1b	-1.2463
		16952	Anxa1	-1.18898
		14309	Fshr	-1.17472
		15566	Htr7	-1.1364
		21336	Tacr1	-1.12491
		99296	Hrh3	-1.05305
		242093	Rxfp4	-1.04311
		15562	Htr4	-1.03209
		18166	Npy1r	-1.02075
		12773	Ccr4	-1.00322
		13614	*Edn1*	*-0.99296*
		19204	Ptafr	-0.97951
		21333	Tac1	-0.97278
		11555	Adrb2	-0.92768
		227289	Gpbar1	-0.88141
		11606	Agt	-0.80285
		18829	Ccl21a	-0.76839
		244701	Mtnr1b	-0.7353
		319197	Gpr4	-0.63098
		53978	Lpar2	-0.61346
		14062	F2r	-0.61231
		19219	*Ptger4*	*-0.59249*
		233571	P2ry6	-0.58524
		**18441**	**P2ry1**	**0.758201**
		**65086**	**Lpar3**	**0.832089**
		**12672**	**Chrm4**	**0.850005**
		**321020**	**Fpr-rs6**	**0.856069**
		**13491**	**Drd4**	**0.875113**
		**14294**	**Fpr3**	**0.944948**
		**13492**	**Drd5**	**0.966222**
		**15552**	**Htr1d**	**1.221482**
		**12000**	**Avpr2**	**1.251924**
		**20300**	**Ccl25**	**1.322852**
		**57385**	**P2ry4**	**1.327103**
		**18167**	**Npy2r**	**1.341246**
		**22044**	**Trh**	**1.380194**
		**252837**	**Ackr4**	**1.449683**
		**11541**	**Adora2b**	**1.805445**

		**67405**	**Nts**	**3.057371**
	G alpha (i) signalling events	23919	Insl5	-4.39738
		19064	Ppy	-2.23412
		217212	Pyy	-2.12279
		387347	Tas2r118	-2.1135
		574417	Tas2r137	-1.7196
		667992	Tas2r103	-1.64983
		17773	Mtnr1a	-1.58287
		268934	Grm4	-1.33684
		18386	Oprd1	-1.27226
		15551	Htr1b	-1.2463
		16952	Anxa1	-1.18898
		99296	Hrh3	-1.05305
		242093	Rxfp4	-1.04311
		18166	Npy1r	-1.02075
		12772	Ccr2	-1.00727
		12773	Ccr4	-1.00322
		24012	Rgs7	-0.96881
		12776	Ccr8	-0.85933
		11606	Agt	-0.80285
		56470	Rgs19	-0.78909
		11513	Adcy7	-0.76956
		18829	Ccl21a	-0.76839
		244701	Mtnr1b	-0.7353
		53978	Lpar2	-0.61346
		**387343**	**Tas2r109**	**0.735099**
		**65086**	**Lpar3**	**0.832089**
		**12672**	**Chrm4**	**0.850005**
		**321020**	**Fpr-rs6**	**0.856069**
		**13491**	**Drd4**	**0.875113**
		**14294**	**Fpr3**	**0.944948**
		**387356**	**Tas2r131**	**0.958132**
		**15552**	**Htr1d**	**1.221482**
		**20300**	**Ccl25**	**1.322852**
		**57385**	**P2ry4**	**1.327103**
		**18167**	**Npy2r**	**1.341246**
	Taste transduction	387347	Tas2r118	-2.1135
		83770	Tas1r2	-1.84156
		574417	Tas2r137	-1.7196
		667992	Tas2r103	-1.64983
		268934	Grm4	-1.33684

		18796	Plcb2	-0.92128
		18749	Prkacb	-0.69222
		18573	Pde1a	-0.58797
		**83771**	**Tas1r3**	**0.629121**
		**387343**	**Tas2r109**	**0.735099**
		**387356**	**Tas2r131**	**0.958132**
		**20277**	**Scnn1b**	**1.023555**
	Monoamine GPCRs	15551	Htr1b	-1.2463
		15566	Htr7	-1.1364
		15562	Htr4	-1.03209
		11555	Adrb2	-0.92768
		**12672**	**Chrm4**	**0.850005**
		**13491**	**Drd4**	**0.875113**
		**13492**	**Drd5**	**0.966222**
		**15552**	**Htr1d**	**1.221482**
		**11551**	**Adra2a**	**2.836444**
	G alpha (s) signalling events	14652	Glp1r	-2.36498
		17202	Mc4r	-2.32772
		16867	Lhcgr	-1.37102
		14309	Fshr	-1.17472
		15566	Htr7	-1.1364
		15562	Htr4	-1.03209
		11555	Adrb2	-0.92768
		227289	Gpbar1	-0.88141
		19227	Pthlh	-0.88025
		18583	Pde7a	-0.84692
		11513	Adcy7	-0.76956
		19219	*Ptger4*	*-0.59249*
		18573	Pde1a	-0.58797
		**12921**	**Crhr1**	**0.757531**
		**11517**	**Adcyap1r1**	**0.851143**
		**13492**	**Drd5**	**0.966222**
		**12000**	**Avpr2**	**1.251924**
		**11541**	**Adora2b**	**1.805445**
		**116903**	**Calcb**	**2.19162**
	G alpha (q) signalling events	26361	Avpr1b	-2.92801
		14459	Gast	-1.80179
		58991	Ghrl	-1.26899
		16952	Anxa1	-1.18898
		22068	Trpc6	-1.15821
		21336	Tacr1	-1.12491

		14675	Gna14	-1.07872
		13614	*Edn1*	*-0.99296*
		19204	Ptafr	-0.97951
		21333	Tac1	-0.97278
		18796	Plcb2	-0.92128
		11606	Agt	-0.80285
		217480	Dgkb	-0.80037
		56470	Rgs19	-0.78909
		319197	Gpr4	-0.63098
		53978	Lpar2	-0.61346
		14062	F2r	-0.61231
		233571	P2ry6	-0.58524
		**18441**	**P2ry1**	**0.758201**
		**320127**	**Dgki**	**0.800134**
		**65086**	**Lpar3**	**0.832089**
		**321020**	**Fpr-rs6**	**0.856069**
		**14294**	**Fpr3**	**0.944948**
		**14676**	**Gna15**	**0.956512**
		**22044**	**Trh**	**1.380194**
		**26946**	**Trpc7**	**1.470884**
		**67405**	**Nts**	**3.057371**
	Class C/3 (Metabotropic glutamate/pheromone receptors)	387347	Tas2r118	-2.1135
		83770	Tas1r2	-1.84156
		574417	Tas2r137	-1.7196
		667992	Tas2r103	-1.64983
		268934	Grm4	-1.33684
		**83771**	**Tas1r3**	**0.629121**
		**387343**	**Tas2r109**	**0.735099**
		**387356**	**Tas2r131**	**0.958132**
	Amine ligand-binding receptors	15551	Htr1b	-1.2463
		15566	Htr7	-1.1364
		99296	Hrh3	-1.05305
		15562	Htr4	-1.03209
		11555	Adrb2	-0.92768
		**12672**	**Chrm4**	**0.850005**
		**13491**	**Drd4**	**0.875113**
		**13492**	**Drd5**	**0.966222**
		**15552**	**Htr1d**	**1.221482**
Xenobiotics metabolism	Phase 1 - Functionalization of compounds	11522	Adh1	-1.67577
		21391	Tbxas1	-0.73684
		14261	Fmo1	-0.72381

		**74519**	**Cyp2j9**	**0.814402**
		**13115**	**Cyp27b1**	**0.848137**
		**13110**	**Cyp2j6**	**0.907245**
		**13119**	**Cyp4a14**	**0.98224**
		**665095**	**Cyp2j8**	**0.987507**
		**26358**	**Aldh1a7**	**1.035068**
		**13097**	**Cyp2c38**	**1.138673**
		**13112**	**Cyp3a11**	**1.348164**
		**244209**	**Cyp2r1**	**1.52365**
		**13117**	**Cyp4a10**	**1.548098**
		**13089**	**Cyp2b13**	**1.987011**
		**11668**	**Aldh1a1**	**2.031233**
		**72303**	**Cyp2c65**	**2.567495**
		**13120**	**Cyp4b1**	**2.85414**
		**69888**	**Cyp2c66**	**3.077231**
		**13114**	**Cyp3a16**	**3.547892**
	Biological oxidations	17962	Nat3	-2.67001
		11522	Adh1	-1.67577
		21391	Tbxas1	-0.73684
		14261	Fmo1	-0.72381
		**14630**	**Gclm**	**0.716835**
		**22017**	**Tpmt**	**0.71723**
		**242585**	**Slc35d1**	**0.769778**
		**68214**	**Gsto2**	**0.779804**
		**74519**	**Cyp2j9**	**0.814402**
		**14629**	**Gclc**	**0.845164**
		**13115**	**Cyp27b1**	**0.848137**
		**13110**	**Cyp2j6**	**0.907245**
		**13119**	**Cyp4a14**	**0.98224**
		**665095**	**Cyp2j8**	**0.987507**
		**14873**	**Gsto1**	**1.021687**
		**26358**	**Aldh1a7**	**1.035068**
		**94284**	**Ugt1a6a**	**1.076336**
		**13097**	**Cyp2c38**	**1.138673**
		**22235**	**Ugdh**	**1.1947**
		**13112**	**Cyp3a11**	**1.348164**
		**244209**	**Cyp2r1**	**1.52365**
		**231583**	**Slc26a1**	**1.545867**
		**13117**	**Cyp4a10**	**1.548098**
		**13089**	**Cyp2b13**	**1.987011**
		**11668**	**Aldh1a1**	**2.031233**

		**72303**	**Cyp2c65**	**2.567495**
		**13120**	**Cyp4b1**	**2.85414**
		**69888**	**Cyp2c66**	**3.077231**
		**13114**	**Cyp3a16**	**3.547892**
	Drug metabolism - other enzymes	17962	Nat3	-2.67001
		72361	Ces2g	-0.84415
		**22017**	**Tpmt**	**0.71723**
		**94284**	**Ugt1a6a**	**1.076336**
		**394430**	**Ugt1a10**	**1.138556**
		**13112**	**Cyp3a11**	**1.348164**
		**394435**	**Ugt1a6b**	**1.551736**
		**99586**	**Dpyd**	**1.940333**
		**13897**	**Ces1e**	**2.017295**
		**104158**	**Ces1d**	**2.38827**
		**112417**	**Ugt2b37**	**2.589247**
		**13114**	**Cyp3a16**	**3.547892**
		**100559**	**Ugt2b38**	**4.110325**
	Drug metabolism - cytochrome P450	11522	Adh1	-1.67577
		226143	Cyp2c44	-1.0174
		14261	Fmo1	-0.72381
		68214	Gsto2	0.779804
		**11532**	**Adh5**	**0.855127**
		**14873**	**Gsto1**	**1.021687**
		**94284**	**Ugt1a6a**	**1.076336**
		**394430**	**Ugt1a10**	**1.138556**
		**13097**	**Cyp2c38**	**1.138673**
		**13112**	**Cyp3a11**	**1.348164**
		**394435**	**Ugt1a6b**	**1.551736**
		**13089**	**Cyp2b13**	**1.987011**
		**72303**	**Cyp2c65**	**2.567495**
		**112417**	**Ugt2b37**	**2.589247**
		**69888**	**Cyp2c66**	**3.077231**
		**13114**	**Cyp3a16**	**3.547892**
		**100559**	**Ugt2b38**	**4.110325**
	Cytochrome P450 - arranged by substrate type	21391	Tbxas1	-0.73684
		**74519**	**Cyp2j9**	**0.814402**
		**13115**	**Cyp27b1**	**0.848137**
		**13110**	**Cyp2j6**	**0.907245**
		**13119**	**Cyp4a14**	**0.98224**
		**665095**	**Cyp2j8**	**0.987507**
		**13097**	**Cyp2c38**	**1.138673**

		**13112**	**Cyp3a11**	**1.348164**
		**244209**	**Cyp2r1**	**1.52365**
		**13117**	**Cyp4a10**	**1.548098**
		**13089**	**Cyp2b13**	**1.987011**
		**72303**	**Cyp2c65**	**2.567495**
		**13120**	**Cyp4b1**	**2.85414**
		**69888**	**Cyp2c66**	**3.077231**
		**13114**	**Cyp3a16**	**3.547892**
Metabolism	Steroid hormone biosynthesis	15495	Hsd3b4	-2.1935
		15485	Hsd17b1	-1.70289
		94224	Srd5a2	-1.65185
		**13079**	**Cyp21a1**	**0.882118**
		**94284**	**Ugt1a6a**	**1.076336**
		**394430**	**Ugt1a10**	**1.138556**
		**13112**	**Cyp3a11**	**1.348164**
		**394435**	**Ugt1a6b**	**1.551736**
		**27400**	**Hsd17b6**	**1.632182**
		**105349**	**Akr1c18**	**1.966455**
		**112417**	**Ugt2b37**	**2.589247**
		**13114**	**Cyp3a16**	**3.547892**
		**100559**	**Ugt2b38**	**4.110325**
	Steroid hormones	15495	Hsd3b4	-2.1935
		15485	Hsd17b1	-1.70289
		14725	Lrp2	-1.65004
		11677	Akr1b3	-0.65276
		**13115**	**Cyp27b1**	**0.848137**
		**170460**	**Stard5**	**1.02616**
		**244209**	**Cyp2r1**	**1.52365**
		**65969**	**Cubn**	**1.724354**
	Retinol metabolism	11522	Adh1	-1.67577
		226143	Cyp2c44	-1.0174
		**67442**	**Retsat**	**0.81903**
		**11532**	**Adh5**	**0.855127**
		**13119**	**Cyp4a14**	**0.98224**
		**26358**	**Aldh1a7**	**1.035068**
		**94284**	**Ugt1a6a**	**1.076336**
		**394430**	**Ugt1a10**	**1.138556**
		**13097**	**Cyp2c38**	**1.138673**
		**13112**	**Cyp3a11**	**1.348164**
		**13117**	**Cyp4a10**	**1.548098**
		**394435**	**Ugt1a6b**	**1.551736**

		**27400**	**Hsd17b6**	**1.632182**
		**13089**	**Cyp2b13**	**1.987011**
		**11668**	**Aldh1a1**	**2.031233**
		**72303**	**Cyp2c65**	**2.567495**
		**112417**	**Ugt2b37**	**2.589247**
		**69888**	**Cyp2c66**	**3.077231**
		**13114**	**Cyp3a16**	**3.547892**
		**100559**	**Ugt2b38**	**4.110325**
	Ascorbate and aldarate metabolism	268756	Gulo	-1.23563
		56727	Miox	-0.60614
		**11671**	**Aldh3a2**	**0.748732**
		**94284**	**Ugt1a6a**	**1.076336**
		**394430**	**Ugt1a10**	**1.138556**
		**22235**	**Ugdh**	**1.1947**
		**394435**	**Ugt1a6b**	**1.551736**
		**112417**	**Ugt2b37**	**2.589247**
		**100559**	**Ugt2b38**	**4.110325**
	Vitamin D (calciferol) metabolism	14725	Lrp2	-1.65004
		**13115**	**Cyp27b1**	**0.848137**
		**244209**	**Cyp2r1**	**1.52365**
		**65969**	**Cubn**	**1.724354**
Cancer signalling	Chemical carcinogenesis	17962	Nat3	-2.67001
		11441	Chrna7	-2.35055
		11522	Adh1	-1.67577
		226143	Cyp2c44	-1.0174
		**68214**	**Gsto2**	**0.779804**
		**11532**	**Adh5**	**0.855127**
		**14873**	**Gsto1**	**1.021687**
		**94284**	**Ugt1a6a**	**1.076336**
		**394430**	**Ugt1a10**	**1.138556**
		**13097**	**Cyp2c38**	**1.138673**
		**13112**	**Cyp3a11**	**1.348164**
		**394435**	**Ugt1a6b**	**1.551736**
		**13089**	**Cyp2b13**	**1.987011**
		**72303**	**Cyp2c65**	**2.567495**
		**112417**	**Ugt2b37**	**2.589247**
		**69888**	**Cyp2c66**	**3.077231**
		**13114**	**Cyp3a16**	**3.547892**
		**100559**	**Ugt2b38**	**4.110325**
Digestive system	Recycling of bile acids and salts	11657	Alb	-2.74905
		28248	Slco1a1	-0.71482

		**28250**	**Slco1a4**	**0.702863**
		**76408**	**Abcc3**	**0.938123**
		**12012**	**Baat**	**1.013347**
		**20494**	**Slc10a2**	**1.82269**
		**16204**	**Fabp6**	**1.881719**
	Fatty acid degradation	11522	Adh1	-1.67577
		**52538**	**Acaa2**	**0.61326**
		**13177**	**Eci1**	**0.621129**
		**66885**	**Acadsb**	**0.666439**
		**113868**	**Acaa1a**	**0.689297**
		**231086**	**Hadhb**	**0.706245**
		**11671**	**Aldh3a2**	**0.748732**
		**328845**	**Acsbg2**	**0.760246**
		**11532**	**Adh5**	**0.855127**
		**235674**	**Acaa1b**	**0.869636**
		**11409**	**Acads**	**0.887581**
		**13119**	**Cyp4a14**	**0.98224**
		**13117**	**Cyp4a10**	**1.548098**
	Linoleic acid metabolism	271844	Pla2g4f	-2.42912
		26971	Pla2g2f	-2.37882
		226143	Cyp2c44	-1.0174
		**74519**	**Cyp2j9**	**0.814402**
		**13110**	**Cyp2j6**	**0.907245**
		**665095**	**Cyp2j8**	**0.987507**
		**13097**	**Cyp2c38**	**1.138673**
		**69836**	**Pla2g12b**	**1.148404**
		**13112**	**Cyp3a11**	**1.348164**
		**72303**	**Cyp2c65**	**2.567495**
		**69888**	**Cyp2c66**	**3.077231**
		**13114**	**Cyp3a16**	**3.547892**
	Bile secretion	11513	Adcy7	-0.76956
		28248	Slco1a1	-0.71482
		18749	Prkacb	-0.69222
		**232975**	**Atp1a3**	**0.693361**
		**28250**	**Slco1a4**	**0.702863**
		**19879**	**Slc22a8**	**0.786054**
		**20181**	**Rxra**	**0.864973**
		**20535**	**Slc4a2**	**0.871771**
		**11826**	**Aqp1**	**0.907644**
		**320024**	**Nceh1**	**0.933867**
		**18669**	**Abcb1b**	**0.937074**

		**76408**	**Abcc3**	**0.938123**
		**20186**	**Nr1h4**	**0.960415**
		**12012**	**Baat**	**1.013347**
		**20494**	**Slc10a2**	**1.82269**
		**106407**	**Slc51a**	**4.426367**
	Fatty acids	**74519**	**Cyp2j9**	**0.814402**
		**13110**	**Cyp2j6**	**0.907245**
		**13119**	**Cyp4a14**	**0.98224**
		**665095**	**Cyp2j8**	**0.987507**
		**13117**	**Cyp4a10**	**1.548098**
		**13120**	**Cyp4b1**	**2.85414**
Transport of molecules	Transmembrane transport of small molecules	108017	Fxyd4	-3.68939
		192113	Atp12a	-3.62413
		11657	Alb	-2.74905
		56643	Slc15a1	-2.27435
		20538	Slc6a2	-2.24272
		242341	Atp6v0d2	-2.21608
		114304	Slc28a3	-2.10056
		338375	Atp6v1g3	-2.04699
		64177	Trpv6	-1.87407
		243616	Slc6a11	-1.32681
		101772	Ano1	-1.271
		236727	Slc9a7	-1.24181
		11827	Aqp2	-1.22025
		52466	Slc46a1	-1.1807
		140919	Slc17a6	-1.17003
		22068	Trpc6	-1.15821
		14377	G6pc	-1.15001
		69047	Atp2c2	-1.14854
		20531	Slc34a2	-1.10194
		193034	Trpv1	-1.09319
		22368	Trpv2	-1.04169
		53881	Slc5a3	-1.03602
		215335	Slc36a1	-0.77241
		381072	Abca17	-0.77097
		11513	Adcy7	-0.76956
		20513	Slc1a6	-0.73881
		14710	Gngt2	-0.72984
		28248	Slco1a1	-0.71482
		108116	Slco3a1	-0.70872
		17364	Trpm1	-0.7033

		18749	Prkacb	-0.69222
		57274	Slc16a8	-0.68227
		11977	Atp7a	-0.67643
		338370	Nalcn	-0.62935
		22041	Trf	-0.6144
		**56517**	**Slc22a21**	**0.601124**
		**230612**	**Slc5a9**	**0.66179**
		**232975**	**Atp1a3**	**0.693361**
		**28250**	**Slco1a4**	**0.702863**
		**22782**	**Slc30a1**	**0.719359**
		**101488**	**Slco2b1**	**0.727811**
		**242585**	**Slc35d1**	**0.769778**
		**26457**	**Slc27a1**	**0.77524**
		**27376**	**Slc25a10**	**0.778687**
		**27416**	**Abcc5**	**0.785076**
		**19879**	**Slc22a8**	**0.786054**
		**14401**	**Gabrb2**	**0.829711**
		**26569**	**Slc27a4**	**0.832769**
		**70484**	**Slc35d2**	**0.868659**
		**20535**	**Slc4a2**	**0.871771**
		**380701**	**Slc47a2**	**0.898028**
		**11826**	**Aqp1**	**0.907644**
		**18669**	**Abcb1b**	**0.937074**
		**76408**	**Abcc3**	**0.938123**
		**68667**	**Trpm4**	**0.951637**
		**74915**	**Atp6v1e2**	**0.979016**
		**216227**	**Slc17a8**	**0.992313**
		**11945**	**Atp4b**	**0.994119**
		**58176**	**Rhbg**	**1.005961**
		**20277**	**Scnn1b**	**1.023555**
		**24115**	**Best1**	**1.027966**
		**21366**	**Slc6a6**	**1.050288**
		**110886**	**Gabra5**	**1.055243**
		**20541**	**Slc8a1**	**1.065224**
		**20533**	**Slc4a1**	**1.10896**
		**74104**	**Abcb6**	**1.120286**
		**20539**	**Slc7a5**	**1.133807**
		**237831**	**Slc13a5**	**1.161357**
		**67547**	**Slc39a8**	**1.210859**
		**12000**	**Avpr2**	**1.251924**
		**26946**	**Trpc7**	**1.470884**

		**30805**	**Slc22a4**	**1.524987**
		**231583**	**Slc26a1**	**1.545867**
		**20519**	**Slc22a3**	**1.948204**
		**67473**	**Slc47a1**	**2.161129**
		**74338**	**Slc6a19**	**2.361178**
	SLC-mediated transmembrane transport	11657	Alb	-2.74905
		56643	Slc15a1	-2.27435
		20538	Slc6a2	-2.24272
		114304	Slc28a3	-2.10056
		243616	Slc6a11	-1.32681
		236727	Slc9a7	-1.24181
		140919	Slc17a6	-1.17003
		14377	G6pc	-1.15001
		20531	Slc34a2	-1.10194
		53881	Slc5a3	-1.03602
		215335	Slc36a1	-0.77241
		20513	Slc1a6	-0.73881
		28248	Slco1a1	-0.71482
		108116	Slco3a1	-0.70872
		57274	Slc16a8	-0.68227
		**56517**	**Slc22a21**	**0.601124**
		**230612**	**Slc5a9**	**0.66179**
		**28250**	**Slco1a4**	**0.702863**
		**22782**	**Slc30a1**	**0.719359**
		**101488**	**Slco2b1**	**0.727811**
		**242585**	**Slc35d1**	**0.769778**
		**26457**	**Slc27a1**	**0.77524**
		**27376**	**Slc25a10**	**0.778687**
		**19879**	**Slc22a8**	**0.786054**
		**26569**	**Slc27a4**	**0.832769**
		**70484**	**Slc35d2**	**0.868659**
		**20535**	**Slc4a2**	**0.871771**
		**380701**	**Slc47a2**	**0.898028**
		**216227**	**Slc17a8**	**0.992313**
		**58176**	**Rhbg**	**1.005961**
		**21366**	**Slc6a6**	**1.050288**
		**20541**	**Slc8a1**	**1.065224**
		**20533**	**Slc4a1**	**1.10896**
		**20539**	**Slc7a5**	**1.133807**
		**237831**	**Slc13a5**	**1.161357**
		**67547**	**Slc39a8**	**1.210859**

		**30805**	**Slc22a4**	**1.524987**
		**231583**	**Slc26a1**	**1.545867**
		**20519**	**Slc22a3**	**1.948204**
		**67473**	**Slc47a1**	**2.161129**
		**74338**	**Slc6a19**	**2.361178**
	Transport of vitamins, nucleosides, and related molecules	11657	Alb	-2.74905
		114304	Slc28a3	-2.10056
		28248	Slco1a1	-0.71482
		108116	Slco3a1	-0.70872
		**28250**	**Slco1a4**	**0.702863**
		**101488**	**Slco2b1**	**0.727811**
		**242585**	**Slc35d1**	**0.769778**
		**26457**	**Slc27a1**	**0.77524**
		**26569**	**Slc27a4**	**0.832769**
		**70484**	**Slc35d2**	**0.868659**
Neuronal response	Neuroactive ligand-receptor interaction	14608	Gpr83	-3.21469
		26361	Avpr1b	-2.92801
		14652	Glp1r	-2.36498
		11441	Chrna7	-2.35055
		17202	Mc4r	-2.32772
		209513	Taar4	-1.97343
		17773	Mtnr1a	-1.58287
		16867	Lhcgr	-1.37102
		209517	Taar7b	-1.36067
		268934	Grm4	-1.33684
		18386	Oprd1	-1.27226
		15551	Htr1b	-1.2463
		14309	Fshr	-1.17472
		15566	Htr7	-1.1364
		21336	Tacr1	-1.12491
		193034	Trpv1	-1.09319
		99296	Hrh3	-1.05305
		15562	Htr4	-1.03209
		18166	Npy1r	-1.02075
		18815	Plg	-1.00547
		435207	Taar7f	-0.98449
		19204	Ptafr	-0.97951
		11555	Adrb2	-0.92768
		244701	Mtnr1b	-0.7353
		53978	Lpar2	-0.61346
		14062	F2r	-0.61231

		19219	*Ptger4*	*-0.59249*
		233571	P2ry6	-0.58524
		**14403**	**Gabrd**	**0.701059**
		**12921**	**Crhr1**	**0.757531**
		**18441**	**P2ry1**	**0.758201**
		**14401**	**Gabrb2**	**0.829711**
		**65086**	**Lpar3**	**0.832089**
		**12672**	**Chrm4**	**0.850005**
		**11517**	**Adcyap1r1**	**0.851143**
		**14812**	**Grin2b**	**0.871054**
		**13491**	**Drd4**	**0.875113**
		**14294**	**Fpr3**	**0.944948**
		**13492**	**Drd5**	**0.966222**
		**14765**	**Gpr50**	**1.018872**
		**14599**	**Gh**	**1.043182**
		**110886**	**Gabra5**	**1.055243**
		**15552**	**Htr1d**	**1.221482**
		**12000**	**Avpr2**	**1.251924**
		**215854**	**Taar5**	**1.272795**
		**57385**	**P2ry4**	**1.327103**
		**18167**	**Npy2r**	**1.341246**
		**19116**	**Prlr**	**1.437521**
		**14938**	**Gzma**	**1.654691**
		**11541**	**Adora2b**	**1.805445**
		**108043**	**Chrnb3**	**1.954763**
		**11551**	**Adra2a**	**2.836444**
	Serotonin receptors	15551	Htr1b	-1.2463
		15566	Htr7	-1.1364
		15562	Htr4	-1.03209
		**15552**	**Htr1d**	**1.221482**

Supplementary Table 4: Details results of ORA after 21 days of exposure of E171 in BALB/c mice in combination with AOM/DSS. Numbers in bold correspond to upregulated genes. Log2FC= Log2 fold change obtained with LIMMA script with correction for its own time-matched control.

Group of pathways	Pathways	EntrezGeneID	Genes	Log2FC
Signalling	Olfactory transduction	258439	Olfr1309	-3.08981
		258879	Olfr330	-2.76283
		258692	Olfr1442	-2.5026
		404335	Olfr1535	-2.39943
		258766	Olfr259	-1.93446
		258969	Olfr1226	-1.68754
		258751	Olfr608	-1.55582
		258689	Olfr1466	-1.47231
		18315	Olfr18	-1.39071
		258820	Olfr1184	-1.38599
		18345	Olfr46	-1.27984
		258549	Olfr798	-1.17812
		258633	Olfr1153	-1.03563
		258207	Olfr452	-1.00468
		258772	Olfr821	-0.99624
		258392	Olfr190	-0.95696
		258499	Olfr945	-0.91291
		259016	Olfr1048	-0.90774
		258464	Olfr1384	-0.87283
		12325	Camk2g	-0.85629
		258887	Olfr1294	-0.84054
		258477	Olfr197	-0.77396
		258532	Olfr373	-0.72847
		258713	Olfr430	-0.69518
		258502	Olfr279	-0.61717
		258484	Olfr1410	-0.60585
		258527	Olfr1368	-0.60206
		320129	Adrbk2	-0.59922
		258815	Olfr1218	-0.59461
		**14913**	**Guca1a**	**0.626273**
		**258825**	**Olfr975**	**0.817423**
		**258976**	**Olfr1262**	**1.383201**
		**258381**	**Olfr460**	**1.464693**
		**258961**	**Olfr631**	**1.968851**
	G Protein signalling Pathways	18761	Prkcq	-1.34532

		18751	Prkcb	-1.18634
		29863	Pde7b	-1.14388
		319757	*Smo*	*-0.90040*
		19057	Ppp3cc	-1.00873
		14695	Gnb3	-0.97877
		238276	Akap5	-0.94338
		11513	Adcy7	-0.86202
		14673	Gna12	-0.86126
		16801	Arhgef1	-0.70168
		14687	Gnaz	-0.69642
		83397	Akap12	-0.64937
		14706	Gng4	-0.63464
Immune response	Cell adhesion molecules (CAMs)	192167	Nlgn1	-2.08356
		16401	Itga4	-1.32152
		224754	H2-M11	-1.29746
		15894	Icam1	-1.21248
		60533	Cd274	-1.03935
		20737	Spn	-1.03922
		16403	Itga6	-1.03136
		20970	Sdc3	-0.97302
		80883	Ntng1	-0.96274
		12562	Cdh5	-0.93428
		15042	H2-T24	-0.93002
		16414	Itgb2	-0.91394
		320910	Itgb8	-0.89441
		12524	Cd86	-0.84888
		12504	Cd4	-0.81738
		245537	Nlgn3	-0.80663
		18417	Cldn11	-0.78674
		12805	Cntn1	-0.73044
		15019	H2-Q8	-0.72762
		50723	Icosl	-0.7219
		14991	H2-M3	-0.69415
		547349	LOC547349	-0.66324
	Innate Immune System	13429	Dnm1	-0.62205
		12504	Cd4	-0.81738
		16414	Itgb2	-0.91394
		170743	Tlr7	-0.72769
		56743	Lat2	-0.65261
		76884	Cyfip2	-1.52672
		140579	Elmo2	-0.81886

		14360	Fyn	-0.63732
		103967	Dnm3	-0.80914
		12628	Cfh	-1.02214
		12268	C4b	-1.13577
		195046	Nlrp1a	-0.68831
		18595	Pdgfra	-1.07342
		317677	C1s2	-0.81699
		16885	Limk1	-0.60718
		108960	Irak2	-0.82573
		328572	Ep300	-0.9958
		11820	App	-0.60252
		16150	Ikbkb	-0.63611
		12326	Camk4	-1.42283
		50909	C1ra	-1.14292
		12503	Cd247	-1.25342
		80987	Nckipsd	-0.6101
		16635	Klra4	-2.32696
		15951	Ifi204	-1.02764
		18761	Prkcq	-1.34532
		100040462	Mndal	-1.56423
		217069	Trim25	-0.75227
		18591	Pdgfb	-0.63586
		12266	C3	-1.19742
		26414	Mapk10	-0.77253
		13869	Erbb4	-0.89524
		18186	Nrp1	-0.63787
		12524	Cd86	-0.84888
		11513	Adcy7	-0.86202
		57257	Vav3	-0.89394
		21898	Tlr4	-0.80896
		214403	Gm4788	-2.30009
		19200	Pstpip1	-0.77605
		320148	B430306N03Rik	-1.41436
		17079	Cd180	-1.41635
	Immune System	16635	Klra4	-2.32696
		214403	Gm4788	-2.30009
		16178	Il1r2	-2.02507
		100040462	Mndal	-1.56423
		76884	Cyfip2	-1.52672
		21354	Tap1	-1.5234
		56508	Rapgef4	-1.42788

		12326	Camk4	-1.42283
		17079	Cd180	-1.41635
		320148	B430306N03Rik	-1.41436
		16197	Il7r	-1.40769
		18761	Prkcq	-1.34532
		16401	Itga4	-1.32152
		12503	Cd247	-1.25342
		240168	Rasgrp3	-1.24337
		15894	Icam1	-1.21248
		12266	C3	-1.19742
		18751	Prkcb	-1.18634
		83490	Pik3ap1	-1.18421
		50909	C1ra	-1.14292
		12268	C4b	-1.13577
		16331	Inpp5d	-1.1318
		18595	Pdgfra	-1.07342
		20469	Sipa1	-1.05513
		60533	Cd274	-1.03935
		15951	Ifi204	-1.02764
		12628	Cfh	-1.02214
		328572	Ep300	-0.9958
		65256	Asb2	-0.93911
		16414	Itgb2	-0.91394
		13869	Erbb4	-0.89524
		57257	Vav3	-0.89394
		11513	Adcy7	-0.86202
		12325	Camk2g	-0.85629
		12524	Cd86	-0.84888
		108960	Irak2	-0.82573
		58194	Sh3kbp1	-0.82002
		140579	Elmo2	-0.81886
		12504	Cd4	-0.81738
		317677	C1s2	-0.81699
		103967	Dnm3	-0.80914
		21898	Tlr4	-0.80896
		14026	Evl	-0.79651
		19200	Pstpip1	-0.77605
		26414	Mapk10	-0.77253
		17470	Cd200	-0.76283
		217069	Trim25	-0.75227
		170743	Tlr7	-0.72769

		15019	H2-Q8	-0.72762
		74153	Uba7	-0.72612
		50723	Icosl	-0.7219
		14991	H2-M3	-0.69415
		195046	Nlrp1a	-0.68831
		11767	Ap1m1	-0.67679
		56743	Lat2	-0.65261
		18186	Nrp1	-0.63787
		14360	Fyn	-0.63732
		16150	Ikbkb	-0.63611
		18591	Pdgfb	-0.63586
		13429	Dnm1	-0.62205
		80987	Nckipsd	-0.6101
		16885	Limk1	-0.60718
		11820	App	-0.60252
Extracellular matrix	Extracellular matrix organization	240913	Adamts4	-2.6206
		16402	Itga5	-1.37853
		18453	P4hb	-1.36286
		16401	Itga4	-1.32152
		18198	Musk	-1.27138
		50701	Elane	-1.22146
		13004	Ncan	-1.22049
		15894	Icam1	-1.21248
		20970	Sdc3	-0.97302
		16399	Itga2b	-0.97158
		12153	Bmp1	-0.93515
		16414	Itgb2	-0.91394
		320910	Itgb8	-0.89441
		213945	Col28a1	-0.86825
		17390	*Mmp2*	*-0.82058*
		216725	Adamts2	-0.76482
		16779	Lamb2	-0.70709
		108075	Ltbp4	-0.70174
		14264	Fmod	-0.65589
		18591	Pdgfb	-0.63586
		237759	Col23a1	-0.62811
		21892	Tll1	-0.62532
		109711	Actn1	-0.61951
	Regulation of actin cytoskeleton	16399	Itga2b	-0.97158
		16401	Itga4	-1.32152
		16402	Itga5	-1.37853

		16403	Itga6	-1.03136
		16414	Itgb2	-0.91394
		76884	Cyfip2	-1.52672
		109711	Actn1	-0.61951
		18591	Pdgfb	-0.63586
		18595	Pdgfra	-1.07342
		20430	Cyfip1	-0.76709
		73341	Arhgef6	-0.72228
		12061	Bdkrb1	-0.90404
		17698	Msn	-1.01169
		14673	Gna12	-0.86126
		12672	Chrm4	-0.97074
		320910	Itgb8	-0.89441
		16801	Arhgef1	-0.70168
		57257	Vav3	-0.89394
		70584	Pak4	-0.76162
		16885	Limk1	-0.60718
		227753	Gsn	-0.6912
Neuronal response	Neuronal System	20511	Slc1a2	-1.95335
		12326	Camk4	-1.42283
		110902	Chrna2	-1.35204
		18751	Prkcb	-1.18634
		15166	Hcn2	-1.18138
		11448	Chrne	-1.04818
		14695	Gnb3	-0.97877
		16497	Kcnab1	-0.95345
		238276	Akap5	-0.94338
		16524	Kcnj9	-0.90225
		11513	Adcy7	-0.86202
		12325	Camk2g	-0.85629
		330953	Hcn4	-0.84286
		15168	Hcn3	-0.83643
		110637	Grik4	-0.77339
		11423	Ache	-0.77258
		14415	Gad1	-0.77245
		16528	Kcnk4	-0.75602
		20907	Stx1a	-0.74224
		54393	Gabbr1	-0.67084
		55984	Camkk1	-0.67046
		16511	Kcnh2	-0.66804
		14706	Gng4	-0.63464

		14814	Grin2d	-0.60631
		13162	Slc6a3	0.95093
	Transmission across Chemical Synapses	20511	Slc1a2	-1.95335
		12326	Camk4	-1.42283
		110902	Chrna2	-1.35204
		18751	Prkcb	-1.18634
		11448	Chrne	-1.04818
		14695	Gnb3	-0.97877
		238276	Akap5	-0.94338
		16524	Kcnj9	-0.90225
		11513	Adcy7	-0.86202
		12325	Camk2g	-0.85629
		110637	Grik4	-0.77339
		11423	Ache	-0.77258
		14415	Gad1	-0.77245
		20907	Stx1a	-0.74224
		54393	Gabbr1	-0.67084
		55984	Camkk1	-0.67046
		14706	Gng4	-0.63464
		14814	Grin2d	-0.60631
		**13162**	**Slc6a3**	**0.95093**
	Serotonin and anxiety-related events	56193	Plek	-1.08686
		192167	Nlgn1	-2.08356
		14814	Grin2d	-0.60631
		18751	Prkcb	-1.18634
	Neurotransmitter Receptor Binding And Downstream Transmission In The Postsynaptic Cell	12326	Camk4	-1.42283
		110902	Chrna2	-1.35204
		18751	Prkcb	-1.18634
		11448	Chrne	-1.04818
		14695	Gnb3	-0.97877
		238276	Akap5	-0.94338
		16524	Kcnj9	-0.90225
		11513	Adcy7	-0.86202
		12325	Camk2g	-0.85629
		110637	Grik4	-0.77339
		54393	Gabbr1	-0.67084
		55984	Camkk1	-0.67046
		14706	Gng4	-0.63464
		14814	Grin2d	-0.60631
	HCN channels	15166	Hcn2	-1.18138
		15168	Hcn3	-0.83643

		330953	Hcn4	-0.84286

Supplementary table 5: Result of the GSEA of the DEG after exposure to E171 in combination with AOM/DSS in the distal colon of BALB/c mice.

Days of exposure	Group of pathways	Pathways	q-value	Measured genes
2 days	Signalling	Olfactory transduction	0.183	31
7 days	Signalling	Phosphatidylinositol signalling system	4.58E-05	17
	Xenobiotics metabolism	Biological oxidations	0.000441	47
		Drug metabolism - other enzymes	0.00291	17
		Drug metabolism - cytochrome P450	0.00415	25
		Phase 1 - Functionalization of compounds	0.00514	30
		Metabolism of xenobiotics by cytochrome P450	0.0103	26
		Cytochrome P450 - arranged by substrate type	0.0138	23
		Synthesis of epoxy (EET) and dihydroxyeicosatrienoic acids (DHET)	0.0454	10
		Synthesis of (16-20)-hydroxyeicosatetraenoic acids (HETE)	0.0454	10
	Metabolism of protein	Metabolism	0.00291	253
		Inositol phosphate metabolism	0.0162	12
		Inositol phosphate metabolism	0.0162	13
		Linoleic acid metabolism	0.0185	16
		Pentose and glucuronate interconversions	0.0454	10
		Retinol metabolism	0.0454	19
	Immune response	Cytokine-cytokine receptor interaction	0.00113	67
		Hematopoietic cell lineage	0.0039	19
		Spinal Cord Injury	0.00415	23
		Staphylococcus aureus infection	0.00182	17
	Cancer signalling	Chemical carcinogenesis	0.0194	25
	Extracellular Matrix	Extracellular matrix organization	1.10E-07	75
		Collagen formation	0.00139	22

	organisation	Activation of Matrix Metalloproteinases	0.00182	17
		Degradation of the extracellular matrix	0.00182	31
		Collagen biosynthesis and modifying enzymes	0.0039	15
		Matrix Metalloproteinases	0.0162	13
		Assembly of collagen fibrils and other multimeric structures	0.038	12
	Digestive system	Bile secretion	0.031	26
	Disease	Maturity onset diabetes of the young	0.0103	13
	Haemostasis	Platelet degranulation	0.0347	20
		Response to elevated platelet cytosolic Ca2+	0.0368	21
	Muscle	Striated Muscle Contraction	0.00986	16
		Muscle contraction	0.0144	17
	Neuronal response	Serotonergic synapse	0.0454	29
	Transmembrane transport of small molecules	Membrane Trafficking	0.0039	15
		Transmembrane transport of small molecules	0.035	110
14 days	Signalling	Signal Transduction	0.00988	225
		Signalling by GPCR	0.0215	169
		GPCR downstream signalling	0.0284	157
	Xenobiotics metabolism	Cytochrome P450 - arranged by substrate type	0.0215	15
		Biological oxidations	0.0215	29
		Phase 1 - Functionalization of compounds	0.0284	19
	Metabolism of proteins	Retinol metabolism	0.0232	20
		Metabolism of proteins	0.0346	31
	Immune response	Innate Immune System	0.0215	28
		Immune System	0.0222	58
		Natural killer cell mediated cytotoxicity	0.0346	15
	Extracellular	Extracellular matrix organization	0.0215	23

	Matrix organisation			
	Digestive system	Bile secretion	0.0408	16
	Haemostasis	Response to elevated platelet cytosolic Ca2+	0.0346	11
	Cellular Processes	Regulation of actin cytoskeleton	0.0222	19
		XPodNet - protein-protein interactions in the podocyte expanded by STRING	0.0279	76
		Focal Adhesion	0.0404	13
21 days	Signalling	Signal Transduction	5.65E-10	80
		Signalling by GPCR	7.69E-07	47
		GPCR downstream signalling	6.43E-06	42
		GPCR ligand binding	0.000203	20
		Class A/1 (Rhodopsin-like receptors)	0.00061	17
		G alpha (i) signalling events	0.00105	16
		Non-odorant GPCRs	0.00193	14
		Signalling by NGF	0.00193	13
		Olfactory transduction	0.00193	34
		G Protein Signalling Pathways	0.0034	12
		GPCRs, Class A Rhodopsin-like	0.00601	11
		HIF-1 signalling pathway	0.0101	10
		Peptide ligand-binding receptors	0.014	11
		NGF signalling via TRKA from the plasma membrane	0.014	9
		Wnt signalling pathway	0.014	9
	Metabolism of proteins	Metabolism	1.10E-06	39
		Metabolism of carbohydrates	0.000444	16
		Glycosaminoglycan metabolism	0.014	9
		MPS I - Hurler syndrome	0.014	9

		MPS II - Hunter syndrome	0.014	9
		MPS IIIA - Sanfilippo syndrome A	0.014	9
		MPS IIIB - Sanfilippo syndrome B	0.014	9
		MPS IIIC - Sanfilippo syndrome C	0.014	9
		MPS IIID - Sanfilippo syndrome D	0.014	9
		MPS IV - Morquio syndrome A	0.014	9
		MPS IV - Morquio syndrome B	0.014	9
		MPS VI - Maroteaux-Lamy syndrome	0.014	9
		MPS VII - Sly syndrome	0.014	9
		MPS IX - Natowicz syndrome	0.014	9
		Mucopolysaccharidoses	0.014	9
		Metabolism of proteins	0.0238	8
	Immune response	Innate Immune System	2.91E-10	41
		Immune System	5.65E-10	63
		Adaptive Immune System	1.49E-07	30
		Cell adhesion molecules (CAMs)	1.27E-05	22
		HTLV-I infection	0.000244	17
		Cytokine-cytokine receptor interaction	0.000723	15
		T Cell Receptor Signalling Pathway	0.00193	13
		Leukocyte transendothelial migration	0.00193	13
		Epstein-Barr virus infection	0.00193	13
		B Cell Receptor Signalling Pathway	0.00601	11
		Tuberculosis	0.00601	11
		Herpes simplex infection	0.00601	11
		DAP12 interactions	0.0101	10
		Chemokine signalling pathway	0.0101	10
		Toll-Like Receptors Cascades	0.0101	10

		Signalling by the B Cell Receptor (BCR)	0.0101	10
		Fc epsilon receptor (FCERI) signalling	0.0101	10
		NF-kappa B signalling pathway	0.0101	10
		DAP12 signalling	0.014	9
		Toll Like Receptor 4 (TLR4) Cascade	0.014	9
		Complement and coagulation cascades	0.014	9
		Natural killer cell mediated cytotoxicity	0.014	9
		Hematopoietic cell lineage	0.014	9
		Viral myocarditis	0.014	9
		Chemokine signalling pathway	0.0238	8
		Complement and Coagulation Cascades	0.0238	8
		Pertussis	0.0238	8
		Immunoregulatory interactions between a Lymphoid and a non-Lymphoid cell	0.0238	8
		Cytokine Signalling in Immune system	0.0238	8
		T cell receptor signalling pathway	0.0238	8
		Downstream Signalling Events Of B Cell Receptor (BCR)	0.0238	8
		Influenza A	0.0238	8
		B cell receptor signalling pathway	0.0238	8
	Cancer signalling	PI3K-Akt signalling pathway	0.000244	17
		Pathways in cancer	0.000244	17
		Transcriptional misregulation in cancer	0.000444	16
		Viral carcinogenesis	0.000723	15
		Integrin-mediated Cell Adhesion	0.0034	12
		Proteoglycans in cancer	0.00601	11
		Cell Cycle	0.0101	10
		MicroRNAs in cancer	0.014	9

		Cell Cycle, Mitotic	0.0238	8
		MAPK signalling pathway	0.0238	8
		Ras signalling pathway	0.0252	15
	Extracellular Matrix organisation	Extracellular matrix organization	6.94E-06	23
	Disease	Disease	6.56E-06	29
	Haemostasis	Hemostasis	7.69E-07	27
		Calcium Regulation in the Cardiac Cell	0.00193	13
		Platelet activation, signalling and aggregation	0.00193	13
		Calcium signalling pathway	0.0034	12
		Potassium Channels	0.0101	10
		Cell surface interactions at the vascular wall	0.014	9
	Cellular Processes	XPodNet - protein-protein interactions in the podocyte expanded by STRING	5.87E-07	38
		Regulation of actin cytoskeleton	2.35E-05	21
		Endocytosis	0.000504	20
		Phagosome	0.00122	14
		PodNet- protein-protein interactions in the podocyte	0.00122	14
		Focal adhesion	0.00122	14
		Regulation of Actin Cytoskeleton	0.00193	13
		Focal Adhesion	0.0034	12
		Gene Expression	0.0292	10
	Muscle	Myometrial Relaxation and Contraction Pathways	0.0238	8
		Vascular smooth muscle contraction	0.0238	8
	Neuronal response	Developmental Biology	0.000174	18
		Neuronal System	0.000244	25

		Axon guidance	0.000244	17
		Neuroactive ligand-receptor interaction	0.000723	15
		Neurotransmitter Receptor Binding And Downstream Transmission In The Postsynaptic Cell	0.00122	14
		Transmission across Chemical Synapses	0.00256	19
		Osteoclast differentiation	0.0101	10
		Cholinergic synapse	0.014	9
		L1CAM interactions	0.014	9
		Glutamatergic synapse	0.0238	8
	Transmembrane transport of small molecules	Transmembrane transport of small molecules	0.00457	18
	Other	Circadian entrainment	0.014	9
